# Further Validation of Quantum Crystallography Approaches

**DOI:** 10.3390/molecules26123730

**Published:** 2021-06-18

**Authors:** Monika Wanat, Maura Malinska, Anna A. Hoser, Krzysztof Woźniak

**Affiliations:** 1 Biological and Chemical Research Centre, Department of Chemistry, University of Warsaw, 101 Żwirki i Wigury, 02-089 Warszawa, Poland; mwanat@uw.edu.pl (M.W.); m.malinska@uw.edu.pl (M.M.); a.hoser@uw.edu.pl (A.A.H.); 2 College of Inter-Faculty Individual Studies in Mathematics and Natural Sciences (MISMaP), University of Warsaw, 2C Stefana Banacha, 02-097 Warszawa, Poland

**Keywords:** multipole model, normal mode refinement, Hirshfeld atom refinement, transferable aspherical atom model, charge density

## Abstract

Quantum crystallography is a fast-developing multidisciplinary area of crystallography. In this work, we analyse the influence of different charge density models (i.e., the multipole model (MM), Hirshfeld atom refinement (HAR), and the transferable aspherical atom model (TAAM)), modelling of the thermal motion of hydrogen atoms (anisotropic, isotropic, and with the aid of SHADE or NoMoRe), and the type of radiation used (Mo Kα and Cu Kα) on the final results. To achieve this aim, we performed a series of refinements against X-ray diffraction data for three model compounds and compared their final structures, geometries, shapes of ADPs, and charge density distributions. Our results were also supported by theoretical calculations that enabled comparisons of the lattice energies of these structures. It appears that geometrical parameters are better described (closer to the neutron values) when HAR is used; however, bonds to H atoms more closely match neutron values after MM or TAAM refinement. Our analysis shows the superiority of the NoMoRe method in the description of H-atom ADPs. Moreover, the shapes of the ADPs of H atoms, as well as their electron density distributions, were better described with low-resolution Cu Kα data in comparison to low-resolution Mo Kα data.

## 1. Introduction

Quantum crystallography is a multidisciplinary “volcanic“ area of crystallography that combines X-ray diffraction experiments with quantum mechanics. According to an excellent review article about quantum crystallography [1], there are four definitions of this field: The first is linked to wavefunction and density matrix modelling; however, this definition excludes most charge density studies based on the multipole model or maximum entropy methods. The second definition expands the range of acceptable methods, as it assumes that quantum crystallography is based on studies of quantum mechanical functions in crystals. The third definition treats crystals as quantum objects, and assumes that only quantum mechanics may explain the phenomena that are found during studies of crystalline materials. The fourth definition is the widest, treating quantum crystallography as a multidisciplinary field that allows for studies of materials other than solid-state crystals. Although the above definitions are not precise, most workers in the field currently seem to agree that quantum crystallography should also include charge density analysis based on both experimental data and theoretical calculations, meaning that quantum crystallography is crystallography beyond the independent atom model (IAM).

In this work we will analyse four methods of refinement of electron density—the multipole model (MM) [2], the transferable aspherical atom model (TAAM) [3,4,5,6,7,8], Hirshfeld atom refinement (HAR) [9], and a combination of HAR and normal mode refinement (NoMoRe) [10,11]—all of which, from our point of view, are quantum crystallography methods.

MM refinement of high-resolution X-ray diffraction data leads to a different quantitative distribution of electron density to the commonly used IAM, as it takes the asphericity of atomic charge density into consideration. TAAM is based on a multipole model derived from the pseudoatom databanks [3,6,8], whereas Hirshfeld atom refinement is based on wavefunction calculations. Both of these models—TAAM and HAR—enable the reconstruction of theoretical/experimental charge density. Moreover, these two models enable refinement against the low-resolution X-ray data (i.e., d > 0.8 Å), as opposed to the multipole model, which requires high-resolution X-ray data (i.e., d < 0.5 Å). Another approach to refinement was proposed in 2016 by Hoser and Madsen [10], who introduced the NoMoRe method. This method is based on DFT calculations of frequencies at the Γ point. These results are used for the calculation of ADPs. The atomic coordinates are refined, while structure factors—as well as *w*R_2_—are calculated. Then, selected frequencies are optimized to minimize *w*R_2_. Due to adjustment of the values of frequencies to X-ray diffraction data, the calculations of ADPs are repeated until convergence. NoMoRe results may be combined with any of the previously described methods.

In our previous study [12] concerning quantum crystallography methods, we showed the influence of the application of the HAR and TAAM models on refinements against Cu Kα data collected with a resolution of 0.8 Å. We showed that HAR (anisotropic, isotropic, and SHADE variants) and TAAM (SHADE and isotropic variants) refinements resulted in reliable final structures. This was supported by reasonable values of crystallographic agreement factors and the quality of geometric parameters. Although the results of refinement against Cu Kα data generally agreed with refinements against high-resolution Mo Kα data, some systematic trends were also observed in the final structural models. These effects included systematically higher ADPs as well as smaller maxima and larger minima of residual electron density for Cu Kα than for Mo Kα data. However, based on those results, we were not able to identify which systematic errors arose from a difference in data resolution, and which resulted from the radiation source.

In this work, we discuss the influence of the applied model of electron density (Section 3.2), the approach to modelling hydrogen atom ADPs (Section 3.3), and the source of radiation (Section 3.4) on the parameters of charge density distribution. Some of these aspects were discussed in previous studies; for instance, the influence of H-atom ADPs on charge density results [13,14], the application of low-resolution Cu Kα data [12,15,16], and the differences between quantum crystallography models [17,18,19,20,21,22]. In this complementary work, we consider the effects of the refinement of hydrogen atoms with the NoMoRe method, as well as the influence of the source of radiation (with the same resolution).

**Figure 1:**
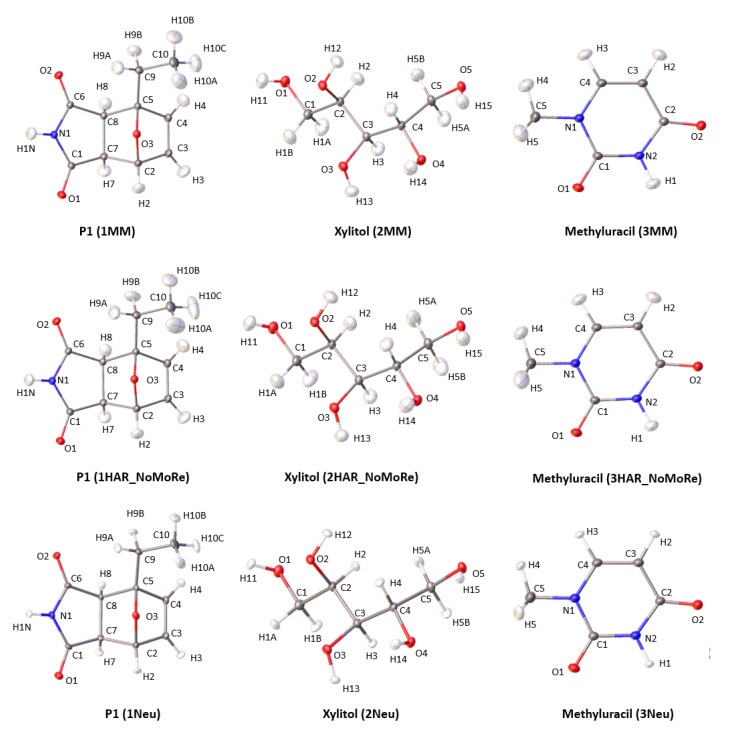
Labelling of atoms and visualisation and comparison of atomic ADPs in molecular structures of the studied compounds **1**, **2**, and **3** obtained with the MM and HAR–NoMoRe refinements and neutron diffraction. Anisotropic atomic displacement ellipsoids are drawn at the 50% probability level.

We performed our analyses using structures of the following model compounds: P1—i.e., 10-oxa-4-azatricyclo(5.2.1.02,6)dec-8-ene-3,5-dione—(**1**); xylitol (**2**); and methyluracil (**3**) (Figure 1).

## 2. Materials and Methods

### 2.1. Crystallographic Data

High-resolution Mo Kα X-ray diffraction data for single crystals of compounds **1** [23] and **3**, as well as Cu Kα X-ray diffraction data for crystals of compounds **1**, **2**, and **3**, were collected by us [12], whereas the high-resolution Mo Kα X-ray diffraction data for **2** were collected by Madsen et al. [24]. Neutron data used as a reference in this work were collected by us (**1**), [12], by Madsen et al. (**2**), [25] and by McMullan et al. (**3**) [26] (Figure 1).

### 2.2. Multipole Model Refinement

Starting from TAAM structures, with the ADPs of H atoms estimated by using the SHADE3 server (CSD deposition numbers: 2008296 (**1**); 2008302 (**2**) [12]), the high resolution X-ray diffraction data for **1** and **2** were refined using MoProSuite [27]. In the refinement, the following parameters were refined consecutively: valence populations (P_val_); multipoles (P_lm_, i.e., dipoles, quadrupoles, octupoles, and hexadecapoles for oxygen atoms); and expansion and contraction parameters (kappa and kappa’). After each block refinement of kappas, the xyz and U_ij_ parameters were refined, while the ADPs of H atoms were estimated by the SHADE3 server [28,29] and applied as constraints. Moreover, the X–H distances were extended to the mean neutron values as restraints, with a standard deviation of 0.001 [30] (Figure 1).

Starting from the TAAM structure, with the ADPs of H atoms estimated by using the SHADE3 server ([12]; CSD deposition number: 2008274), the model parameters (P_val_, P_lm_, kappa, and kappa’) for **3** were refined using XD2016 against high-resolution data [31]. In the refinement, the following parameters were refined consecutively: valence populations (P_val_); multipoles (P_lm_, i.e., dipoles, quadrupoles, octupoles and, for oxygen atoms, hexadecapoles); and kappa and kappa’. After each block refinement of kappas, the xyz and U_ij_ parameters were refined. The ADPs of H atoms were constrained with the aid of the SHADE3 server, and X–H distances were extended to the neutron values and applied as constraints. In the first cycle of refinement, the site symmetry restrictions based on symmetric spherical harmonics, including additional restrictions from UBDB, were used. In further cycles, additional restrictions from UBDB were neglected (Figure 1). Details of the combination of the MM approach with NoMoRe for compound **1** are available in Supplementary Section S1. This approach was also previously reported [11].

### 2.3. Hirshfeld Atom Refinement and the Transferable Aspherical Atom Model

In this work, we analyse HAR and TAAM (procedures described in Supplementary Figures S1 and S2) refinements of high-resolution Mo Kα X-ray data for **1**, **2**, and **3**, which were previously described in [12]. The HAR method was applied using Tonto, version 18.01.30. Calculations were performed using the DFT method, B3LYP functional, and cc-pVDZ basis set, using a full range of reflections. TAAM parameters based on atom types were recognized by the LSDB program [32]. Atom population, kappa, and kappa’ parameters were derived from the UBDB2016 [3]. During the refinement with TAAM, the X–H distances were elongated to the neutron values and refined as the restraints, with an s.u. value of 0.001Å. In HAR, the distances between heavy atoms and hydrogen atoms were refined freely [9,33]. Moreover, for the SHADE approach for TAAM and HAR, the ADPs of hydrogen atoms were constrained to the values generated by the SHADE3 server. All of the structures were deposited in the Cambridge Structural Database with the following deposition numbers: anisotropic HAR refinements: 2008277 (**1**), 2008301 (**2**), and 2008288 (**3**); isotropic HAR refinements: 2008275 (**1**), 2008304 (**2**), and 2008287 (**3**); HAR_SHADE refinements: 2008276 (**1**), 2008302 (**2**), and 2008286 (**3**); TAAM_SHADE refinements: 2008296 (**1**), 2008282 (**2**), and 2008274 (**3**); and isotropic TAAM refinements: 2008295 (**1**), 2008283 (**2**), and 2008273 (**3**). TAAM and HAR refinements with the same approaches were repeated for the low-resolution Mo Kα data (**1-cutoff**, **2-cutoff**, **3-cutoff**). The cut-off to the resolution equal to 0.8 Å was applied with the aid of WinGX Software [34]. Details are available in Table S1. These results were compared with the results of Cu Kα X-ray data refinements, as previously described in [12], and deposited in the Cambridge Structural Database: anisotropic HAR refinements: 2008290 (**1-Cu Kα**), 2008300 (**2-Cu Kα**), and 2008698 (**3-Cu Kα**); isotropic HAR refinements: 2008181 (**1-Cu Kα**), 2008298 (**2-Cu Kα**), and 2008699 (**3-Cu Kα**); HAR_SHADE refinements: 2008289 (**1-Cu Kα**), 2008299 (**2-Cu Kα**), and 2008700 (**3-Cu Kα**); TAAM_SHADE refinements: 2008294 (**1-Cu Kα**), 2008281 (**2-Cu Kα**), and 2008271 (**3-Cu Kα**); and isotropic TAAM refinements: 2008293 (**1-Cu Kα**), 2008280 (**2-Cu Kα**), and 2008225 (**3-Cu Kα**).

The CIF files resulting from new refinements and related to the present paper were deposited in the CSD, and carry the following identification numbers—for high-resolution Mo Kα data: 2081779 (**P1_MM**), 2081783 (**P1_HAR_NoMoRe**), 2081794 (**xylitol_MM**), 2081791 (**xylitol_HARNoMoRe**), 2081799 (**methyluracil MM**), and 2081800 **(methyluracil_HAR_NoMoRe**); low-resolution (d = 0.8 Å) Mo Kα data: 2081785 (**P1_TAAM_iso**), 2081784 (**P1_TAAM_Shade**), 2081782 (**P1_HAR_Shade**), 2081781 (**P1_HAR_iso**), 2081780 (**P1_HAR_ani**), 2081793 (**xylitol_TAAM_iso**), 2081792 (**xylitol_TAAM_Shade**), 2081790 (**xylitol_HAR_iso**), 2081789 (**xylitol_HAR_ani**), 2081788 (**xylitol_HAR_Shade**), 2081798 (**methyluracil TAAM_Shade**), 2081797 (**methyluracil TAAM_iso**), 2081796 (**methyluracil HAR_ani**), 2081795 (**methyluracil HAR_iso**), and 2081801 (**methyluracil HAR_Shade**).

Additionally, in this work we used the results of the IAM refinement for all three crystal structures for comparison; the structure parameters were refined against high-resolution Mo Kα data using Olex2 Software. The structures were solved with SHELXS [35] and refined with SHELXL [36], and then deposited in the CSD: 2008269 (**1**), 2085455 (**2**), and 2008180 (**3**).

### 2.4. HAR with ADPs from the NoMoRe (Normal Mode Refinement) Method

X-ray Mo Kα data for compounds **1**, **2**, and **3** were subjected to NoMoRe refinement. The initial frequencies and normal mode coordinates for **1**, **2**, and **3** were calculated with the DFT method using the B3LYP functional and 6-31G(d, p) basis set in CRYSTAL17 [37,38]. Prior to the above operations, the structures were optimized (coordinates only). The obtained frequencies and normal mode vectors were used for the calculation of ADPs. Based on the obtained ADPs, the structure factors and *w*R_2_ were calculated using SHELXL [36]. In further steps, the previously calculated frequencies were optimized to minimize the *w*R_2_ and used for the next cycle of ADP calculations [10]. For NoMoRe refinement details, see the Supplementary Materials (Figure S3, Table S2).

In HAR, the wavefunctions calculated in all refinements were obtained using the DFT method, applying the BLYP functional and the cc-pVDZ basis set. In accordance with previous studies [17,33,39], the cluster size of the atomic charges and dipoles was set to a standard size of 8 Å. For all refinements, the full range of reflections (excluding those with negative intensities) was used [17]. The ADPs of hydrogen atoms obtained via the NoMoRe method were used for the Hirshfeld atom refinement (HAR) as fixed constraints. In the HAR, only the positions of all atoms and ADPs of heavy atoms were refined (Figure 1).

### 2.5. ADP Analysis

A similarity index (S = 100(1-R_12_)) [40] was used to analyse the differences between hydrogen atom ADP values for two models, where R_12_ is the overlap of probability density functions p_1_ and p_2_ of the analysed ADP tensors. R_12_ is defined as below:

(1) $$R_{12}=\int {\left[p_{1}\left(x\right)p_{2}\left(x\right)\right]}^{1∕2}d^{3}x=\frac{2^{3/2}{\left({\det\;U}_{1}^{-1}U_{2}^{-1}\right)}^{1/4}}{[{\left(\det\left(U_{1}^{-1}+U_{2}^{-1}\right)\right]}^{1/2}}$$

where U_1_ and U_2_ represent ADP tensors. The mean similarity index ($\bar{S}$) value is the average value of the similarity index values obtained for a molecule. The $\bar{S}$. value is used as a reference. Based on our studies [12,17] and literature data [11,40], we propose that good agreement is achieved for an $\bar{S}$ value equal to or less than 1. Notably, the $\;\bar{S}$ value is a reliable parameter for the analysis of ADPs with high accordance. On the other hand, this parameter can be sometimes misleading [41]. For instance, visible differences between model and neutron ADPs (i.e., elongated ellipsoids of the analysed model ADPs) are observed where the $\bar{S}$ value is higher than 1, and the presence of the non-positive definite (NPD) ADPs leads to an $\bar{S}$ value higher than 6.

**Figure 2:**
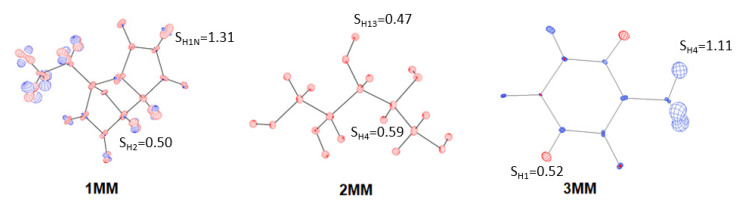
Differences between ADP values resulting from neutron and MM refinements for **1**, **2**, and **3**. PEANUT plots for HAR_aniso, HAR_NoMoRe, HAR_SHADE, and TAAM_SHADE are available in the Supplementary Materials (Figures S27–S29). Scale is equal to 2.0. The colour red represents ADPs larger than neutron ADPs, whereas blue represents ADPs smaller than neutron ADPs. Selected values of similarity index (S_HX_, where x is an atom number) were added to the plots.

Visualisation of differences between the ADPs of all atoms was performed using the PEANUT software [42]. Two target structures—the neutron, and the analysed model—were overlaid with a two-step overlay algorithm. A graphical representation of the differences between the ADPs was obtained, and these differences are presented (Figure 2).

### 2.6. Theoretical Computations

The lattice energy was calculated by subtracting the electronic energy of a single molecule in the gas phase from the electronic energy of the unit cell in the crystal divided by the number of molecules. Basis set superposition error (BSSE) was also accounted for. Using CRYSTAL17 [37,38], we performed calculations of the bulk energy of the crystal structures and the molecular energy of the molecules in the gas phase. The applied level of theory was DFT/B3LYP, with DFT-D3(ATM) dispersion correction [43] and the cc-pVDZ basis set, in accordance with our previous calculations [12,17]. Moreover, ghost atoms used for BSSE estimation were selected at up to 4 Å distance from the central molecule [44]. LSDB was used for normalisation of hydrogen atom positions for the IAM model [3,32]. All calculations were performed without geometry optimization. As a reference, we used previously obtained results of lattice energy obtained from neutron data. The results for the neutron data were calculated in two variants—with, and without geometry optimization [12].

## 3. Results and Discussion

### 3.1. Refinement Models

**Figure 3:**
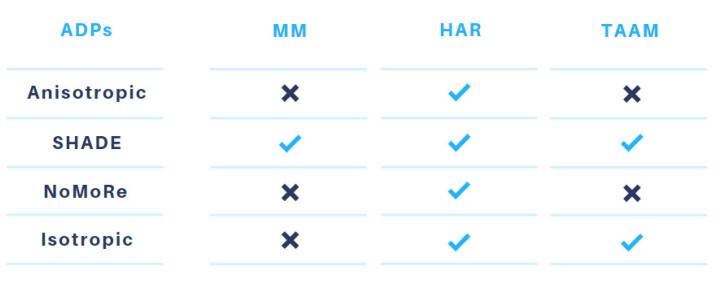
Summary of the modelling of H atoms in the MM, HAR, and TAAM methods.

In this work, we considered refinements of high-resolution X-ray Mo Kα data for three compounds: **1** (P1), **2** (xylitol), and **3** (methyluracil). The X-ray data were refined with the following models: MM, four variants of HAR, and two variants of TAAM (which differed in hydrogen atom ADP treatment). These approaches are summarized in Figure 3. The ADPs of H atoms were refined (HAR_aniso), not refined or constrained (HAR_SHADE, HAR_NoMoRe, TAAM_SHADE), or the H atoms were described by the isotropic displacement parameters and constrained to the appropriate value derived from the heavy atom ADPs (HAR_iso, TAAM_iso). The results of the applied models and modelling of H atoms are analysed in Section 3.2 and Section 3.3, respectively. Additionally, in Section 3.4, we discuss the influence of the type of radiation used on the final results. We compare the results of HAR and TAAM refinements against X-ray Mo Kα (**1-cutoff**, **2-cutoff**, **3-cutoff**) and Cu Kα (**1-Cu Kα**, **2-Cu Kα**, **3-Cu Kα**) data with the same resolution (i.e., d = 0.8Å).

According to the results, all methods analysed here have similar levels of user-friendliness; however, combination of HAR and NoMoRe requires more steps, as the NoMoRe approach must be performed before HAR in order to obtain the ADPs. This is also the most time-consuming method, as NoMoRe requires calculations performed in CRYSTAL17 (our calculations last up to 5457559 CPU seconds). The exact time of the performed calculations depends on the size of the molecules. For analysed compounds, one cycle of MM/TAAM refinement takes ca. 1–20 CPU seconds (up to one wall clock minute). TAAM requires up to six cycles, whereas MM requires ca. 20–30 cycles, and one cycle of HAR require 2350–7340 CPU seconds (ca. 40–150 wall clock minutes). Anisotropic and isotropic HAR calculations required one cycle, NoMoRe two cycles, and SHADE two or three cycles. Therefore, the TAAM and MM methods are less time-consuming in comparison to HAR.

### 3.2. Validation of Refinement Models

#### 3.2.1. Agreement Factors and Residual Density

The final structures, as well as the statistical parameters of the performed refinements, are presented in Table 1. Although the differences between parameters describing the quality of the refinement of the models are insignificant, most values of R (F > 2σ(F)) are smaller for HAR than for TAAM refinement, because TAAM has smaller flexibility than HAR. Moreover, the values of the R (F > 2σ(F)) and *w*R(F^2^) parameters are larger for the isotropic approaches than for the others.

For all refinements, the goodness-of-fit (GoF) parameters are less than or greater than 1. For the refinements of **1** and **3**, these values are higher, whereas for **2** they are lower. The explanation of these effects may be found in the analysis of normal probability plots. As we indicated in our previous paper [12], the standard uncertainty (s.u.) of the reflection intensities is mostly underestimated for **1** and **3**, while being overestimated for **2**. The errors in fitting s.u. values are reflected in the GoF being larger than 1 for the too small s.u. values and smaller than 1 for the too large s.u. values [45].

The higher values of the maxima and minima of residual densities for **3** (MM and TAAM) might result from the positioning of methyluracil (except for the H5 hydrogen atom) in the symmetry plane. This positioning of methyluracil introduces many site symmetry restrictions for the Z coordinate, U_13_, U_23_, and selected multipole parameters. In HAR, this problem does not occur, as the refinements are performed on the cluster of molecules instead of the molecule with Z’ = 0.5.

**Figure 4:**
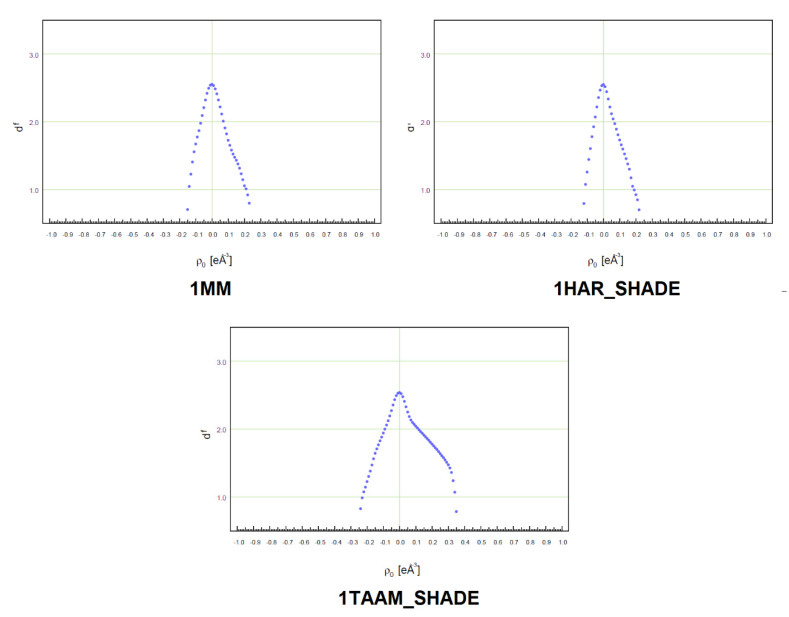
Fractal dimension plots for MM, HAR_SHADE, and TAAM_SHADE refinements of **1**. Fractal dimension plots for all analysed compounds and methods are available in the Supplementary Materials (Figures S5–S7).

Further inspection of the residual density maps (Figures S2–S4), as well as the fractal dimension plots (Figure 4 and Figures S5–S7), allows for analysis of the quality of fit parameters of a given model to the experimental structure factors (HAR is based on refinement against |F_o_|, whereas MM and TAAM against |F_o_^2^|). The most significant differences between the final residual electron densities obtained with these methods of refinement are illustrated on the fractal dimension plots (Figure 4 and Figures S5–S7). The fractal dimension plots of HAR are narrower and have more regular, parabolic shapes than the fractal dimension plots for TAAM refinements. The values of Δρ = Δρ_max_ − Δρ_min_ are higher for TAAM than for MM (up to 0.36 eÅ^−3^), but Δρ of MM is also higher in comparison to HAR (up to 0.47 eÅ^−3^). Systematic differences occur for fractal dimension plots of HAR_aniso—which are slightly narrower than for HAR_NoMoRe, HAR_SHADE, and HAR_iso—which may result from the highest number of refined parameters. To judge the extent to which the residual distribution is featureless, we have added the analysis of maximum of the fractal dimension plot—the d^f^ (0) values—for each refinement, which should be 3.0 for the ideal parabolic distribution and close to 2.7 for the experimental data. The highest d^f^ (0) scores are obtained after MM refinements. After cutting the resolution of the XRD data to 0.81 Å, the d^f^ (0) values are lower among all refinements, as expected. However, TAAM refinement for cut-off data has higher values than HAR, showing that maps after TAAM have less features. Moreover, the values for HAR refinement are significantly lower, showing that maps after HAR have features that we do not see on residual density map.

#### 3.2.2. Geometric Parameters

**Figure 5:**
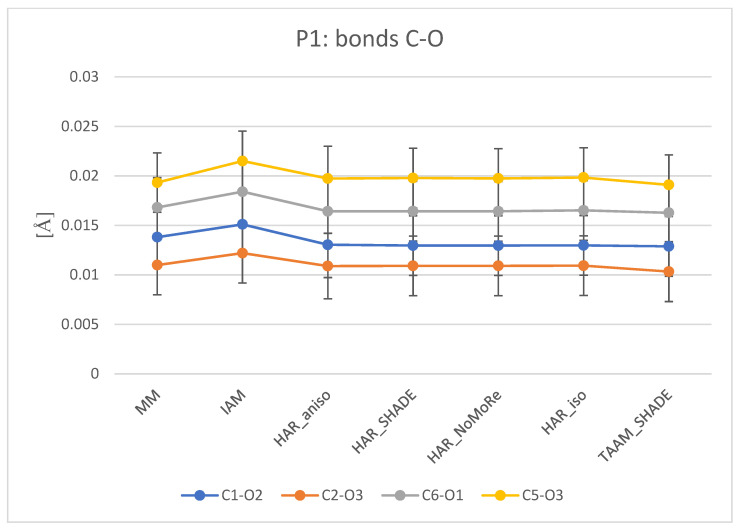
Comparison of the O–C bonds for IAM, MM, HAR, and TAAM refinements of **1**. The values on the plot represent the difference between the analysed model and the neutron data (in Å). Plotlines have no physical meaning, but aid in visual analysis. For each plot, estimated standard deviations within +/−1 ESD are included. For comparison, plots of O–C bonds for **2** and **3** are available in the Supplementary Materials (see Figure S8).

Analysis of geometrical parameters (Figures S8–S13) reveals that the bonds between the carbon atoms and the non-hydrogen atoms (i.e., C, N, and O) are described properly by all of the analysed methods—MM, HAR, and TAAM (Figure 5 and Figures S8–S10). The differences for bond lengths obtained from the analysed models and neutron data do not exceed 0.035 Å (for **1**) and 0.007 Å (for **2** and **3**), whereas estimated standard deviations (ESDs) for neutron bonds between non-hydrogen atoms do not exceed 0.003 Å. The results of refinements for different variants of displacement parameters with the same charge density model are similar. Significantly, some higher deviations were observed for bond lengths with hydrogen atoms. This shows that the treatment of hydrogen atoms reflects itself only in the geometry of bonds to hydrogen atoms.

**Figure 6:**
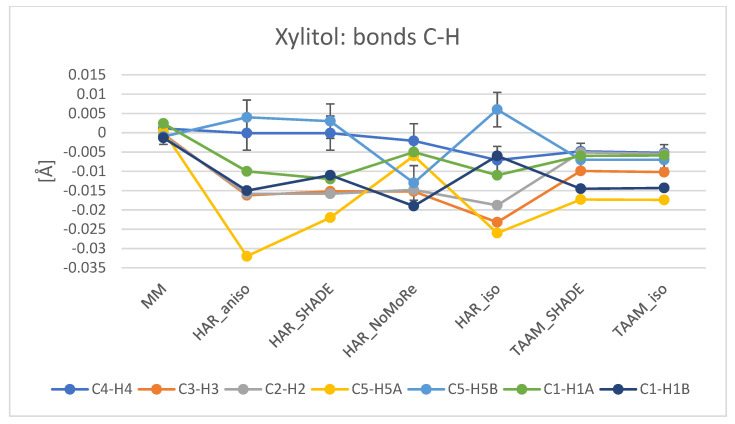
Comparison of C–H bonds for MM, HAR, and TAAM refinements of **2**. The values on the plot represent the difference between a given analysed model and the neutron data. Plotlines have no physical meaning, but aid in visual analysis. For clarity, +/−1 ESD intervals were added to selected bonds (C4–H4 and C5–H5B). Restraints were applied for MM and TAAM refinements. Plots for the comparison of C–H bonds in **1** and **3** are available in Supplementary Figure S11.

Analysis of bond lengths to hydrogen atoms proved that these bonds for MM and TAAM are closer to the neutron values than in the case of HAR (Figure 6 and Figures S11–S13). Notably, for MM and TAAM, restraints were applied for X–H bonds, which increased agreement with the neutron data. For the non-hydrogen atoms, differences between bond lengths obtained with the analysed model and those from the neutron data were greater. These differences generally did not exceed 0.035 Å, whereas ESDs for neutron bonds with hydrogen atoms did not exceed 0.002 Å (for **2** and **3**) and 0.007 Å (for **1**). Unfortunately, it is difficult to find any trends for X–H bonds in the case of HAR_aniso, HAR_SHADE, HAR_NoMoRe, and HAR_iso refinements. For instance, the O–H bonds are largely better described (closer to neutron values) by HAR_aniso than in the case of other variants of refinements. However, this result was not repeated for the N–H bonds, where HAR_SHADE supplied bonds for **1** slightly better than HAR_aniso, but for **3**, HAR_aniso supplied bonds significantly closer to the neutron values than HAR_SHADE. In comparison, results obtained with TAAM_SHADE and TAAM_iso were in accord. This shows that the way one treats hydrogen atoms has a strong influence on the final results of HAR refinement. Therefore, in the refinements such as those presented in this work, the TAAM method is more reliable than HAR for a description of bond lengths, due to the application of restraints.

**Figure 7:**
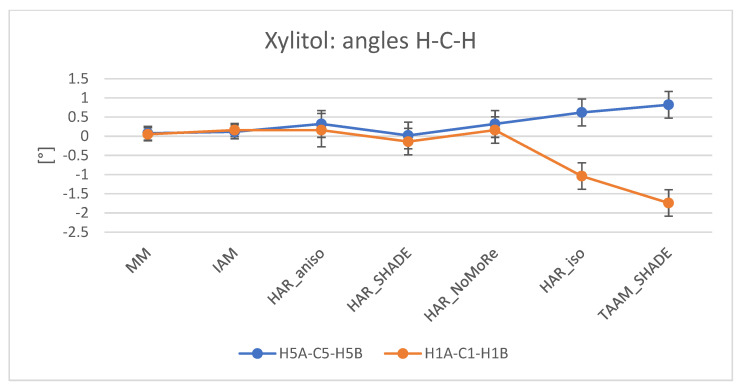
Comparison of H–C–H angles for MM, IAM, HAR, and TAAM refinements of **2**. Plot values represent the difference between values obtained with the analysed model and the neutron data, in degrees. Plotlines have no physical meaning, but aid in visual analysis. For each plot, estimated standard deviations were added. Plots for the comparison of H–C–H angles for **1** and **3** are available in Supplementary Figure S26.

Analysis of angles gives similar results (Figure 7 and Figures S14–S26). We did not apply restraints for angles. The values of angles without hydrogen atoms (OCC, OCN, CNC, CCC, NCC, COC, and NCN) were similar to the neutron ones, and the differences did not exceed 0.4°, which is well below the level of expected error. The differences observed for values of angles with hydrogen atoms (OCH, CNH, CCH, HCH, COH, NCH, and CNH) were larger, reaching up to 7°. Generally, the angles were closer to the neutron values with MM refinement. Similarly to the analysis of bonds, comparison of the results of the HAR_aniso, HAR_SHADE, HAR_NoMoRe, and HAR_iso variants of refinement did not indicate which method achieved the highest agreement with neutron data. However, most results from HAR_iso reflect lower accordance with the neutron data than the results of the other HAR refinements. Although TAAM_SHADE and TAAM_iso give similar results in bond analysis, this trend was repeated only for angles with hydrogen atoms. Neither TAAM_SHADE nor TAAM_iso was shown to give a description of the angles closer to the neutron values. Moreover, comparison of the TAAM and HAR results revealed that the HAR results exhibited smaller differences with respect to the neutron values than the TAAM results. Therefore, both the HAR and MM methods were more reliable than TAAM for the description of valence angles.

#### 3.2.3. Lattice Energy

The last step of validation for the final models concerns comparison of the lattice energy calculation results (Table 2 and Table S3). For this validation, we intentionally omitted geometric optimization, as we intended to compare the results of lattice calculations for the final geometry of a particular model. The geometric optimization was applied only for the neutron data, which were used as a reference (Table 2). However, the results without geometric optimization for neutron data are also available (Table S3). The X–H bond lengths for IAM were extended to the neutron values, as the positions of H atoms had a significant influence on the final results. This was also shown by previous analyses of lattice energy calculations for IAM and quantum crystallography methods [12,46].

Our results show that the most accurate and precise values of lattice energy in accordance with the neutron data are obtained with MM. Further analysis shows that the differences between the SHADE and isotropic variants of the HAR and TAAM models are in agreement, except for HAR in the case of **2**. This last disagreement may result from the high number of degrees of freedom for the xylitol molecule. Differences between HAR_aniso and HAR_NoMoRe, HAR_SHADE, or HAR_iso suggested that the HAR_aniso and HAR_NoMoRe refinements may be better suited to describing lattice energy than HAR_SHADE or HAR_iso.

### 3.3. Results of H Atom ADP Estimation

**Figure 8:**
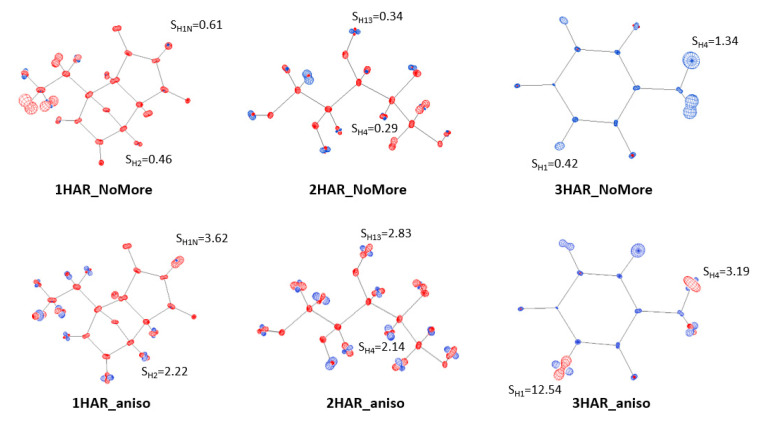
Differences between the ADPs of the neutron data and the analysed models for HAR_NoMoRe and HAR_aniso refinements of **1**, **2**, and **3**. A two-step overlay algorithm and 2.0 scale were applied using PEANUT software. Selected similarity index values (S_HX_, where x is an atom number) were added to the plots. PEANUT plots for MM, HAR_aniso, HAR_NoMoRe, HAR_SHADE, and TAAM_SHADE are available in the Supplementary Materials (Figures S27–S29).

In order to analyse the ADPs of hydrogen atoms, we calculated the similarity index S and mean similarity index $\bar{S}$ (Table 3 and Tables S3–S5), and prepared PEANUT plots (i.e., graphical representations of differences between the ADPs of the neutron model and an analysed model (Figure 8 and Figures S27–S29)). The ADPs in our models were obtained via anisotropic refinement (HAR_aniso) and application of ADPs from SHADE (MM, HAR_SHADE, and TAAM) or NoMoRe (HAR_NoMoRe). ADPs obtained via the SHADE method (MM, HAR, and TAAM) resulted in similar values of the similarity index, with $\bar{S}$ ranging from 0.7 to 0.9. The only exception was the HAR_SHADE refinement of **3**, for which $\bar{S}$ was equal to 0.5 (1) (Table 3 and Tables S4–S6). According to our criteria, all ADPs fell into the category of very good or good agreement with the neutron values of ADPs.

The high agreement between the SHADE and neutron ADPs results from the inclusion of internal motion derived from the library based on the neutron experiments in SHADE2. This was also the only case where the application of SHADE resulted in the closest agreement with the neutron values. However, further analysis of HAR_NoMoRe revealed that this method was even better. The mean similarity indexes for **1**, **2**, and **3** were small, and equal to $\bar{S}$_1_ = 0.6 (1), $\bar{S}$_2_ = 0.4 (1), and $\bar{S}$_3_ = 0.6 (2), respectively. None of the analysed methods improved results for **1** or **2**, whereas the lowest values of the mean similarity indices for **3** were obtained with HAR_SHADE ($\bar{S}$_3_ = 0.5 (1)). Therefore, the methods using SHADE (MM, HAR, and TAAM) could not be considered the best methods for describing ADPs. According to our analysis, the HAR_NoMoRe and HAR_SHADE approaches resulted in the best fitting of H atom ADPs. However, HAR_NoMoRe was slightly better (the ADPs were closer to the neutron values) than HAR_SHADE (with the sole exception of **3**; see Table 3). The worst was HAR_aniso, as the mean similarity index values were higher than 1, with high values of standard error of the mean ($\bar{S}$_1_ = 1.4 (3), $\bar{S}$_2_ = 2.2 (4), $\bar{S}$_3_ = 4 (2)) (Table 3, Figure 8).

### 3.4. Influence of Data Resolution on the Final Results

X-ray data for **1**, **2**, and **3** analysed in this work were collected up to resolutions of 0.46 Å, 0.41 Å, and 0.45 Å, respectively. Here, we discuss the influence of the radiation wavelength on the final results. However, for the sake of comparison with the Cu Kα results, the Mo Kα X-ray data discussed in this chapter were trimmed to a resolution equal to d = 0.8 Å (**1-cutoff**, **2-cutoff**, and **3-cutoff**). We performed refinements against **1-cutoff**, **2-cutoff**, and **3-cutoff** data using the HAR_aniso, HAR_iso, HAR_SHADE, TAAM_SHADE, and TAAM_iso models of electron density. Analysis of the data quality shows that the R_int_ and R_sigma_ are smaller and I/sigma(I) is larger for **1-Cu Kα** and **2-Cu Kα** in comparison to **1-cutoff** and **2-cutoff** (excluding R_int_ for **2-cutoff** as the data is merged); the opposite is true for the comparison of **3-Cu Kα** and **3-cutoff**. (Table S1).

All results appear to be quite reasonable based on the statistical parameters (Tables S7–S9), obtained geometry, and ADP values. 

#### 3.4.1. Geometric Analysis

Geometric analysis revealed that, in general, bond lengths between heavy atoms (i.e., C–O, C–N, C–C (Figures S30–S32)) were similar for the Mo Kα and Cu Kα X-ray diffraction data with resolution d = 0.8 Å. However, the use of Cu Kα data results in most of the geometrical parameters of these bonds being slightly closer to the neutron values. Particularly for **3**, the Cu Kα X-ray results seemed to better mimic the neutron O–C bonds after the HAR refinement. However, this is not a general rule. For instance, after TAAM refinement, the Mo Kα results better described the N–C bonds.

Analysis of bonds to hydrogen atoms (Figures S33–S35) revealed that significant differences are observed only for HAR refinements, which is to be expected, as in the TAAM refinements the lengths of the bonds to hydrogen atoms were extended to the neutron values.

Quite a complex situation appears when comparing the results of the application of different wavelengths—no clear trends that would show which form of radiation would be better to use are evident. For instance, the O–H bonds seemed to be better described (closer to the neutron values) by the Mo Kα results, while the N–H bonds were best described by the Cu Kα results for **1** and by the Mo Kα results for **3**. For valence angles (see Figures S36–S48), some angles have higher accordance with the neutron values with Mo Kα (i.e., the C–O–C angle of **1** and the O–C–C angle of **3**), and others with Cu Kα (i.e., the O–C–H and N–C–C angles of **1**). However, for most of the cases (i.e., C–C–H for **1**, **2**, and **3**), the angles are described equally well by data obtained with both sources of radiation. Therefore, we suggest no preferred radiation wavelength.

#### 3.4.2. ADP Analysis

Analysis of ADPs obtained from the refinements against **1-cutoff**, **2-cutoff**, and **3-cutoff** data performed with PEANUT (Figure S49) revealed that, in general, for low-resolution datasets, the ADPs of heavy atoms were larger than the neutron ADPs. Additionally, it appears that H atom ADPs obtained with low-resolution Cu Kα radiation were closer to neutron H atom ADPs than those obtained with low-resolution Mo Kα X-ray diffraction data—with the exception of HAR_ SHADE and TAAM_ SHADE—for **1-cutoff** and **1-Cu Kα** (Table 4 and Tables S10–S12). ADPs obtained with the high-resolution X-ray diffraction data are closer to the neutron data than those obtained with low-resolution X-ray diffraction data. Therefore, we can confirm that precise and accurate information concerning ADP values is available in the high-resolution region. For low-resolution data, more precise and accurate ADP values were obtained with the refinement against Cu Kα data.

#### 3.4.3. Analysis of Residual Density

**Figure 9:**
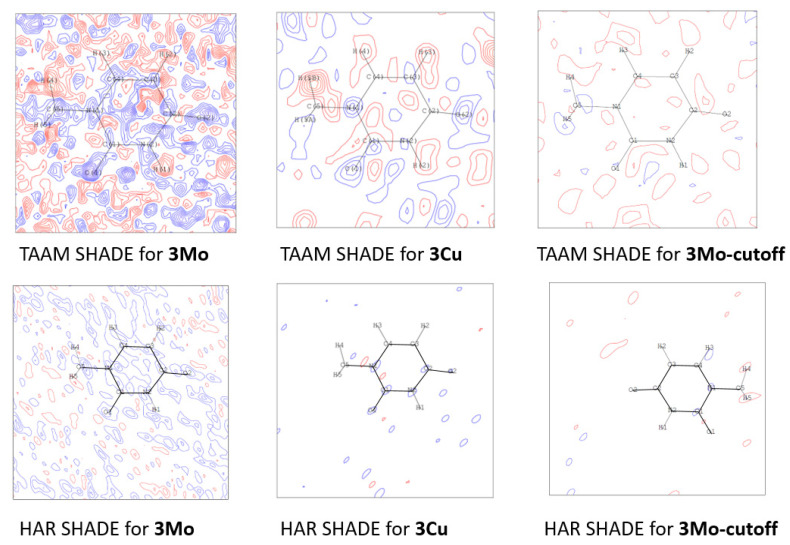
Residual density maps for HAR_SHADE and TAAM_SHADE refinements of **3Mo**, **3Cu**, and **3-cutoff**. Maps are presented with contour levels with intervals of ±0.05 eÅ^−3^. All residual density maps for the analysed compounds are available in the Supplementary Materials (Figures S2–S4).

Further analysis of the fractal plots (Figures S5–S7) and residual density maps (Figures S2–S4) revealed the presence of larger maxima and smaller minima for the residual densities obtained from the high-resolution Mo Kα dataset than for those obtained from the Cu Kα and Mo Kα low-resolution data (Figure 9). Moreover, larger maxima and smaller minima for the residual densities were observed for the **3-cutoff** and **1-cutoff** datasets (only in the case of HAR_aniso refinement) than for **3-Cu Kα** and **1-Cu Kα**. Some opposite relations are observed for the **1-cutoff** (HAR_SHADE and TAAM_SHADE refinements) and **2-cutoff** dataset refinements, which may also be related to the data quality (Section 3.4.1, Table S1). Interestingly, the tendencies observed for **1-cutoff**/**1-Cu Kα** and **3-cutoff**/**3-Cu Kα** were in accordance with the results for ADPs. Therefore, the closer ADP values were to the neutron values, the better the fitting of the model of electron density to the experimental structure factors. This confirms that lower quality descriptions of the shapes of ADP values influence the quality of the electron density. However, there are some exceptions, such as **2-cutoff**/**2-Cu Kα**, which could be associated with the presence of systematic errors in these datasets (parallel strips of positive and negative regions on residual density maps).

## 4. Conclusions

We analysed various aspects of the final structures refined with quantum crystallography methods of electron density refinement, such as MM, HAR (four variants of H-ADP modelling: anisotropic, isotropic, SHADE, and NoMoRe), and TAAM (two variants modelling H-ADPs: SHADE and isotropic). Our results show that:

- According to agreement/discrepancy factors, all methods lead to reasonable models of electron density (see Section 3.2.1).
- Analysis of geometrical parameters revealed that HAR better supplies valence angles closer to the neutron values, whereas bonds, particularly with H atoms, seem to be better described by MM and TAAM (at least in the case of the studied compounds). This may result from restraints applied in those two models. Similar results were also obtained for lattice energies (see Section 3.2.2).
- The HAR model requires restraints to the X–H bonds (see Section 3.2.2).
- Validation of the used models presented in this work revealed that the application of particular treatments of H atoms may have a significant influence on the final results. These effects include:
- The NoMoRe approach may be indicated as the superior method of treatment of hydrogen atom thermal motion (see Section 3.2.2).

ADP values obtained with the NoMoRe method and combined with HAR were in good agreement with the ADP values obtained from the neutron data (see Section 3.3). The HAR model may be supported by ADP constraints from SHADE or NoMoRe (see Section 3.3).

- Isotropic refinement of H atoms in HAR led to some of the worst geometric modelling results, whereas isotropic refinement of H atoms in TAAM refinement supplied similar results to the application of SHADE (see Section 3.2.2).
- Anisotropic and NoMoRe approaches mostly resulted in lattice energies closer to the reference neutron values (see Section 3.2.3).
- Finally, we analysed model changes based on refinement against low-resolution Mo Kα and Cu Kα X-ray diffraction data. Results obtained with both wavelengths led to reliable geometry of the final structures (see Section 3.4.1); however, some systematic effects were observed in the ADP values:
- ADPs of heavy atoms obtained with Mo Kα X-ray diffraction data were systematically closer to the ADPs obtained from neutron diffraction, and smaller than those obtained with Cu Kα data (see Section 3.4.2).
- H atoms’ ADP values obtained with Cu Kα data were closer to the neutron ADP values than those obtained with Mo Kα data (0.8 Å) (see Section 3.4.2).
- A better description of ADP values was also reflected in a better fit of the model to the experimental electron density (see Section 3.4.3).

Therefore, if a crystal diffracts only to a resolution of ca. 0.8 Å, we recommend collecting XRD data using copper radiation and the application of any model more advanced than IAM.

## Figures and Tables

**Table 1:** Statistical parameters of MM, HAR, and TAAM refinements for **1**, **2**, and **3** against high-resolution Mo Kα data.

Compound	Parameters	MM	HAR Aniso	HAR SHADE	HAR NoMoRe	HAR Iso	TAAM SHADE	TAAM Iso
**1**	R(F > 2σ(F))	0.013	0.015	0.015	0.015	0.017	0.015	0.018
	*w*R(F^2^)	0.043	0.045	0.045	0.045	0.051	0.050	0.057
	# of reflections	9849	9828	9826	9828	9828	9849	9849
	# of fit parameters	160	226	160	160	160	160	171
	chi^2^	*n*/*a*	2.35	2.39	2.41	2.94	*n*/*a*	*n*/*a*
	Goodness of fit	1.35	1.53	1.55	1.55	1.72	1.58	1.77
	Δρ_max_	0.22	0.20	0.22	0.21	0.22	0.33	0.33
	Δρ^min^	−0.16	−0.15	−0.15	−0.15	−0.16	−0.21	−0.33
		MM	HAR aniso	HAR SHADE	HAR NoMoRe	HAR iso	TAAM SHADE	TAAM iso
**2**	R(F > 2σ(F))	0.021	0.021	0.021	0.021	0.022	0.018	0.023
	*w*R(F^2^)	0.029	0.030	0.031	0.031	0.034	0.035	0.038
	# of reflections	9779	9776	9776	9776	9776	9779	9779
	# of fit parameters	127	199	127	127	127	127	139
	chi^2^	*n*/*a*	0.50	0.52	0.51	0.62	*n*/*a*	*n*/*a*
	Goodness of fit	0.67	0.71	0.72	0.72	0.79	0.82	0.89
	Δρ_max_	0.24	0.16	0.15	0.15	0.15	0.24	0.32
	Δρ^min^	−0.27	−0.19	−0.19	−0.19	−0.19	−0.32	−0.46
		MM	HAR aniso	HAR SHADE	HAR NoMoRe	HAR iso	TAAM SHADE	TAAM iso
**3**	R (F > 2σ(F))	0.037	0.038	0.039	0.039	0.039	0.051	0.031
	*w*R(F^2^)	0.057	0.060	0.061	0.061	0.066	0.074	0.070
	# of reflections	3113	3113	3113	3113	3113	3113	3113
	# of fit parameters	56	88	66	66	66	55	60
	chi^2^	*n*/*a*	1.86	1.91	1.93	1.91	*n*/*a*	*n*/*a*
	Goodness of fit	1.24	1.36	1.38	1.39	1.38	1.56	1.51
	Δρ_max_	0.42	0.16	0.17	0.17	0.17	0.49	0.44
	Δρ^min^	−0.33	−0.17	−0.17	−0.17	−0.17	−0.42	−0.29

**Table 2:** Differences between lattice energies calculated based on the final models and the neutron data. Calculations for charge density models were performed without geometric optimization. Calculations for models obtained from neutron measurements were performed with geometric optimization. Results are shown in kJ/mol. Calculations were performed using CRYSTAL17 at the DFT/B3LYP level of theory and applying the DFT-D3(ATM) dispersion correction, using the cc-pVDZ basis set.

Model	1	2	3
MM	1.28	11.63	1.72
IAM	3.05	12.35	0.25
HAR_aniso	2.75	13.65	1.70
HAR_Shade	2.74	14.16	2.37
HAR_NoMoRe	2.46	14.44	2.08
HAR_iso	2.54	15.34	2.38
TAAM_Shade	1.39	11.67	2.94
TAAM_iso	1.33	11.37	2.94

**Table 3:** Mean similarity index ($\bar{S}$) with standard error of the mean for hydrogen atom ADPs of **1**, **2**, and **3** in comparison to the neutron results for MM, HAR_aniso, HAR_SHADE, HAR_NoMoRe, or TAAM_SHADE refinements.

Compound	MM	HAR_Aniso	HAR_SHADE	HAR_NoMoRe	TAAM_SHADE
**1**	0.7 (2)	1.4 (3)	0.7 (1)	0.6 (1)	0.7 (1)
**2**	0.8 (2)	2.2 (4)	0.8 (2)	0.4 (1)	0.8 (2)
**3**	0.9 (4)	4 (2)	0.5 (1)	0.6 (2)	0.7 (3)

**Table 4:** The mean similarity index $\bar{S}$ obtained from refinements against the high-resolution datasets **1**, **2**, and **3**, and low-resolution datasets **1-cutoff**, **2-cutoff**, **3-cutoff**, **1-Cu Kα**, **2-Cu Kα**, and **3-Cu Kα** for HAR_aniso, HAR_SHADE, and TAAM_SHADE refinements for H atoms. Neutron data were used as the reference. Detailed tables containing the similarity index values for each hydrogen atom of **1**, **2**, and **3** (Tables S4–S6), and **1-cutoff**, **2-cutoff**, and **3-cutoff**, respectively, are available in the Supplementary Materials (Tables S10–S12). The values obtained for **1-Cu Kα**, **2-Cu Kα**, and **3-Cu Kα** were published in our previous studies [12].

Compound	HAR_Aniso	HAR_SHADE	TAAM_SHADE
**1**	1.43	0.70	0.72
**1-cutoff**	2.55	0.79	0.87
**1-Cu Kα**	2.13	0.96	1.14
**2**	2.16	0.78	0.79
**2-cutoff**	11.18 *	4.2	4.21
**2-Cu Kα**	10.19 *	1.19	1.34
**3**	4.14	0.47	0.73
**3-cutoff**	6.64	2.22	3.12
**3-Cu Kα**	5.80	0.74	1.18

*: The S value for the NPD hydrogen atom was omitted in the calculation of the mean value.

## Data Availability

Not applicable.

## Supplementary Materials

The following supporting materials are available online: Table S1: Statistical parameters of high- and low-resolution Mo Kα data; Table S2: Normal mode refinement details; Table S3: Lattice energy calculations of **1**, **2**, and **3**; Tables S4–S6: Similarity index ($\bar{S}$) and mean similarity index ($\bar{S}$) calculated for **1**, **2**, and **3**; Tables S7–S9: Statistical parameters of **1-cutoff**, **2-cutoff**, and **3-cutoff**; Tables S10–S12: Similarity index ($\bar{S}$) and mean similarity index ($\bar{S}$) calculated for **1-cutoff**, **2-cutoff**, and **3-cutoff**; Figure S1: ORTEP view of the molecular structures obtained with the neutron, Mo Kα, and Cu Kα data; Figures S2–S4: Residual density maps for **1**, **1-cutoff**, **2**, **2-cutoff**, **3**, and **3-cutoff**; Figures S5–S7: Normal probability plots for **1**, **1-cutoff**, **2**, **2-cutoff**, **3**, and **3-cutoff**; Figures S8–S13: Comparison of bonds for **1**, **2**, and **3**; Figures S14–S26: Comparison of angles for **1**, **2**, and **3**; Figures S27–S29: Differences between the ADPs of the neutron data and the analysed models for **1**, **2**, and **3**; Figures S30–S35: Comparison of bonds for **1-cutoff**, **2-cutoff**, and **3-cutoff**; Figures S36–S48: Comparison of angles for **1-cutoff**, **2-cutoff**, and **3-cutoff**; Figure S49: Difference between the ADPs of the neutron data and the analysed models for **1-cutoff**, **2-cutoff**, and **3-cutoff**; Section S1: Refinement of MM with use of the ADPs for H atoms obtained from the NoMoRe approach (Table S14: Statistical parameters for MM_NoMoRe refinement of **1**); Figure S50: ORTEP view of **1** refined with the MM_NoMoRe model; Figure S51: Fractal dimension plots and residual density maps for MM (SHADE, NoMoRe) refinements of **1**; Figure S52: Comparison of the C–H bonds of **1**, including MM_NoMoRe refinement.

## Sample Availability

Samples of the compounds **1**, **2** and **3** are available from the authors.
