# Supplementary material for: Further Validation of Quantum Crystallography Approaches
Source: Molecules. 2021 Jun 18;26(12):3730. doi: 10.3390/molecules26123730 (PMC8233966; doi:10.3390/molecules26123730)
Supplement: Supplementary file 1 [file molecules-26-03730-s001.zip › molecules-1237761-supplementary.pdf]

# Supporting information

## Further Validation of Quantum Crystallography Approaches

**Monika Wanat[1,2], Maura Malinska[1], Anna A. Hoser[1] and Krzysztof Wozniak[1]***

[1]Biological and Chemical Research Centre, Department of Chemistry, University of Warsaw, 101 Żwirki i Wigury, Warszawa, 02-089, Poland

[2]College of Inter-Faculty Individual Studies in Mathematics and Natural Sciences (MISMaP), University of Warsaw, 2C Stefana Banacha, Warszawa, 02-097, Poland

Correspondence email: kwozniak@chem.uw.edu.pl

**List of content:**


- Hirshfeld Atom Refinement procedure
- Transferable Aspherical Atom Model procedure
- Normal Mode Refinement procedure
- Fractal dimension plots procedure
- **Table S1** Statistical details of **1**, **1-cutoff**, **2**, **2-cutoff**, **3** and **3-cutoff** structures.
- **Table S2** Normal Mode Refinements details
- **Table S3** Lattice energy calculations of **1**, **2** and **3**.
- **Tables S4-S6** Similarity index (S) and mean similarity index ($\bar{S}$ ) calculated for **1**, **2** and **3**.
- **Tables S7-S9** Statistical parameters of **1-cutoff**, **2-cutoff** and **3-cutoff**.
- **Tables S10-S12** Similarity index (S) and mean similarity index ($\bar{S}$ ) calculated for **1-cutoff**, **2-cutoff** and **3-cutoff**.
- **Tables S13** Summary of the $d^f$ maximum of the fractal dimension plots.
- **Figure S1** ORTEP view of the molecular structures obtained with the neutron, MoKα and CuKα data.
- **Figures S2-S4** Residual density maps for **1**, **1-cutoff**, **2**, **2-cutoff**, **3** and **3-cutoff**.
- **Figures S5-S7** Fractal dimension plots for **1**, **1-cutoff**, **2**, **2-cutoff**, **3** and **3-cutoff**.
- **Figures S8-S13** Comparison of bonds for **1**, **2** and **3**.

- **Figures S14-S26** Comparison of angles for **1**, **2** and **3**.
- **Figure S27-S29** Difference between ADPs of neutron and analysed model for **1**, **2** and **3**.
- **Figure S30-S35** Comparison of bonds for **1-cutoff**, **2-cutoff**, and **3-cutoff**.
- **Figure S36-S48** Comparison of angles for **1-cutoff**, **2-cutoff** and **3-cutoff**.
- **Figure S49** Difference between ADPs of neutron and analysed model for **1-cutoff**, **2-cutoff** and **3-cutoff**.
- **Section S1: Combination of Multipole Model with Normal Mode Refinement**
- **References**

**Hirshfeld Atom Refinement**

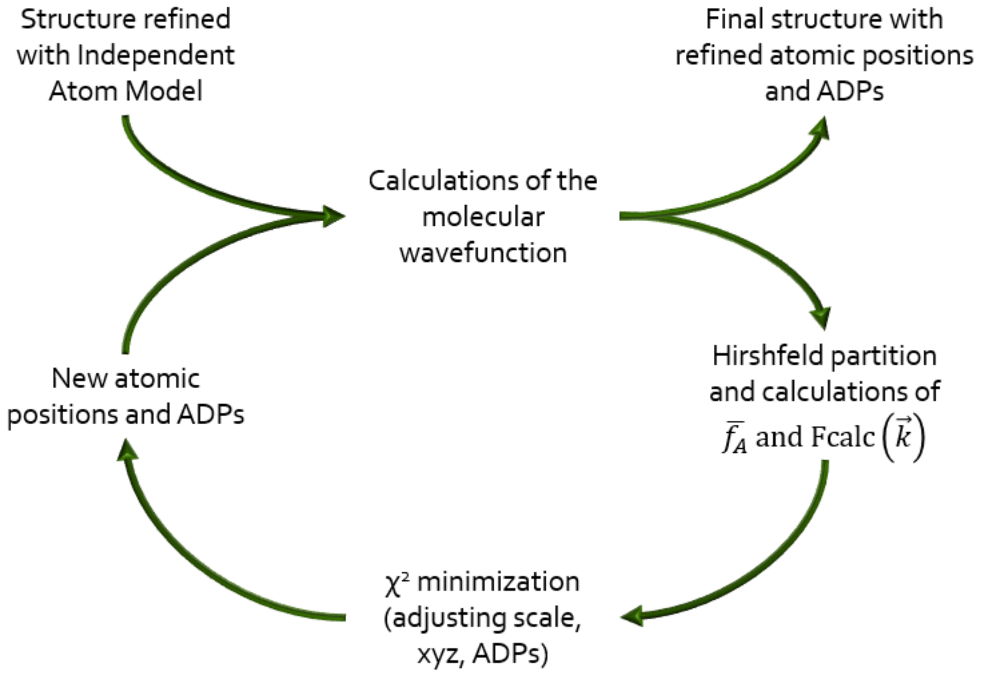


***Scheme S1*** *Hirshfeld Atom Refinement scheme.* [1]

**Procedure:**

After application of Independent Atom Model ( to obtain starting geometry, ADPs, experimental structure factors), the wavefunction for cluster of molecules is calculated. In the next step, the molecular electron density function is divided into atomic contributions (Hirshfeld partition), and atomic scattering factors are calculated. Then, minimization is performed by adjusting parameters such as scale, atomic positions and ADPs between calculated and observed structure factors. As a result, the new atomic positions are obtained and used in next cycle of wavefunction calculations. This cycle is repeated till convergence and as a final step, the final structure with refined atomic positions and ADPs is obtained. The final structure is validated with analysis of atomic positions, residual and deformation density maps, fractal dimension plots and statistical parameters including value of $\chi^2$.

Procedure based on reference [1]

**Transferable Aspherical Atom Model**

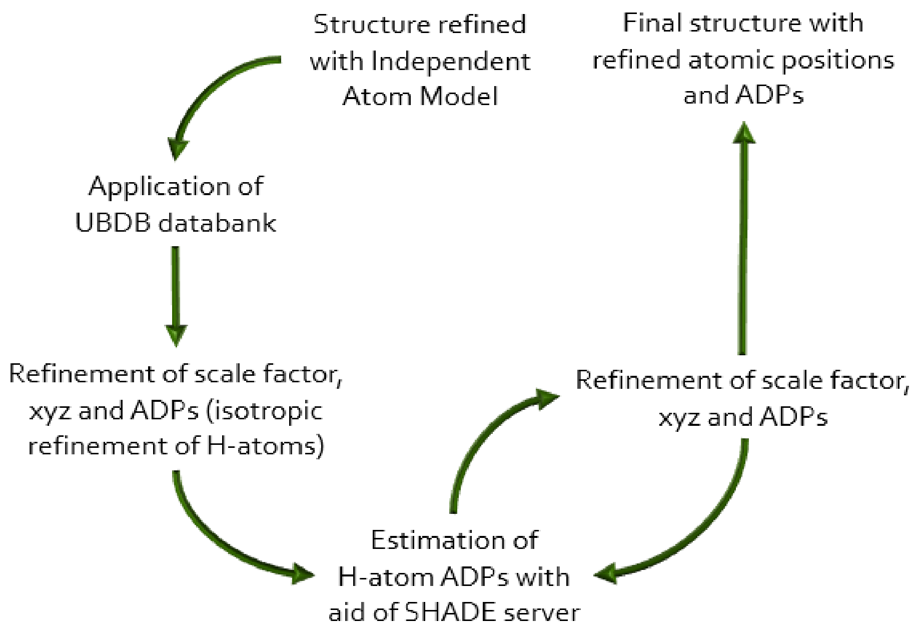


***Scheme S2*** *Transferable Aspherical Atom Model scheme based on references* [2–7]

**Procedure:**

Firstly, the structure is refined with Independent Atom Model. Then, with the aid of LSDB Software (Volkov, A. et al., J. Phys. Chem. A, 2004, 108, 4283–4300), the pseudoatom parameters are transferred from UBDB Pseudoatom Databank. Multipole populations and contraction/expansion parameters in the UBDB are obtained in fitting procedure in reciprocal lattice of multipole model and electron density parameters are obtained with quantum-mechanical calculations. The atom types used in UBDB are based on mean electron density parameters for various pseudoatoms with similar topology of bonds in the model compounds, for which the geometries are obtained from experimental X-ray data.

After the assignment of electron density parameters for structure refined with IAM, the scale factor, atomic positions and ADPs of heavy atoms (anisotropic) as well as hydrogen atoms (isotropic) are refined.

In the next step, the H-atoms of ADPs with the aid of SHADE server. Constraints obtained from SHADE are based on the sum of internal and external motions. Internal motions are derived from library containing results of neutron diffractions experiments, whereas external motions are obtained using TLS (Translation-Libration-Screw) model introduced by Schomaker and Trueblood in 1968. Refinements of scale factors,

atomic positions with ADPs are proceed till convergence. The final structure is validated with analysis of atomic positions, residual and deformation density maps, fractal dimension plots and statistical parameters.

Procedure based on references [2–7]

## Normal Mode Refinement

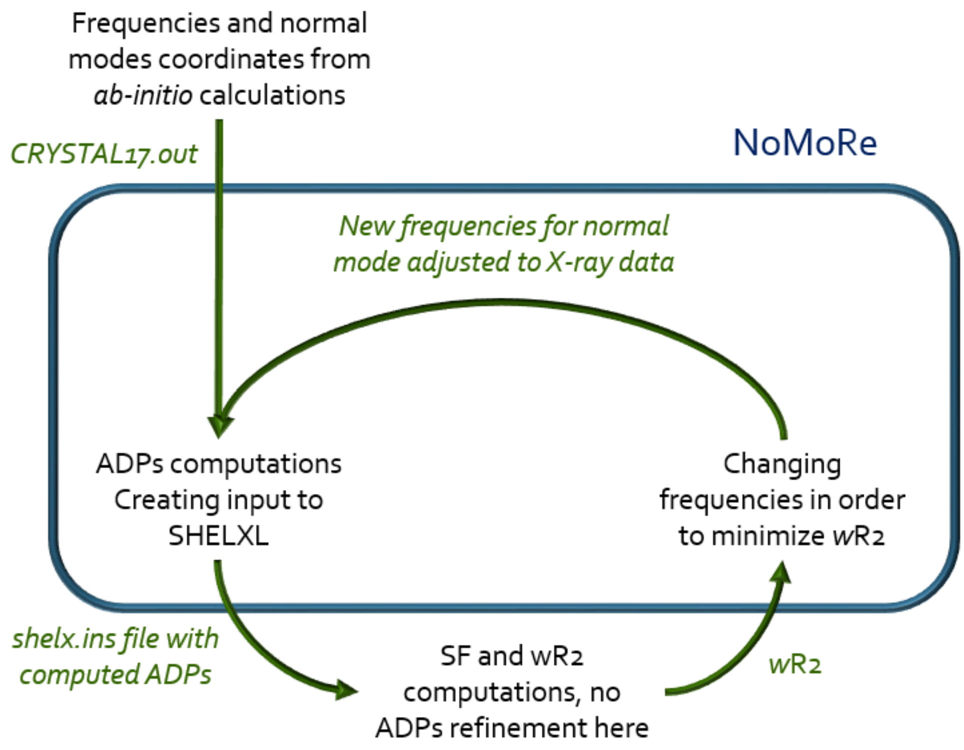


**Scheme S3** *Normal Mode Refinement scheme based on references [8,9]*

**Procedure:**

The first step of NoMoRe procedure is performing calculations in CRYSTAL17 to obtain frequencies at the Γ point. Based on this calculation, the ADPs are calculated. The atomic coordinates are refined whereas structure factors, as well as *w*R2, are calculated. Then, selected frequencies are optimized to achieve minimization of *w*R2. Due to adjusted the values of frequencies to X-ray diffraction data, the calculations of ADPs are repeated. ADPs obtained with NoMoRe approach may be combined with other quantum crystallography models.

Procedure based on references [8,9]

**Fractal dimension plots**

The fractal dimension is described with the formula:

$$d^f(\rho_0;\varepsilon) = \frac{\log(N(\varepsilon))}{\log(\frac{1}{\varepsilon})}$$

$$\frac{1}{\varepsilon} = \sqrt[3]{3(n_x - 1)(n_y - 1)(n_z - 1)}$$

whereas:

$\rho_0$ is residual electron density,

$d^f(\rho_0)$ is fractal dimension calculated for the specific point of $\rho_0$ (*e.g.* $\rho_0$=0.05 eÅ$^{-3}$) and

$n_x, n_y, n_z$ are dimensions of the used grid.

The fractal dimension plots show condition of residual density distribution. The shape of the plot is parabolic and related to the Gaussian distribution, therefore the deviations indicate the presence of systematic errors. Also, the width of fractal dimension plot $\Delta\rho_0$ enable evaluation of residual density distribution. Moreover, the $\rho_0$ is related with additional descriptors which may indicate reason of presence of systematic errors. For instance, values of the gross residual electrons ($e_{gross}$ [e]) may indicate presence of errors related to the applied density model, data processing or presence of noise.

**Table S1** Statistical details of **1**, **1-cutoff**, **1-CuKα**, **2**, **2-cutoff**, **2-CuKα 3**, **3-cutoff** and **3-CuKα** structures.

| | Rint | Rsigma | I/sigma(I) |
|---|---|---|---|
| **1-cutoff** | 0.0607 | 0.0141 | 71.0 |
| **1** | 0.0296 | 0.0254 | 39.3 |
| **1-CuKα** | 0.0290 | 0.0075 | 133.8 |
| **2-cutoff** | Data merged from CSD | 0.0190 | 52.7 |
| **2** | Data merged from CSD | 0.0317 | 31.6 |
| **2-CuKα** | 0.0464 | 0.0152 | 65.8 |
| **3-cutoff** | 0.0260 | 0.0081 | 123.8 |
| **3** | 0.0557 | 0.0310 | 32.2 |
| **3-CuKα** | 0.0470 | 0.0103 | 97.0 |

**Table S2** Refined frequencies and *w*R2 for Normal Mode Refinements for **1**, **2** and **3**.

| | Refined frequencies | *w*R2 |
|---|---|---|
| **1** | 1-33 | 0.0922 |
| **2** | 1-23 | 0.0699 |
| **3** | 1-16 | 0.1567 |

**Table S3** Differences between lattice energy of analysed models and neutron. Calculations of all data were performed without geometry optimization. Results are shown in kJ/mol. Calculations were performed using CRYSTAL17 at the DFT/B3LYP level of theory and applying the DFT-D3(ATM) dispersion correction, using cc-pVDZ basis set.

| | **1** | **2** | **3** |
|---|---|---|---|
| MM | 1.12 | 0.58 | 1.05 |

| | | | |
|---|---|---|---|
| IAM | 2.49 | -2.98 | 2.03 |
| HAR aniso | 2.59 | 9.39 | 1.04 |
| HAR Shade | 2.59 | 9.91 | 1.71 |
| HAR NoMoRe | 2.31 | 10.18 | 1.41 |
| HAR iso | 2.39 | 11.08 | 1.71 |
| TAAM Shade | 1.24 | 7.41 | 2.28 |
| TAAM iso | 1.18 | 7.11 | 2.28 |

**Table S4** Similarity index (S) and mean similarity index ($\bar{S}$ ) calculated for **1**.

| | MM | HAR_ani | HAR_SHADE | HAR_NoMoRe | TAAM |
|---|---|---|---|---|---|
| mean overlap ($\bar{S}$ ) | 0.71 | 1.43 | 0.70 | 0.57 | 0.72 |
| H9A | 0.49 | 0.95 | 0.48 | 0.54 | 0.49 |
| H9B | 1.43 | 0.87 | 1.41 | 0.72 | 1.48 |
| H1N | 1.31 | 3.62 | 1.24 | 0.61 | 1.26 |
| H8 | 0.40 | 0.40 | 0.38 | 0.3 | 0.45 |
| H10A | 0.77 | 0.85 | 0.82 | 1.24 | 0.84 |
| H10C | 1.03 | 2.00 | 1.00 | 1.19 | 0.97 |
| H10B | 0.75 | 0.22 | 0.88 | 0.45 | 0.89 |
| H2 | 0.50 | 2.22 | 0.51 | 0.46 | 0.51 |
| H3 | 0.35 | 2.43 | 0.33 | 0.29 | 0.33 |
| H7 | 0.47 | 1.66 | 0.43 | 0.36 | 0.48 |
| H4 | 0.28 | 0.51 | 0.27 | 0.17 | 0.25 |

**Table S5** Similarity index (S) and mean similarity index ($\bar{S}$ ) calculated for **2**.

| | MM | HAR_ani | HAR_SHADE | HAR_NoMoRe | TAAM |
|---|---|---|---|---|---|
| mean overlap ($\bar{S}$ ) | 0.80 | 2.16 | 0.78 | 0.36 | 0.79 |
| H1A | 1.90 | 3.47 | 1.77 | 0.55 | 1.73 |
| H5A | 0.95 | 0.98 | 0.91 | 0.73 | 0.93 |
| H5B | 0.87 | 2.46 | 0.86 | 0.37 | 0.91 |

| | | | | | |
|---|---|---|---|---|---|
| H11 | 0.64 | 1.17 | 0.65 | 0.33 | 0.70 |
| H12 | 1.01 | 1.28 | 0.99 | 0.35 | 1.06 |
| H13 | 0.47 | 2.83 | 0.43 | 0.34 | 0.50 |
| H14 | 0.25 | 0.6 | 0.3 | 0.1 | 0.28 |
| H15 | 0.78 | 4.7 | 0.78 | 0.4 | 0.78 |
| H1B | 1.63 | 2.52 | 1.64 | 0.29 | 1.57 |
| H2 | 0.22 | 2.21 | 0.2 | 0.21 | 0.23 |
| H3 | 0.30 | 1.53 | 0.26 | 0.38 | 0.27 |
| H4 | 0.59 | 2.14 | 0.53 | 0.29 | 0.58 |

**Table S6** Similarity index (S) and mean similarity index ($\bar{S}$ ) calculated for **3**.

| | MM | HAR_ani | HAR_SHADE | HAR_NoMoRe | TAAM |
|---|---|---|---|---|---|
| mean overlap ($\bar{S}$ ) | 0.91 | 4.14 | 0.47 | 0.63 | 0.73 |
| H4 | 1.11 | 3.19 | 0.43 | 1.34 | 1.11 |
| H5 | 2.26 | 0.33 | 0.70 | 1.01 | 1.80 |
| H3 | 0.49 | 3.31 | 0.51 | 0.18 | 0.30 |
| H2 | 0.17 | 1.32 | 0.17 | 0.2 | 0.17 |
| H1 | 0.52 | 12.54 | 0.55 | 0.42 | 0.25 |

**Table S7** Statistical parameters of HAR and TAAM refinements of **1-cutoff**.

| Model | HAR_ani | HAR_SHADE | HAR_iso | TAAM_SHADE | TAAM_iso |
|---|---|---|---|---|---|
| $R[F^2 > 2\sigma(F^2)]$ | 0.011 | 0.012 | 0.015 | 0.016 | 0.020 |
| $wR(F^2)$ | 0.020 | 0.021 | 0.027 | 0.062 | 0.075 |
| # of reflections | 1766 | 1766 | 1766 | 1766 | 1766 |
| # of fit parameters | 226 | 160 | 160 | 160 | 171 |
| $chi^2$ | 2.71 | 3.01 | 4.91 | n/a | n/a |
| Goodness of fit | 1.65 | 1.73 | 2.22 | 2.51 | 3.03 |
| $\Delta\rho_{max}$ | 0.06 | 0.06 | 0.07 | 0.16 | 0.18 |
| $\Delta\rho_{min}$ | -0.06 | -0.06 | -0.09 | -0.14 | -0.20 |

**Table S8** Statistical parameters of HAR and TAAM refinements of **2-cutoff**.

| Model | HAR_ani | HAR_SHADE | HAR_iso | TAAM_SHADE | TAAM_iso |
|---|---|---|---|---|---|
| $R[F^2 > 2\sigma(F^2)]$ | 0.010 | 0.009 | 0.010 | 0.012 | 0.014 |
| $wR(F^2)$ | 0.013 | 0.011 | 0.013 | 0.030 | 0.036 |
| # of reflections | 1281 | 1281 | 1281 | 1281 | 1281 |
| # of fit parameters | 199 | 127 | 127 | 127 | 139 |
| chi$^2$ | 1.49 | 1.05 | 1.47 | n/a | n/a |
| Goodness of fit | 1.22 | 1.02 | 1.21 | 1.42 | 1.66 |
| $\Delta\rho_{max}$ | 0.07 | 0.07 | 0.08 | 0.09 | 0.10 |
| $\Delta\rho_{min}$ | -0.08 | -0.08 | -0.08 | -0.13 | -0.14 |

**Table S9** Statistical parameters of HAR and TAAM refinements of **3-cutoff**.

| Model | HAR_ani | HAR_SHADE | HAR_iso | TAAM_SHADE | TAAM_iso |
|---|---|---|---|---|---|
| $R[F^2 > 2\sigma(F^2)]$ | 0.013 | 0.016 | 0.017 | 0.018 | 0.018 |
| $wR(F^2)$ | 0.019 | 0.023 | 0.027 | 0.036 | 0.036 |
| # of reflections | 577 | 577 | 577 | 577 | 577 |
| # of fit parameters | 88 | 66 | 66 | 50 | 60 |
| chi$^2$ | 1.76 | 2.34 | 3.26 | n/a | n/a |
| Goodness of fit | 1.33 | 1.53 | 1.81 | 3.65 | 3.67 |
| $\Delta\rho_{max}$ | 0.04 | 0.05 | 0.06 | 0.21 | 0.21 |

| | | | | | |
|---|---|---|---|---|---|
| $\Delta\rho_{min}$ | -0.06 | -0.06 | -0.09 | -0.20 | -0.21 |

**Table S10** Similarity index (S) and mean similarity index ($\bar{S}$) calculated for **1-cutoff** in comparison with neutron data.

| | HAR aniso | HAR_SHADE | TAAM_SHADE |
|---|---|---|---|
| H9A | 0.86 | 0.40 | 0.37 |
| H9B | 2.38 | 1.58 | 1.71 |
| H1N | 8.23 | 1.31 | 1.48 |
| H8 | 1.32 | 0.36 | 0.46 |
| H10A | 0.71 | 0.87 | 0.81 |
| H10C | 1.89 | 1.01 | 1.14 |
| H10B | 0.44 | 0.97 | 1.01 |
| H2 | 4.01 | 0.81 | 0.81 |
| H3 | 4.03 | 0.28 | 0.49 |
| H7 | 3.03 | 0.61 | 0.67 |
| H4 | 1.21 | 0.44 | 0.64 |
| **mean overlap:** | **2.55** | **0.79** | **0.87** |

**Table S11** Similarity index (S) and mean similarity index ($\bar{S}$ ) calculated for **2-cutoff** in comparison with neutron data..

| | HAR_aniso | HAR_SHADE | TAAM_SHADE |
|---|---|---|---|
| H5A | 20.73 | 12.01 | 12.01 |
| H5B | 22.51 | 11.33 | 10.9 |
| H2 | 17.03 | 11.96 | 12.21 |
| H11 | 9.46 | 0.67 | 0.83 |
| H12 | 8.17 | 1.03 | 1.18 |
| H13 | 3.25 | 0.43 | 0.55 |
| H14 | 3.75 | 0.3 | 0.3 |
| H15 | 10.69 | 0.87 | 0.88 |
| H4 | 4.08 | 10.79 | 10.43 |
| H1B | 12.21 | 0.22 | 0.31 |
| H1A | 11.09 | 0.29 | 0.36 |
| H3 | 100 | 0.5 | 0.53 |
| **mean overlap** | **11.18/18.58** | **4.2** | **4.21** |

**Table S12** Similarity index (S) and mean similarity index ($\bar{S}$ ) calculated for **3-cutoff** in comparison with neutron data.

| | HAR_aniso | HAR_SHADE | TAAM_SHADE |
|---|---|---|---|
| H4 | 6.9 | 0.97 | 5.02 |
| H5 | 0.17 | 2.03 | 1.03 |
| H3 | 3.85 | 3.75 | 2.94 |
| H2 | 14.81 | 3.69 | 3.36 |
| H1 | 7.47 | 0.67 | 3.26 |
| **Mean overlap:** | **6.64** | **2.22** | **3.12** |

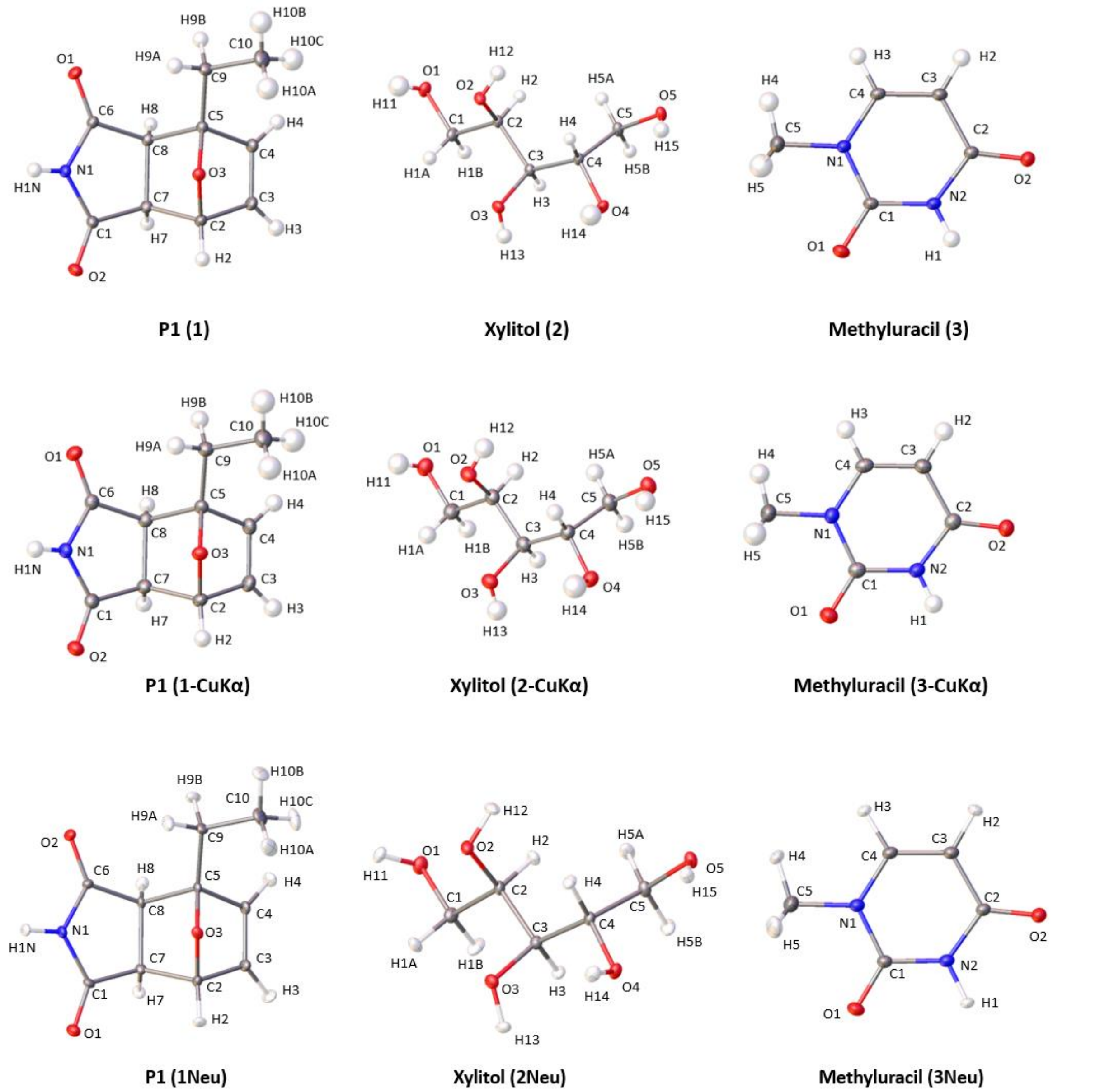


**Figure S1** An ORTEP view of the molecular structure obtained with the IAM refinement of the model compounds **1Cu, 2Cu, 3Mo** and **3Cu**. Anisotropic atomic displacement ellipsoids are drawn at the 50% probability level. Each data are available in the CSD: **1** [23], **2** [24], **2Neu** [25], **3Neu** [26], **1-CuKα**, **2-CuKα**, **3-CuKα**, **1Neu** [12].

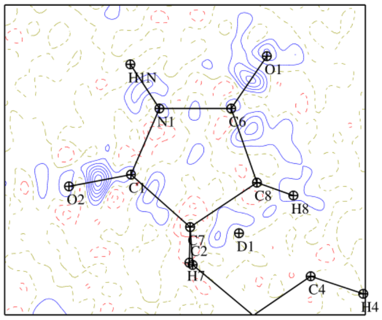


plane determined by N1 (centre), C6 (x axis) and C1 (y axis) atoms

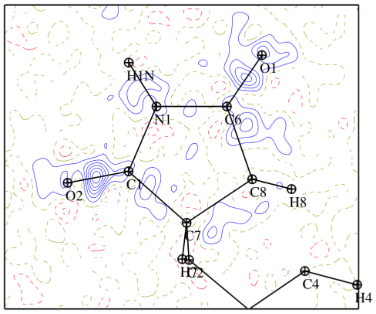


plane determined by N1 (centre), C6 (x axis) and C1 (y axis) atoms

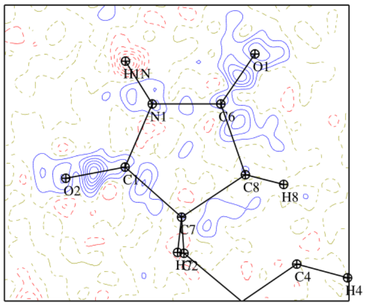


plane determined by N1 (centre), C6 (x axis) and C1 (y axis) atoms

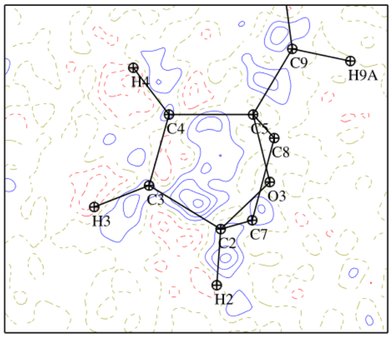


plane determined by C4 (centre), C5 (x axis) and C3 (y axis) atoms

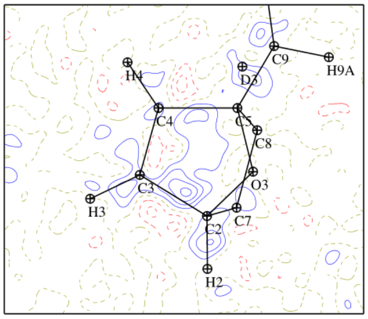


plane determined by C4 (centre), C5 (x axis) and C3 (y axis) atoms

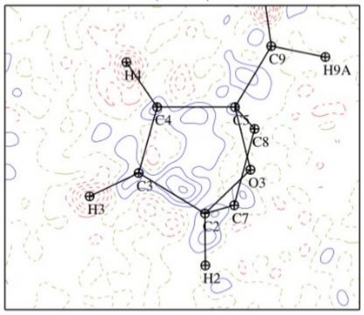


plane determined by C4 (centre), C5 (x axis) and C3 (y axis) atoms

MM

TAAM Mo Shade

TAAM Mo iso

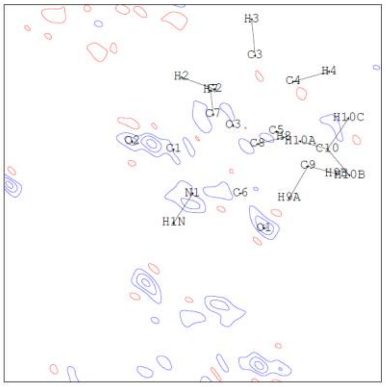


plane determined by N1 (centre), C6 (x axis) and C1 (y axis) atoms

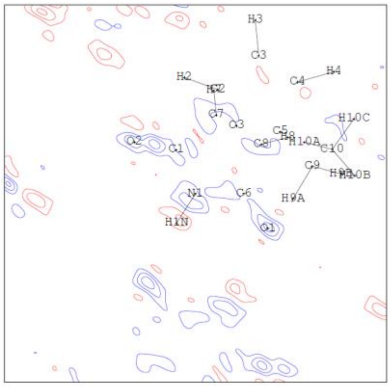


plane determined by N1 (centre), C6 (x axis) and C1 (y axis) atoms

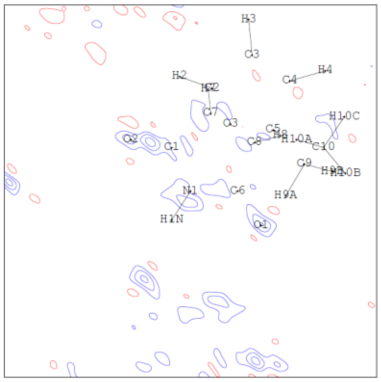


plane determined by N1 (centre), C6 (x axis) and C1 (y axis) atoms

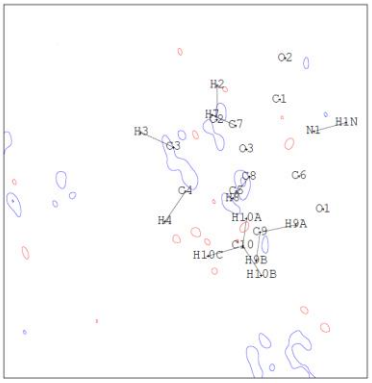

plane determined by C4 (centre), C5 (x axis) and C3 (y axis) atoms

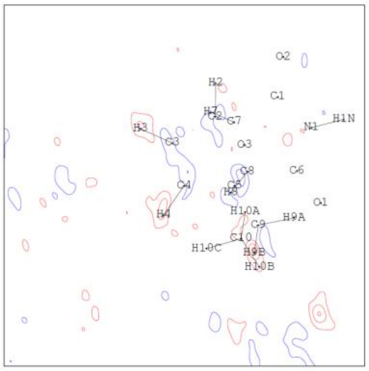

plane determined by C4 (centre), C5 (x axis) and C3 (y axis) atoms

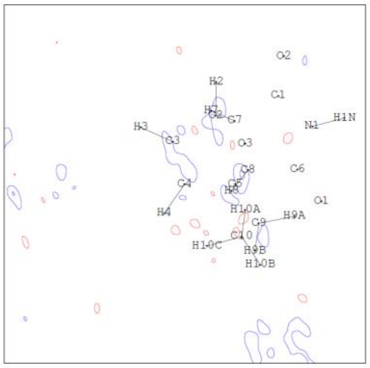

plane determined by C4 (centre), C5 (x axis) and C3 (y axis) atoms

HAR Mo aniso

HAR Mo iso

HAR Mo Shade

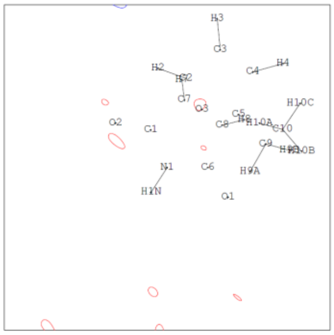

plane determined by N1 (centre), C6 (x axis) and C1 (y axis) atoms

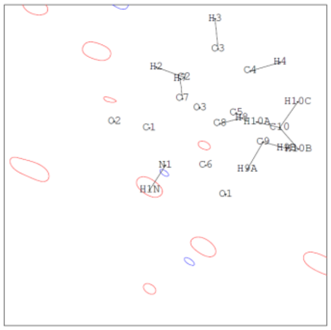

plane determined by N1 (centre), C6 (x axis) and C1 (y axis) atoms

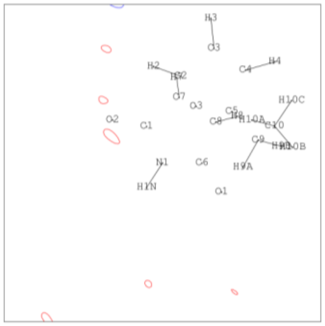

plane determined by N1 (centre), C6 (x axis) and C1 (y axis) atoms

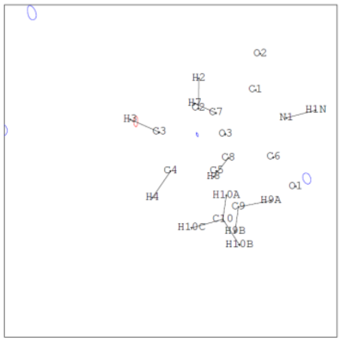

plane determined by C4 (centre), C5 (x axis) and C3 (y axis) atoms

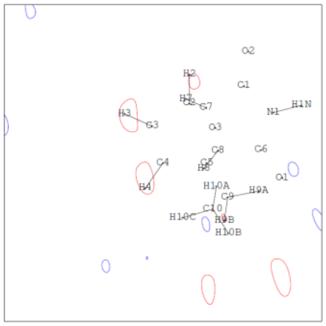

plane determined by C4 (centre), C5 (x axis) and C3 (y axis) atoms

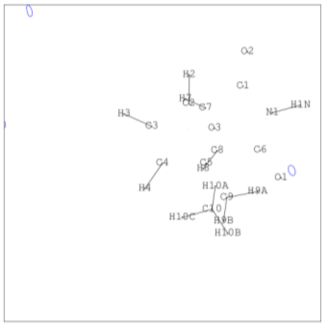

plane determined by C4 (centre), C5 (x axis) and C3 (y axis) atoms

HAR Mo aniso 0.81Å

HAR Mo iso 0.81Å

HAR Mo Shade 0.81Å

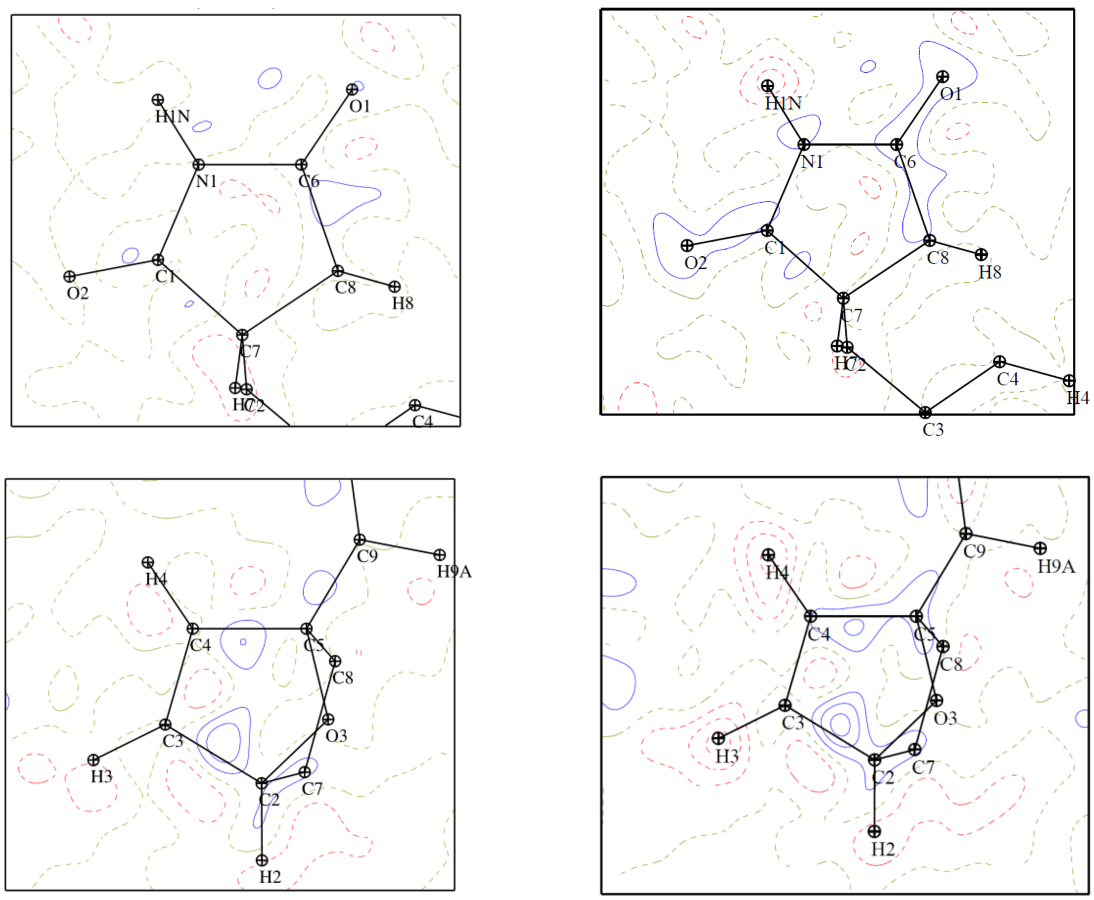


TAAM Shade 0.81Å

TAAM iso 0.81Å

**Figure S2** Residual density maps of **1** and **1-cutoff**. All maps are presented with contours levels with intervals ±0.05 eÅ$^{-3}$. Blue lines represent positive values and red lines the negative ones. Maps were prepared for planes determined by N1 (centre), C6 (x axis) and C1 (y axis) atoms or C4 (centre), C5 (x axis) and C3 (y axis) atoms. Details are given below the maps.

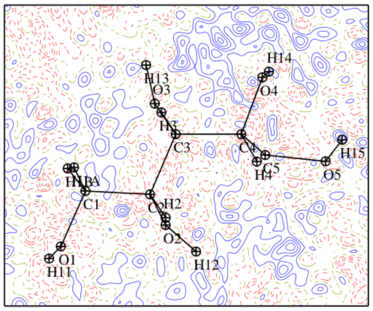


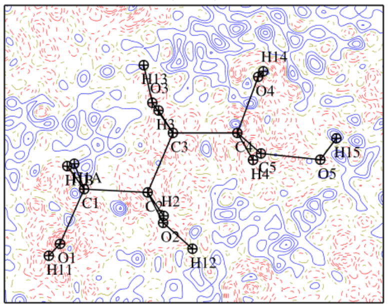


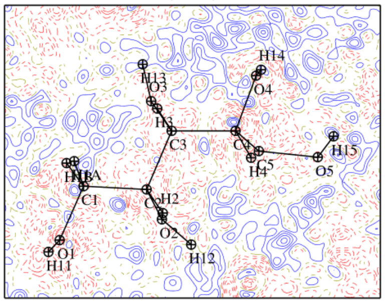


MM

TAAM Mo Shade

TAAM Mo iso

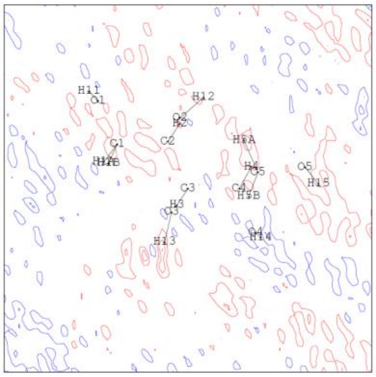

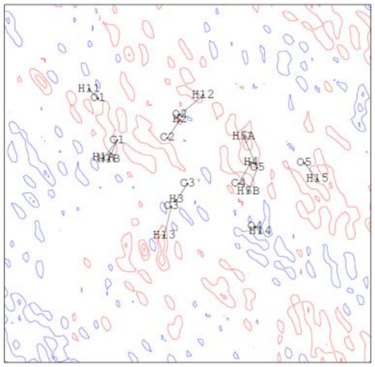

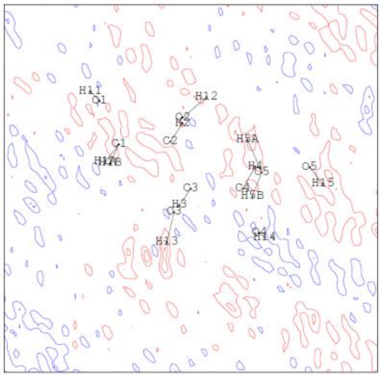

HAR Mo aniso

HAR Mo iso

HAR Mo

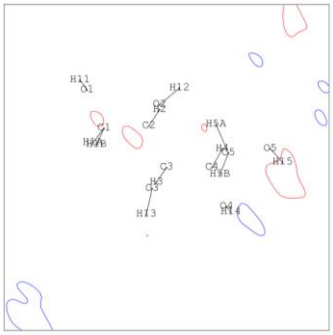

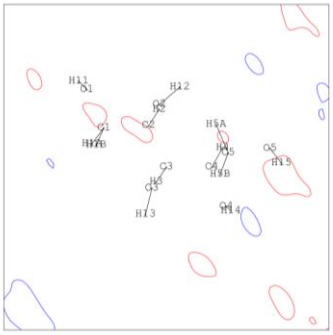

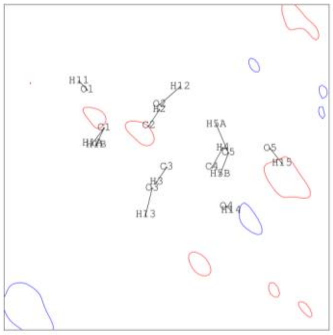

HAR Mo aniso 0.81Å

HAR Mo iso 0.81Å

HAR Mo Shade 0.81Å

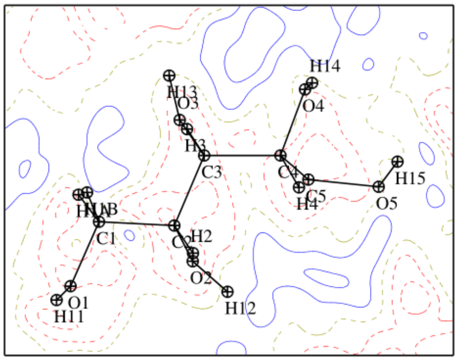


TAAM Shade 0.81Å

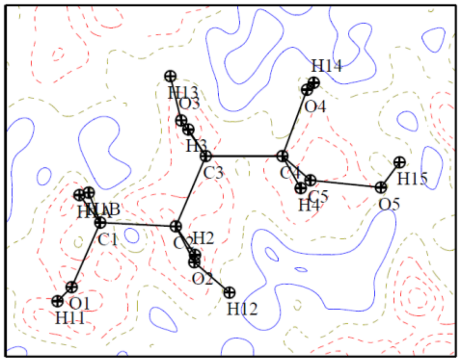


TAAM iso 0.81Å

**Figure S3** Residual density maps of **2** and **2-cutoff** (details are given below the maps). All maps are presented with contours levels with intervals ±0.05 eÅ$^{-3}$. Blue lines represent positive values and red lines the negative ones. Maps were prepared for planes determined by C3 (centre), C4 (x axis) and C2 (y axis) atoms.

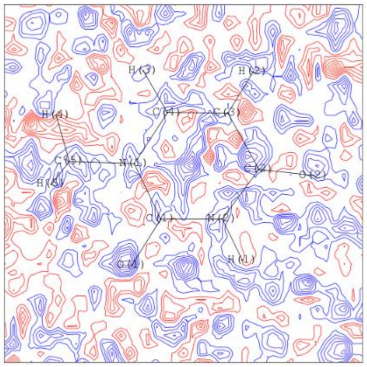

MM

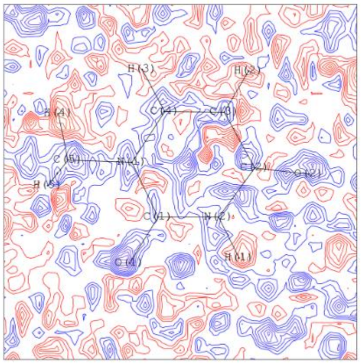

TAAM Mo Shade

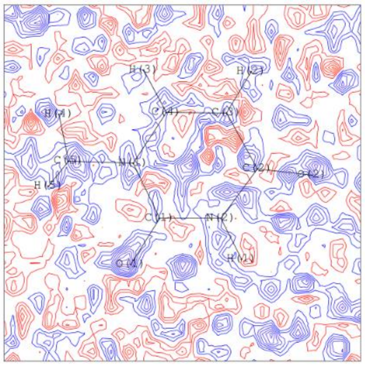

TAAM Mo iso

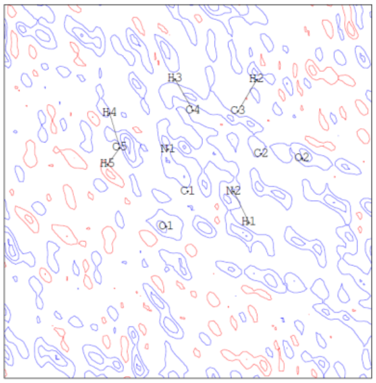


HAR Mo aniso

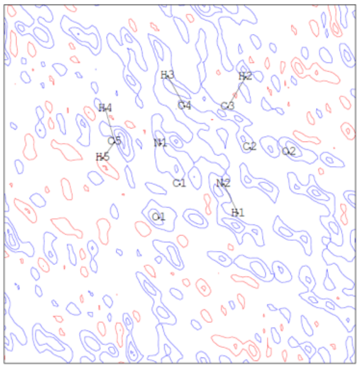


HAR Mo iso

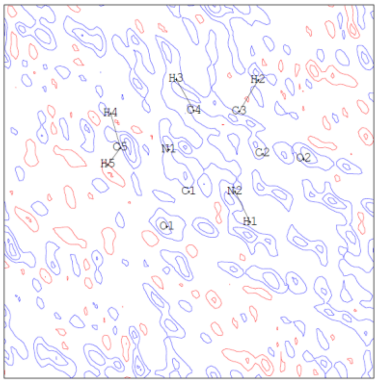


HAR Mo Shade

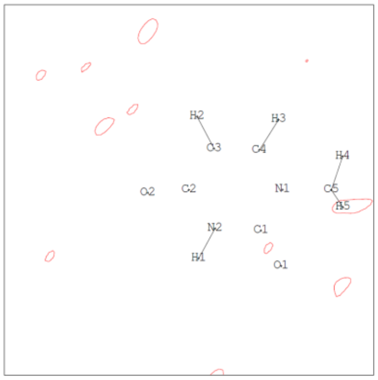


HAR Mo aniso 0.81Å

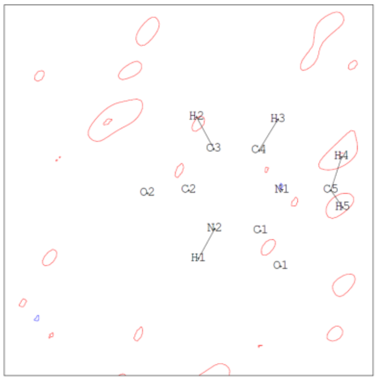


HAR Mo iso 0.81Å

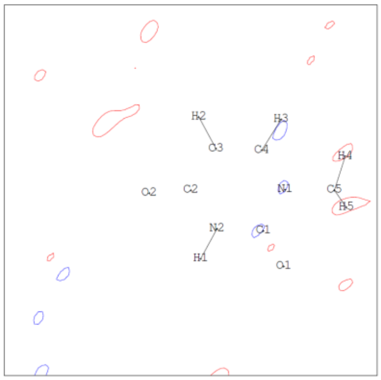


HAR Mo Shade 0.81Å

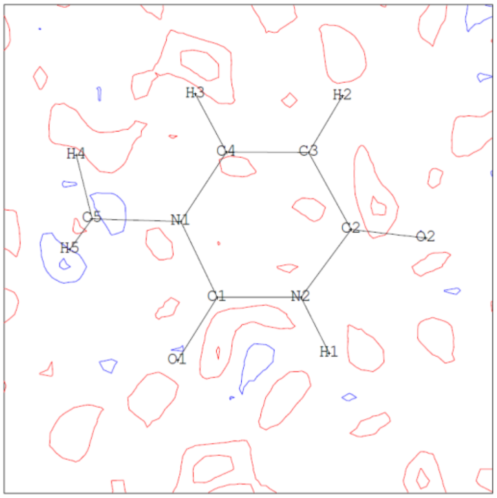


TAAM Shade 0.81Å

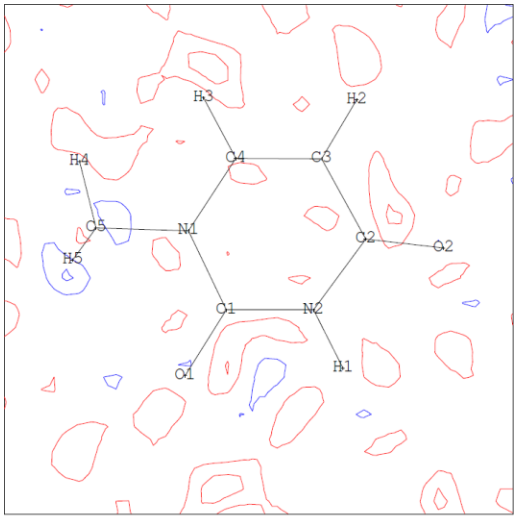


TAAM iso 0.81Å

**Figure S4** Residual density maps of **3** and **3-cutoff** (details are given below the maps). All maps are presented with contours levels with intervals ±0.05 eÅ$^{-3}$. Blue lines represent positive values and red lines the negative ones. Maps were prepared for planes determined by C1 (centre), N2 (x axis) and C4 (y axis) atoms.

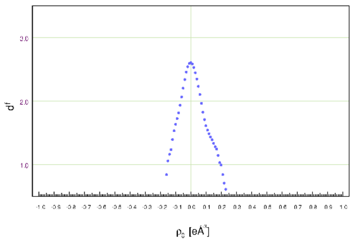

MM

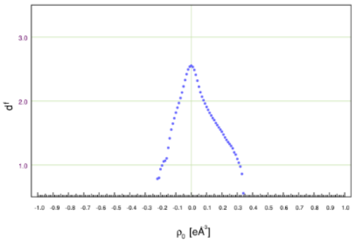

TAAM Mo Shade

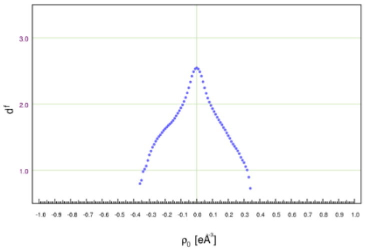

TAAM Mo iso

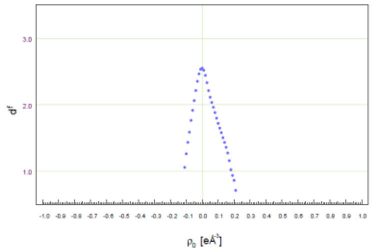

HAR Mo aniso

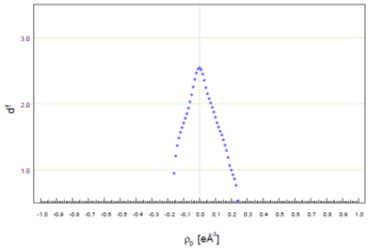

HAR Mo iso

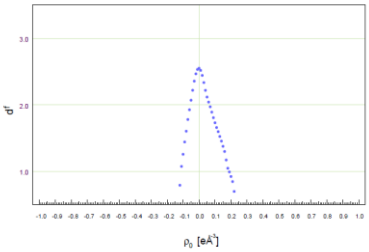

HAR Mo Shade

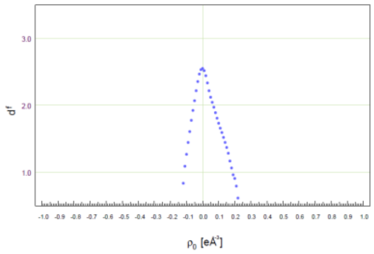

HAR_NoMoRe

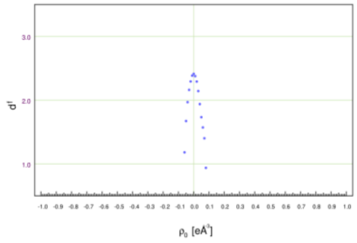

HAR Mo iso 0.81Å

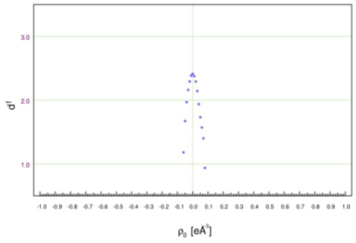

HAR Mo Shade 0.81Å

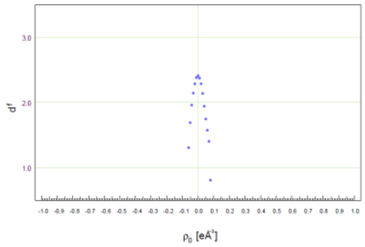

HAR Mo aniso 0.81Å

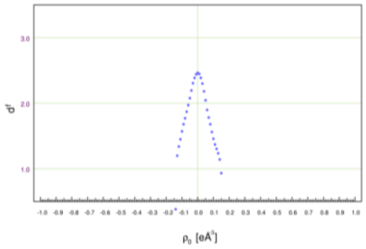

TAAM Mo Shade 0.81Å

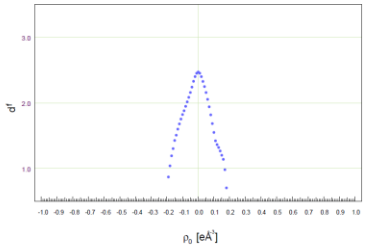

TAAM Mo iso 0.81Å

**Figure S5** Fractal plots of **1** and **1-cutoff**

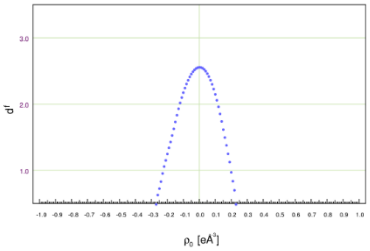

MM

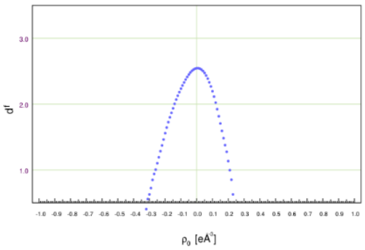

TAAM Mo Shade

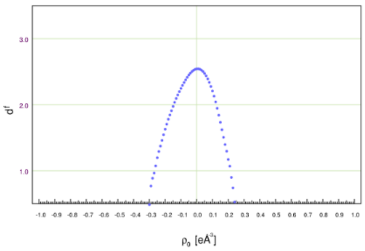

TAAM Mo iso

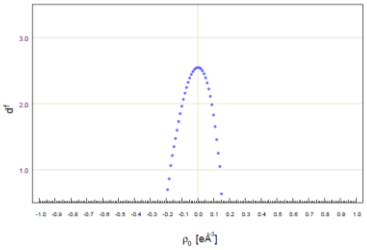

HAR Mo aniso

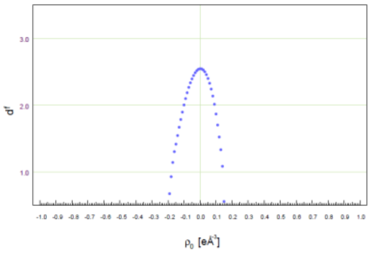

HAR Mo iso

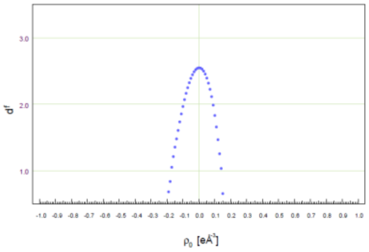

HAR Mo Shade

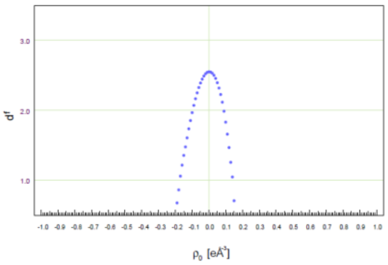

HAR_NoMoRe

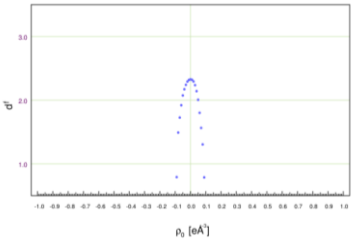

HAR Mo iso 0.81Å

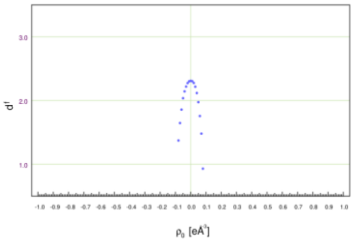

HAR Mo Shade 0.81Å

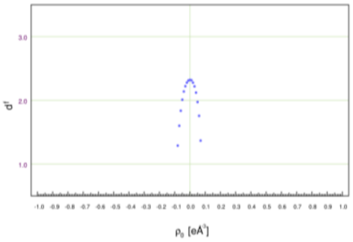

HAR Mo aniso 0.81Å

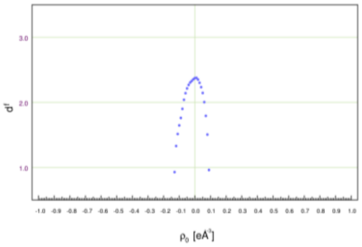

TAAM Shade 0.81Å

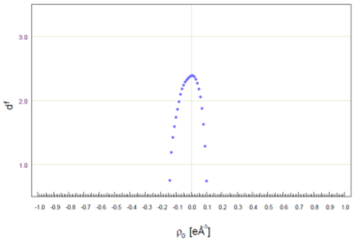

TAAM iso 0.81Å

**Figure S6** Fractal plots of **2** and **2-cutoff**

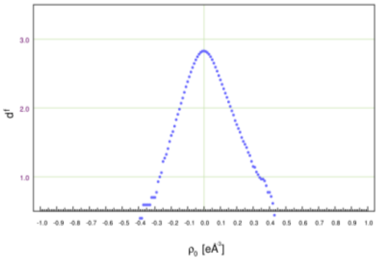

MM

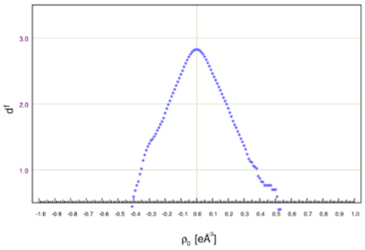

TAAM Mo Shade

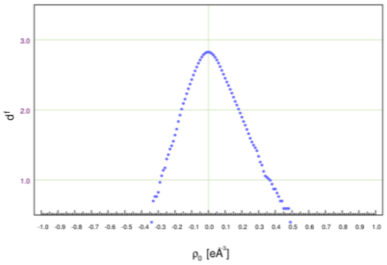

TAAM Mo iso

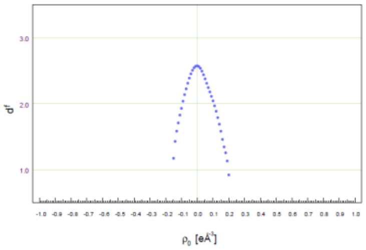

HAR Mo aniso

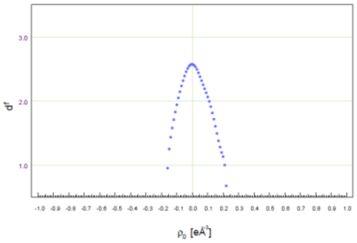

HAR Mo iso

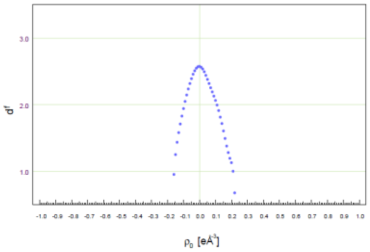

HAR Mo Shade

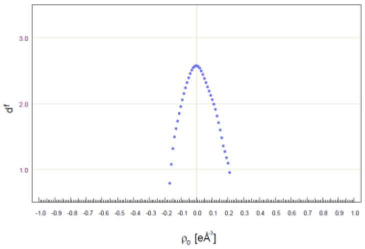

HAR Mo NoMoRe

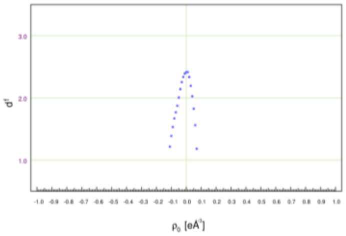

HAR Mo iso 0.81Å

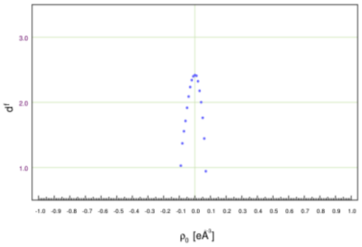

HAR Mo Shade 0.81Å

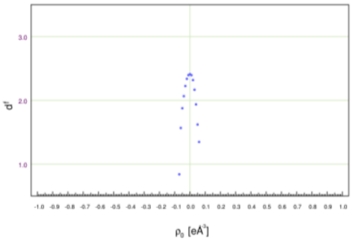

HAR Mo aniso 0.81Å

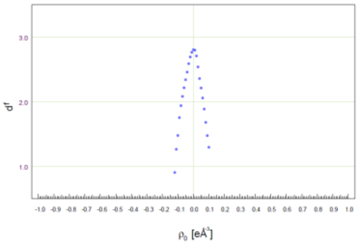

TAAM Shade 0.81Å

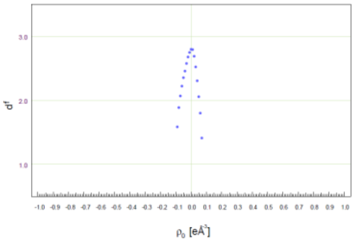

TAAM iso 0.81Å

**Figure S7** Fractal plots of **3** and **3-cutoff**

**Table S13** Summary of the $d^f$ maximum of the fractal dimension plots.

| | 1 | 2 | 3 |
|---|---|---|---|
| MM | 2.551 | 2.556 | 2.828 |
| TAAM_Mo_Shade | 2.551 | 2.545 | 2.827 |
| TAAM_Mo_iso | 2.546 | 2.542 | 2.827 |
| HAR_Mo_aniso | 2.546 | 2.550 | 2.577 |
| HAR_Mo_iso | 2.545 | 2.547 | 2.576 |
| HAR_Mo_Shade | 2.551 | 2.550 | 2.576 |
| HAR_NoMoRe | 2.551 | 2.550 | 2.576 |
| HAR_Mo_iso_0.81 | 2.412 | 2.327 | 2.413 |
| HAR_Mo_Shade_0.81 | 2.412 | 2.302 | 2.418 |
| HAR_Mo_aniso_0.81 | 2.412 | 2.321 | 2.410 |
| TAAM_Mo_Shade_0.81 | 2.464 | 2.373 | 2.780 |
| TAAM_Mo_iso_0.81 | 2.473 | 2.387 | 2.780 |

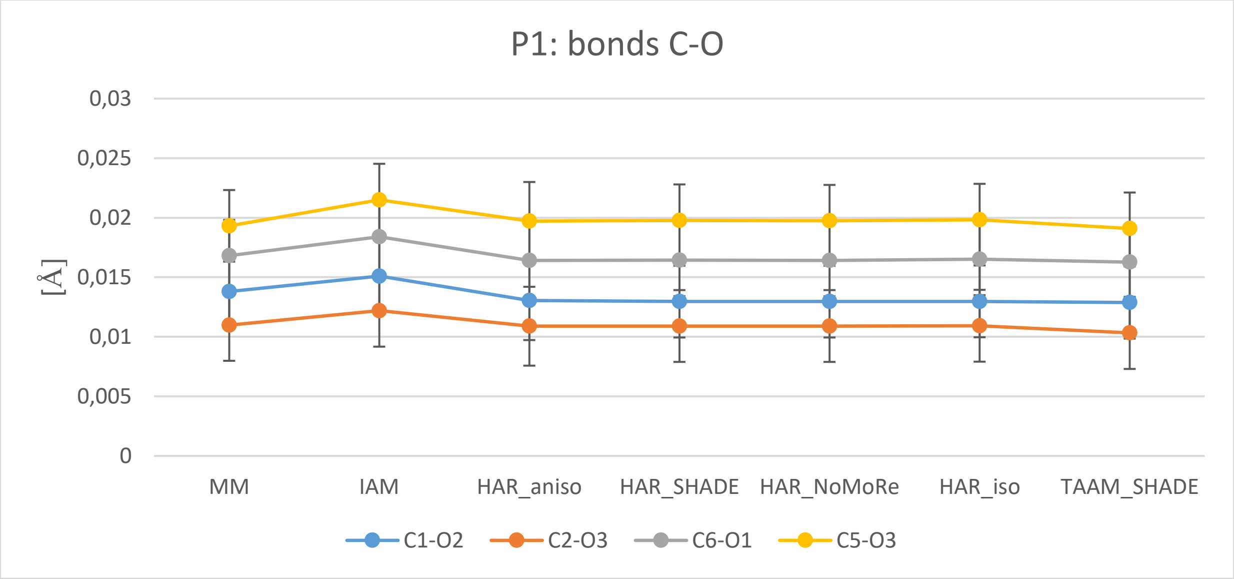

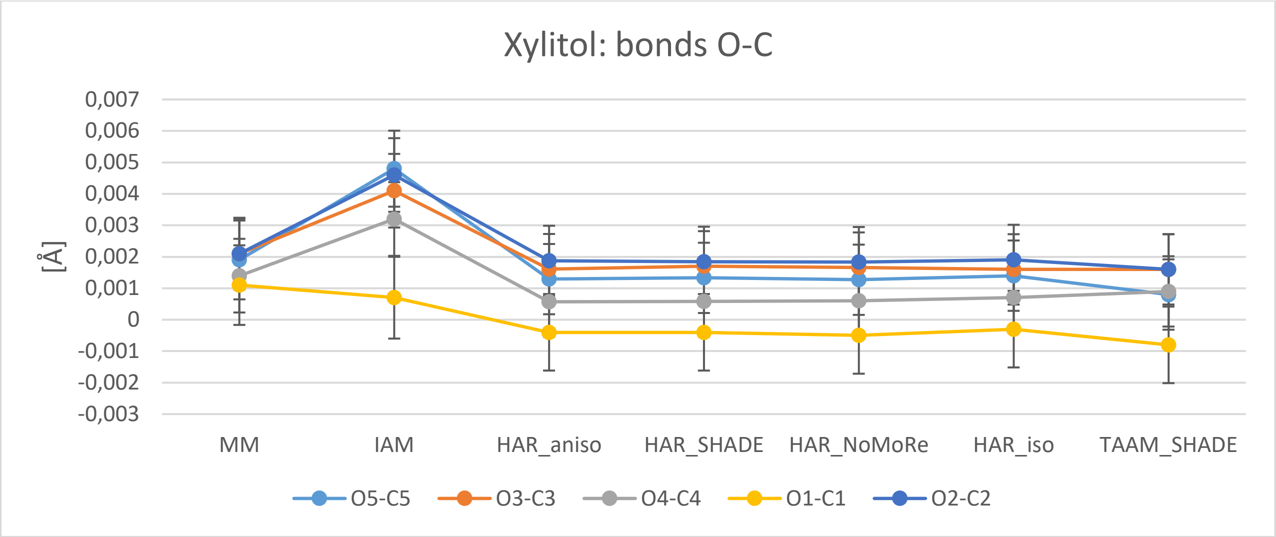


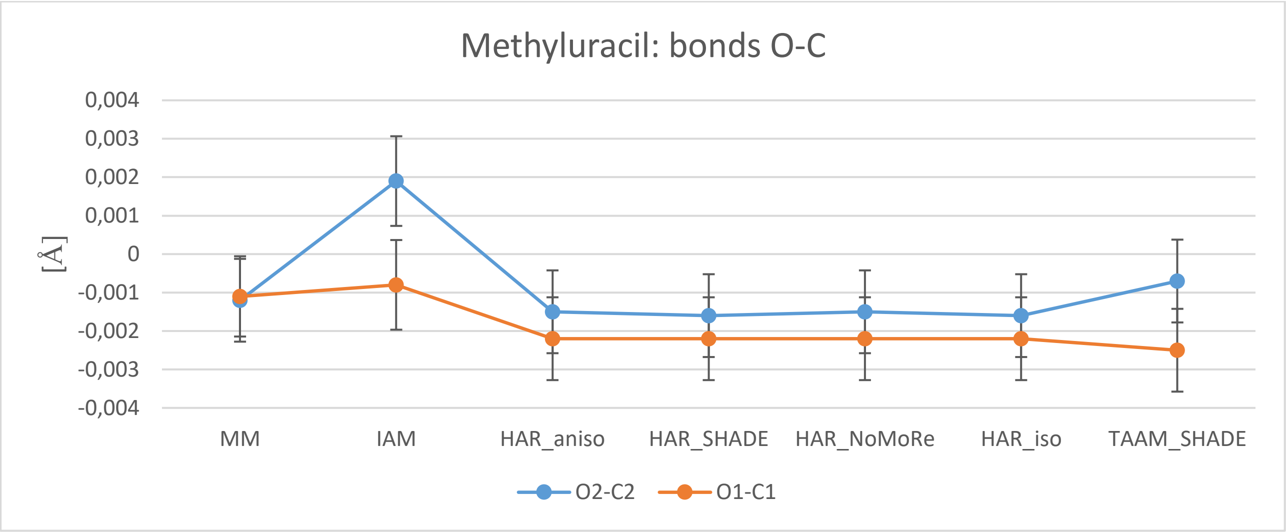


**Figure S8** Comparison of O-C bonds for IAM, MM, HAR and TAAM refinements of **1**, **2** and **3**. Values on plot represent difference between values obtained with analysed model and neutron data in angstroms. Lines on plot have no physical meaning, however help in visual analysis. For each plot estimated standard deviations were added.

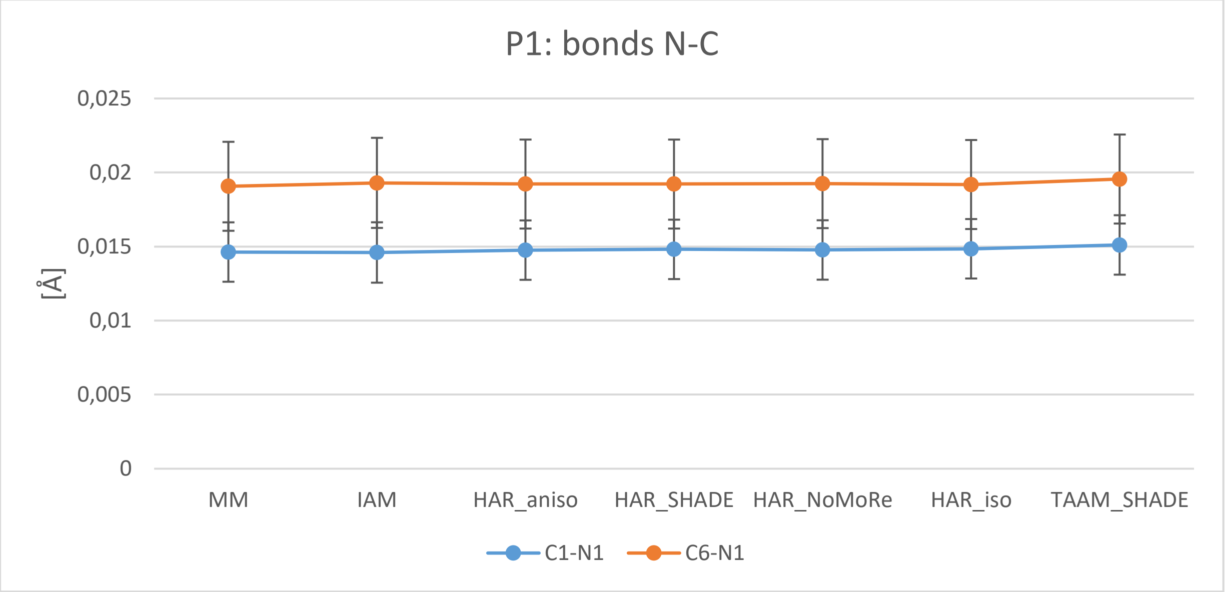


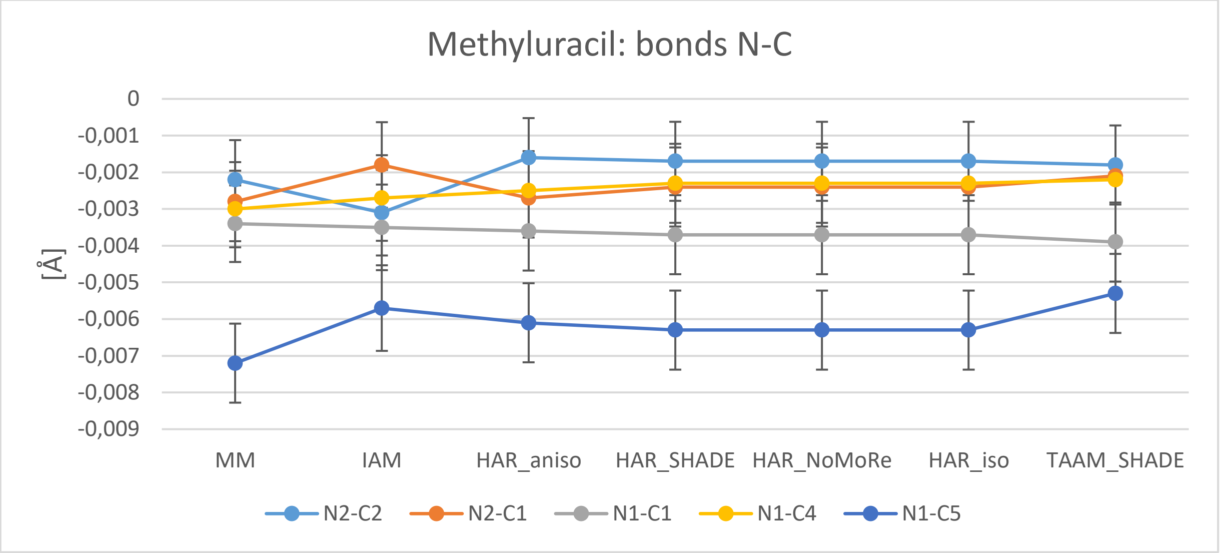


**Figure S9** Comparison of N-C bonds for IAM, MM, HAR and TAAM refinements of **1** and **3**. Values on plot represent difference between values obtained with analysed model and neutron data in angstroms. Lines on plot have no physical meaning, however help in visual analysis. For each plot estimated standard deviations were added.

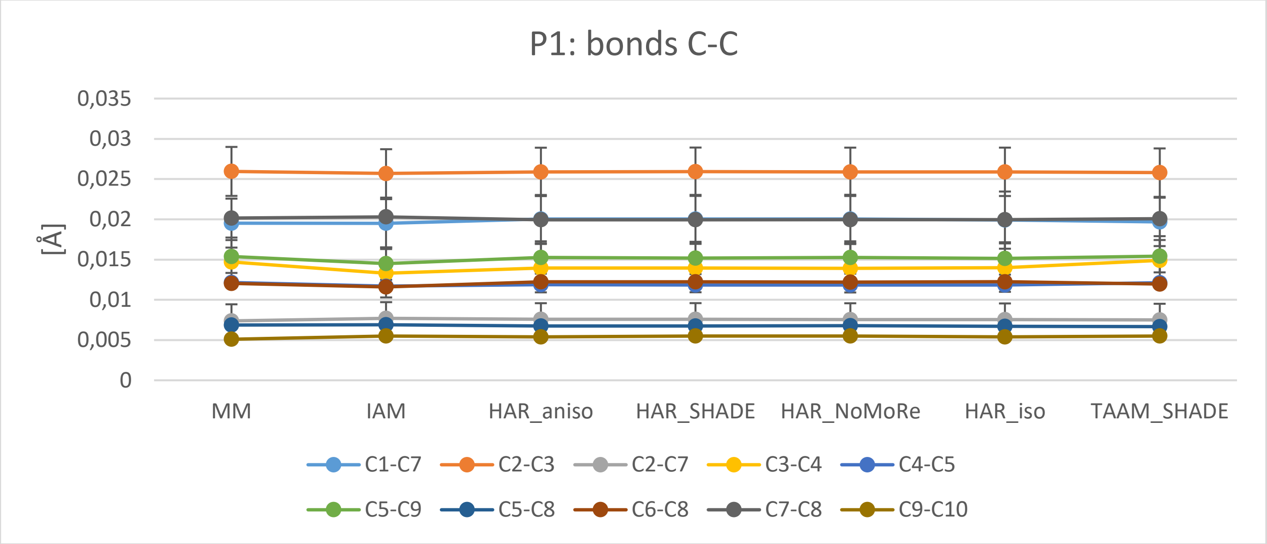
P1: bonds C-C
0,035
0,03
0,025
0,02
0,015
0,01
0,005
0
[Å]
MM
IAM
HAR_aniso
HAR_SHADE
HAR_NoMoRe
HAR_iso
TAAM_SHADE
C1-C7
C2-C3
C2-C7
C3-C4
C4-C5
C5-C9
C5-C8
C6-C8
C7-C8
C9-C10

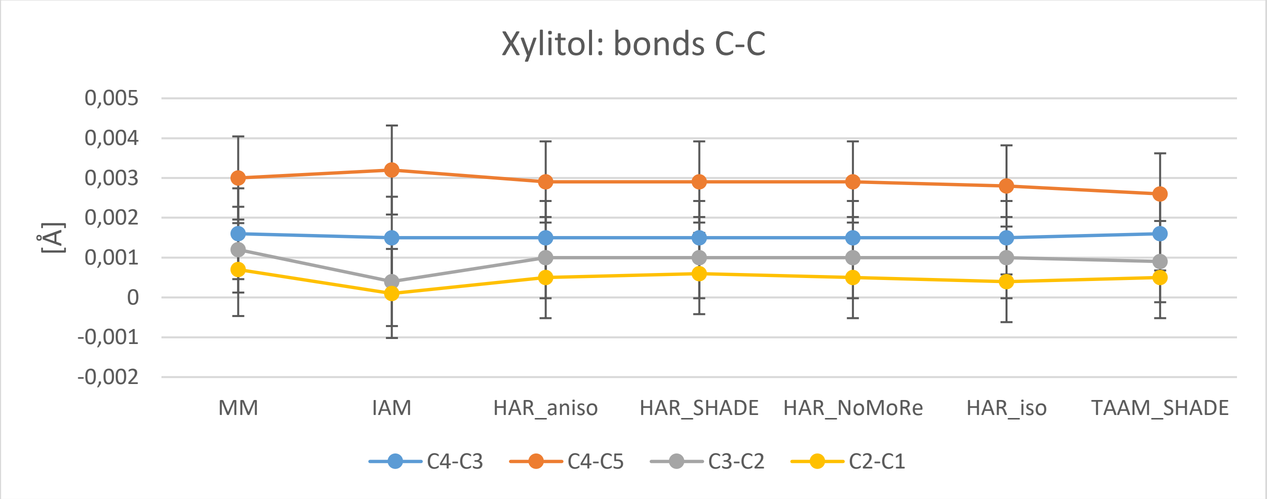
Xylitol: bonds C-C
0,005
0,004
0,003
0,002
0,001
0
-0,001
-0,002
[Å]
MM
IAM
HAR_aniso
HAR_SHADE
HAR_NoMoRe
HAR_iso
TAAM_SHADE
C4-C3
C4-C5
C3-C2
C2-C1

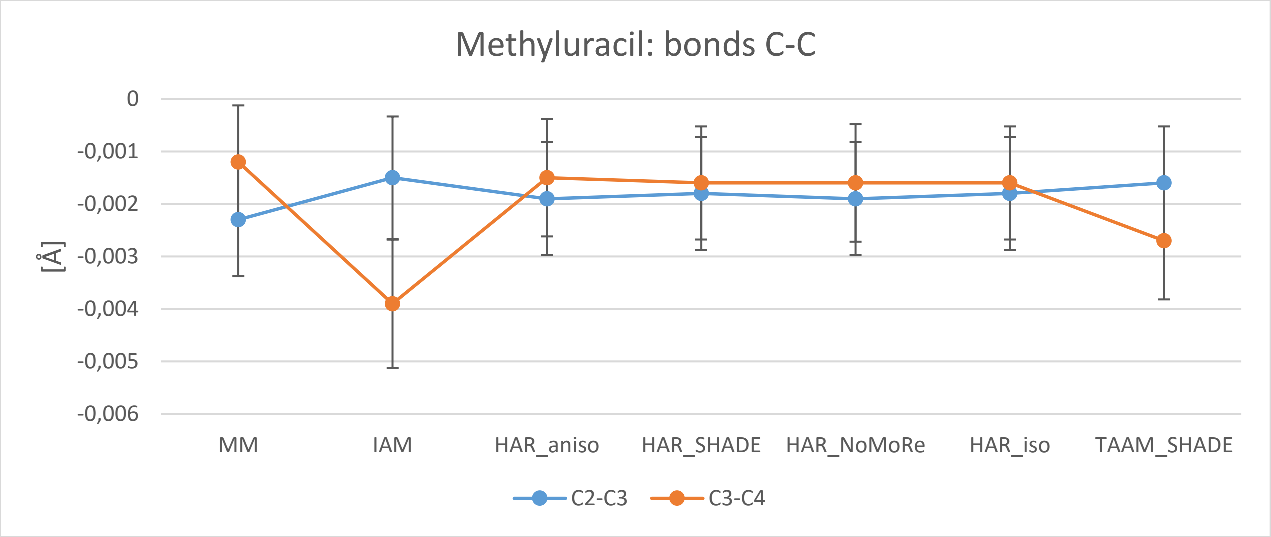
Methyluracil: bonds C-C
0
-0,001
-0,002
-0,003
-0,004
-0,005
-0,006
[Å]
MM
IAM
HAR_aniso
HAR_SHADE
HAR_NoMoRe
HAR_iso
TAAM_SHADE
C2-C3
C3-C4

**Figure S10** Comparison of C-C bonds for IAM, MM, HAR and TAAM refinements of **1**, **2** and **3**. Values on plot represent difference between values obtained with analysed model and neutron data in angstroms. Lines on plot have no physical meaning, however help in visual analysis. For each plot estimated standard deviations were added.

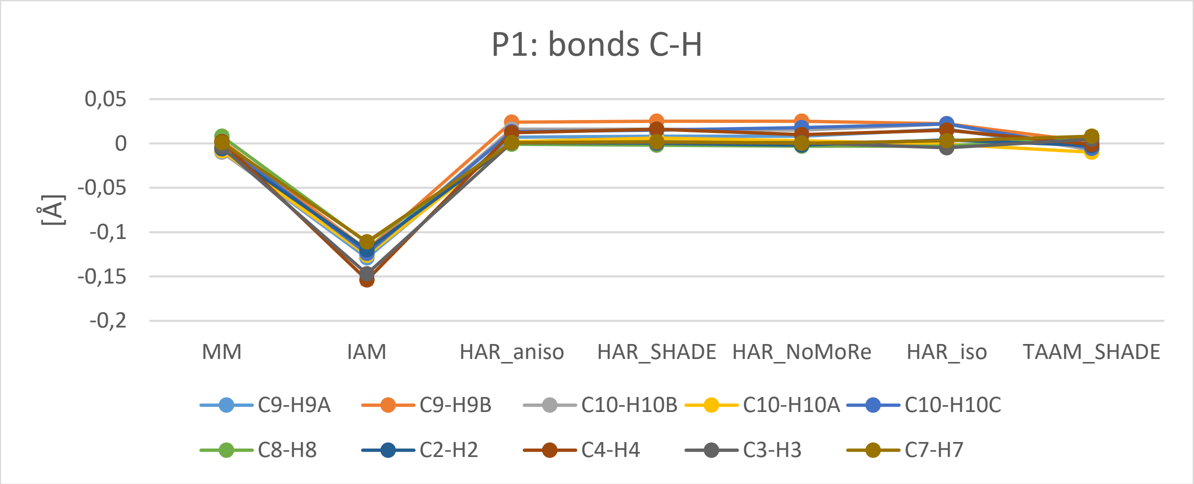


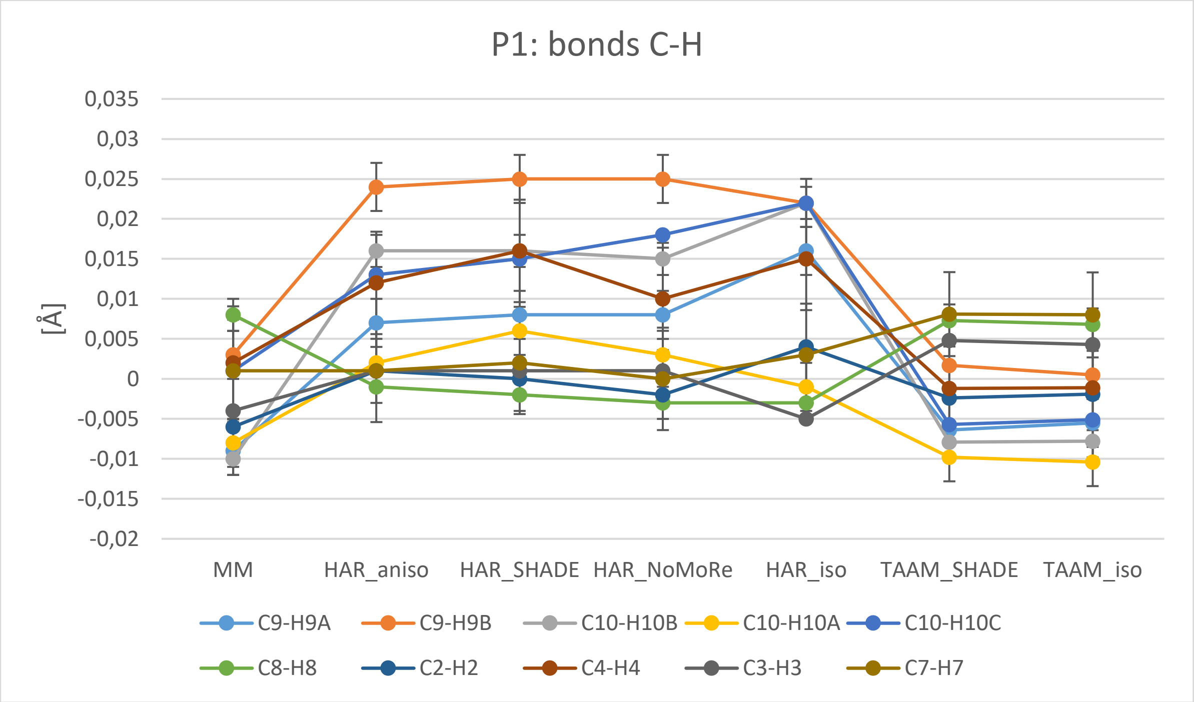

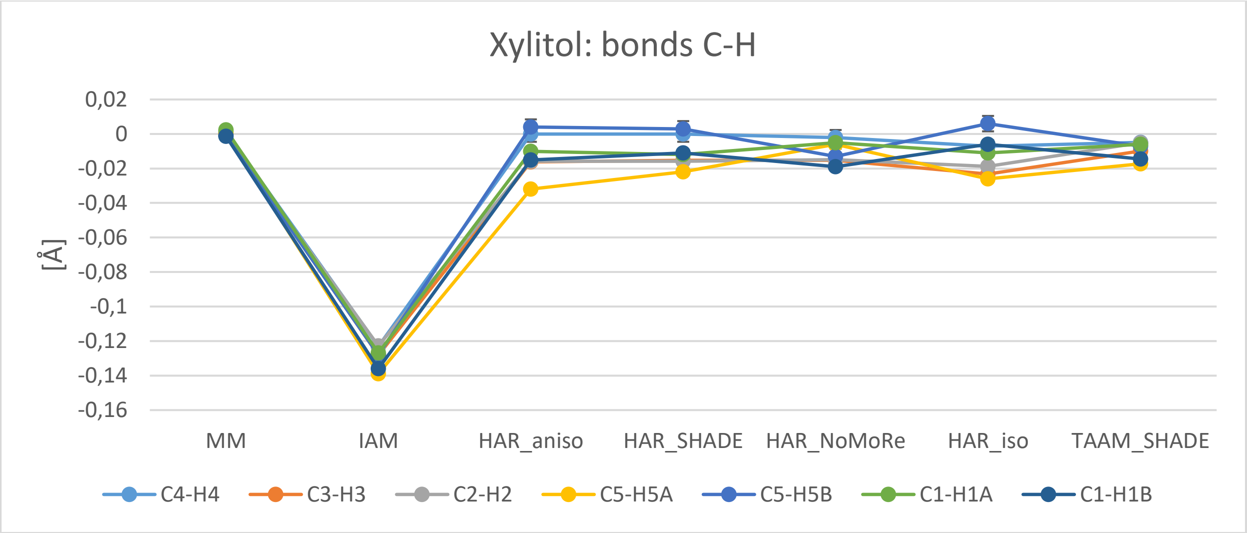
Xylitol: bonds C-H
0,02
0
-0,02
-0,04
-0,06
-0,08
-0,1
-0,12
-0,14
-0,16
[Å]
MM
IAM
HAR_aniso
HAR_SHADE
HAR_NoMoRe
HAR_iso
TAAM_SHADE
C4-H4
C3-H3
C2-H2
C5-H5A
C5-H5B
C1-H1A
C1-H1B

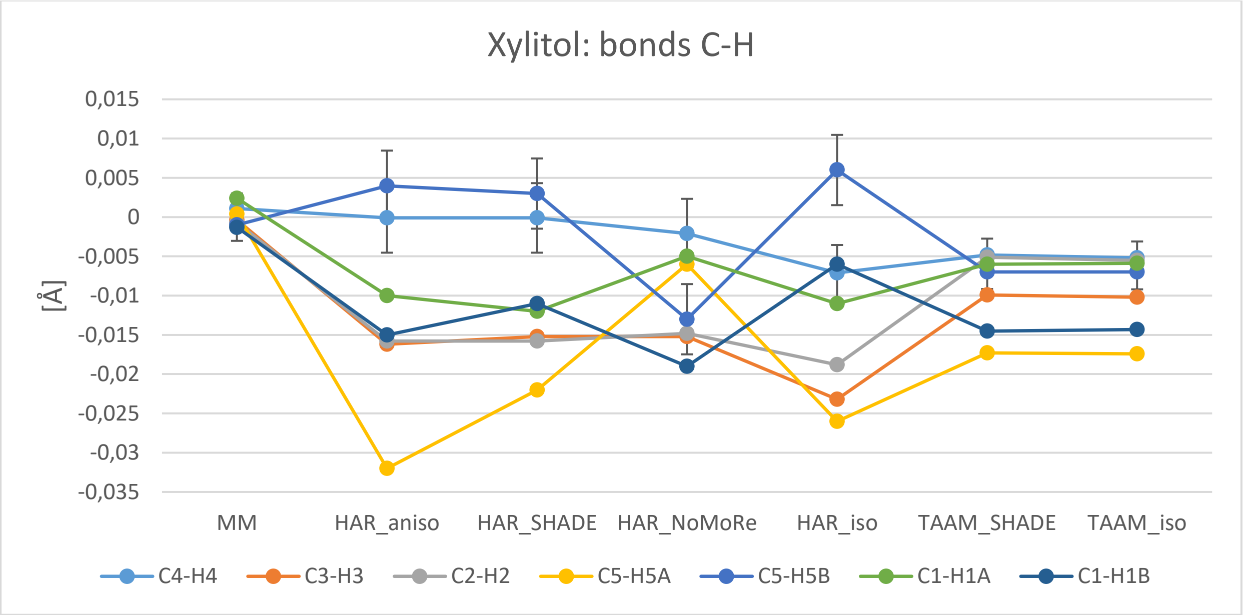
Xylitol: bonds C-H
0,015
0,01
0,005
0
-0,005
-0,01
-0,015
-0,02
-0,025
-0,03
-0,035
[Å]
MM
HAR_aniso
HAR_SHADE
HAR_NoMoRe
HAR_iso
TAAM_SHADE
TAAM_iso
C4-H4
C3-H3
C2-H2
C5-H5A
C5-H5B
C1-H1A
C1-H1B

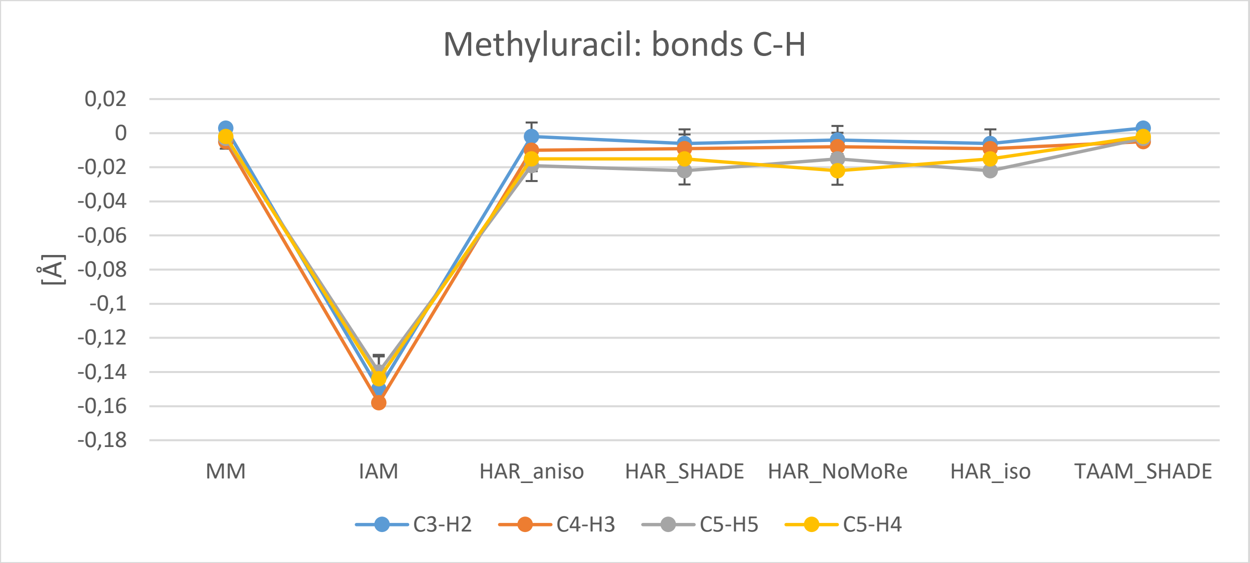


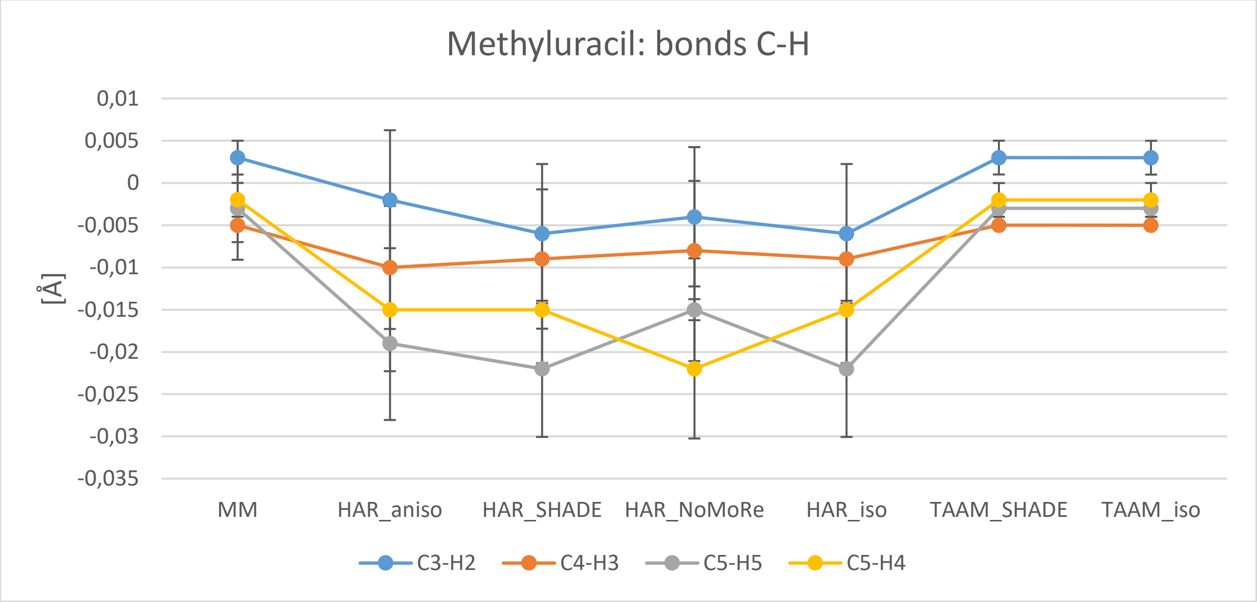


**Figure S11** Comparison of C-H bonds for IAM, MM, HAR and TAAM refinements of **1**, **2** and **3**. For clarity, additional plots without IAM are presented. Values on plot represent difference between values obtained with analysed model and neutron data in angstroms. Lines on plot have no physical meaning, however help in visual analysis. For each plot estimated standard deviations were added.

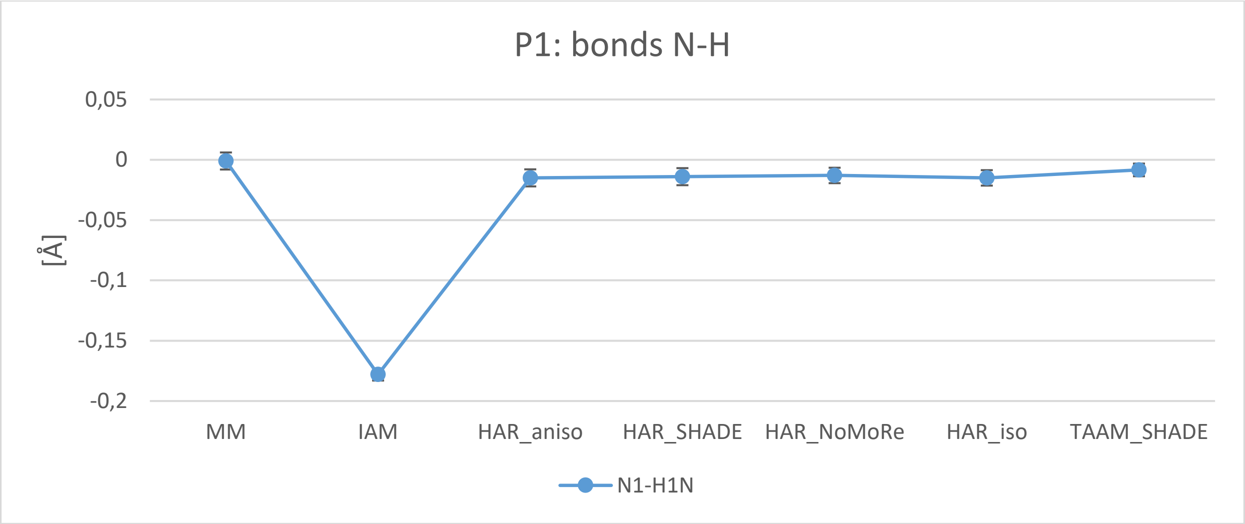
P1: bonds N-H
0,05
0
-0,05
-0,1
-0,15
-0,2
[Å]
MM
IAM
HAR_aniso
HAR_SHADE
HAR_NoMoRe
HAR_iso
TAAM_SHADE
N1-H1N

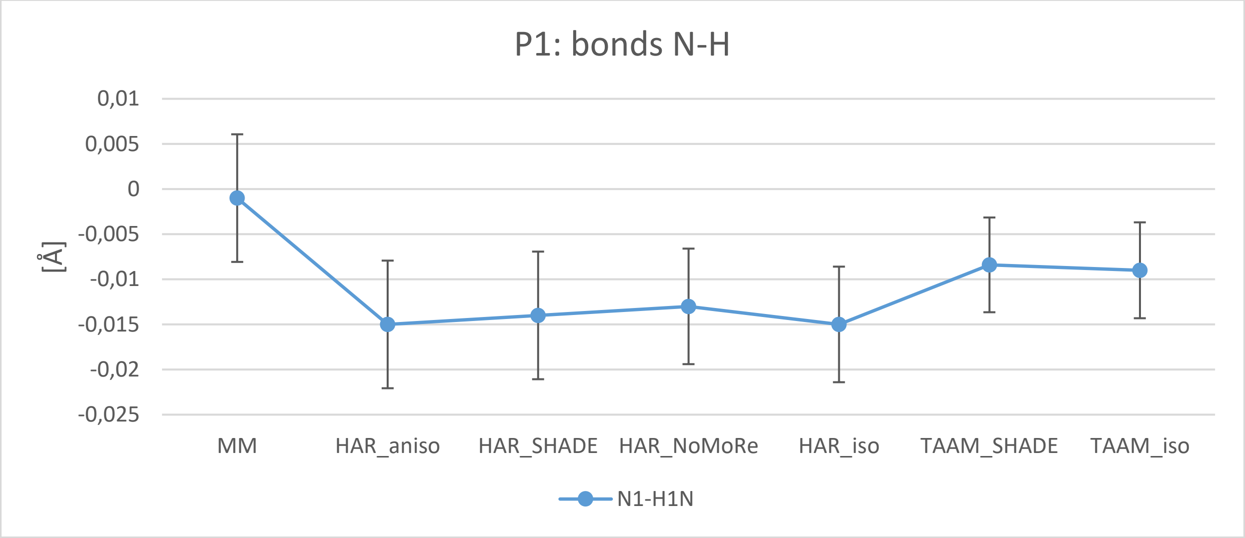
P1: bonds N-H
0,01
0,005
0
-0,005
-0,01
-0,015
-0,02
-0,025
[Å]
MM
HAR_aniso
HAR_SHADE
HAR_NoMoRe
HAR_iso
TAAM_SHADE
TAAM_iso
N1-H1N

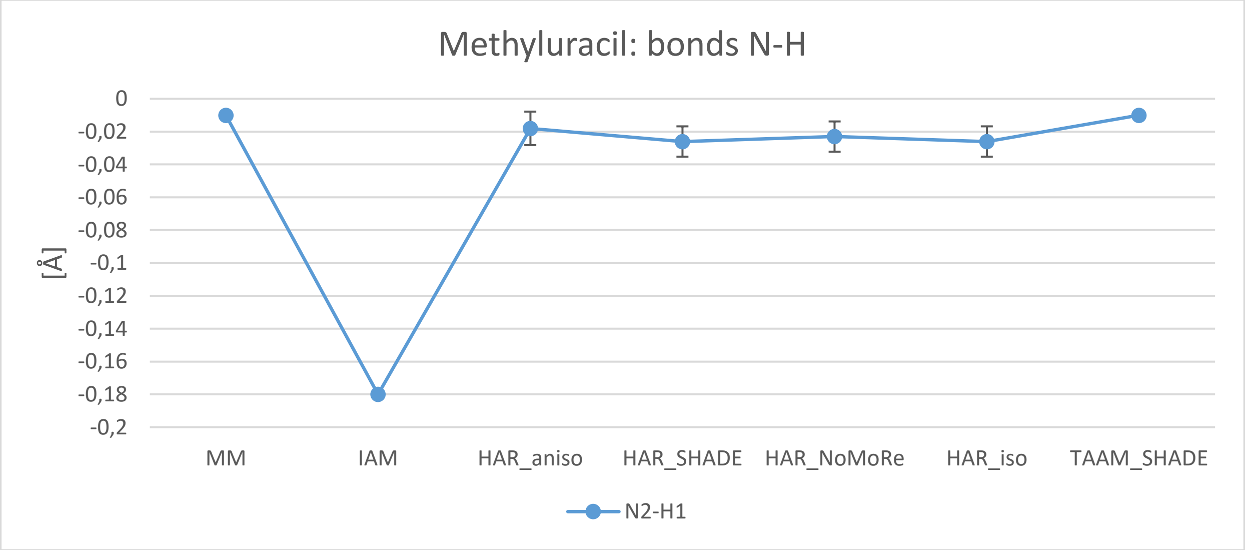
Methyluracil: bonds N-H
0
-0,02
-0,04
-0,06
-0,08
-0,1
-0,12
-0,14
-0,16
-0,18
-0,2
[Å]
MM
IAM
HAR_aniso
HAR_SHADE
HAR_NoMoRe
HAR_iso
TAAM_SHADE
N2-H1

v

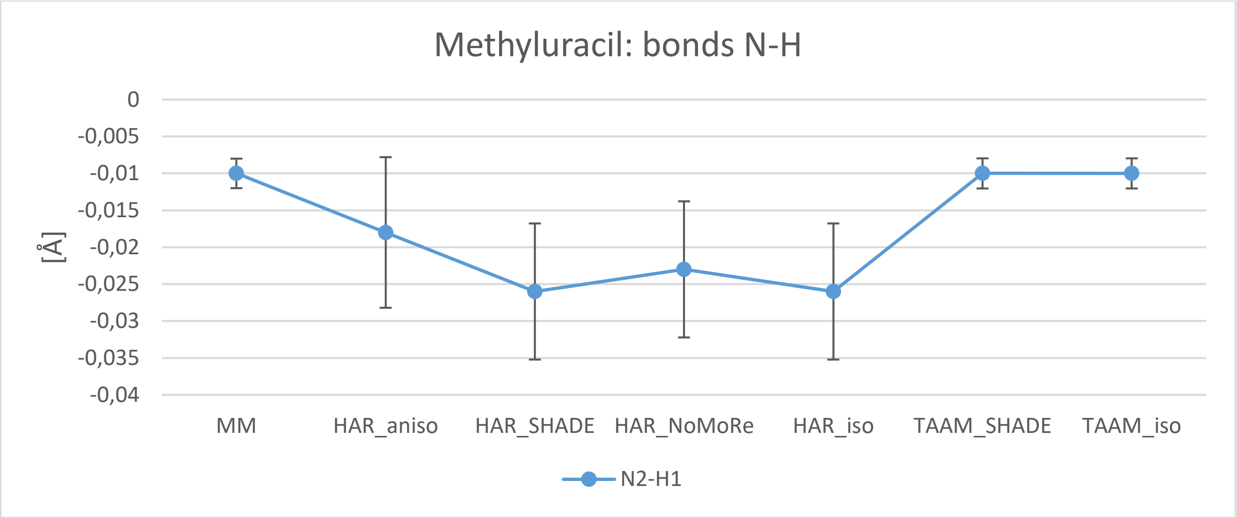


**Figure S12** Comparison of N-H bonds for IAM, MM, HAR and TAAM refinements of **1** and **3**. Values on plot represent difference between values obtained with analysed model and neutron data in angstroms. For clarity, additional plots without IAM are presented. Lines on plot have no physical meaning, however help in visual analysis. For each plot estimated standard deviations were added.

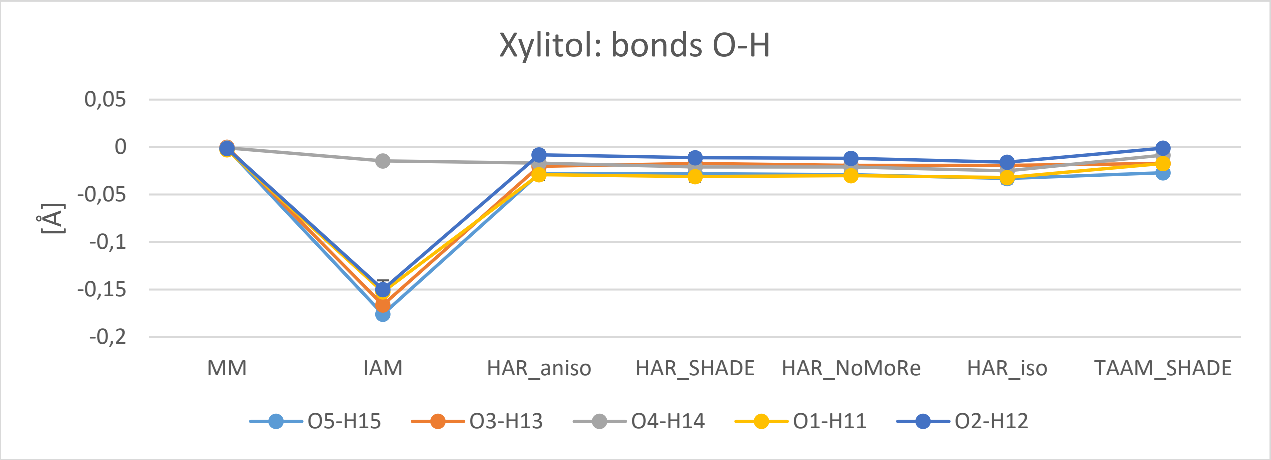

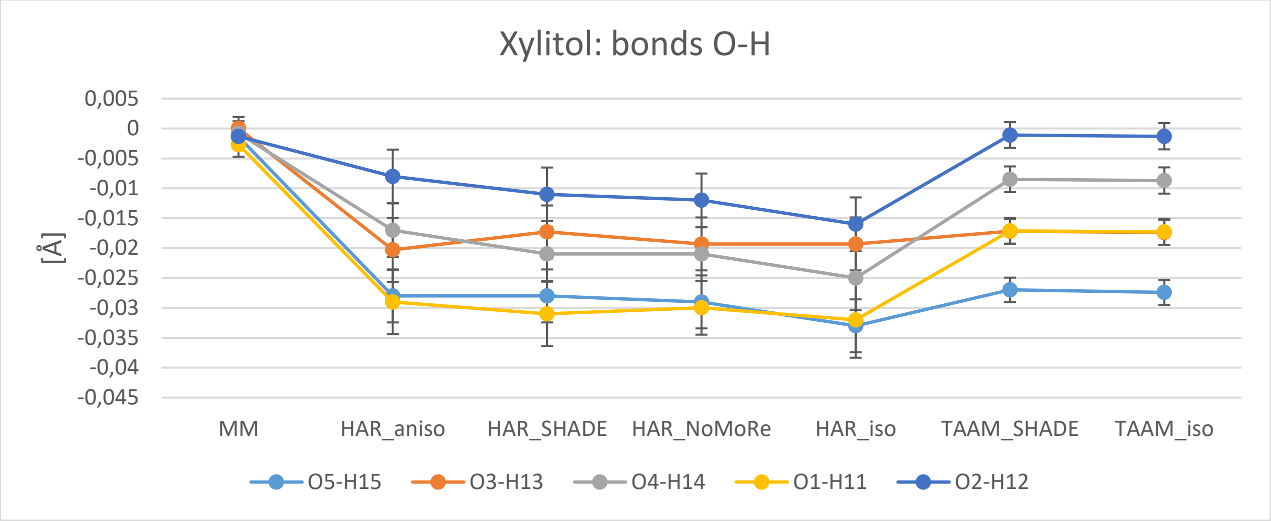


**Figure S13** Comparison of O-H bonds for IAM, MM, HAR and TAAM refinements of **2**. For clarity, additional plot without IAM are presented. Values on plot represent difference between values obtained with analysed model and neutron data in angstroms. Lines on plot have no physical meaning, however help in visual analysis. For each plot estimated standard deviations were added.

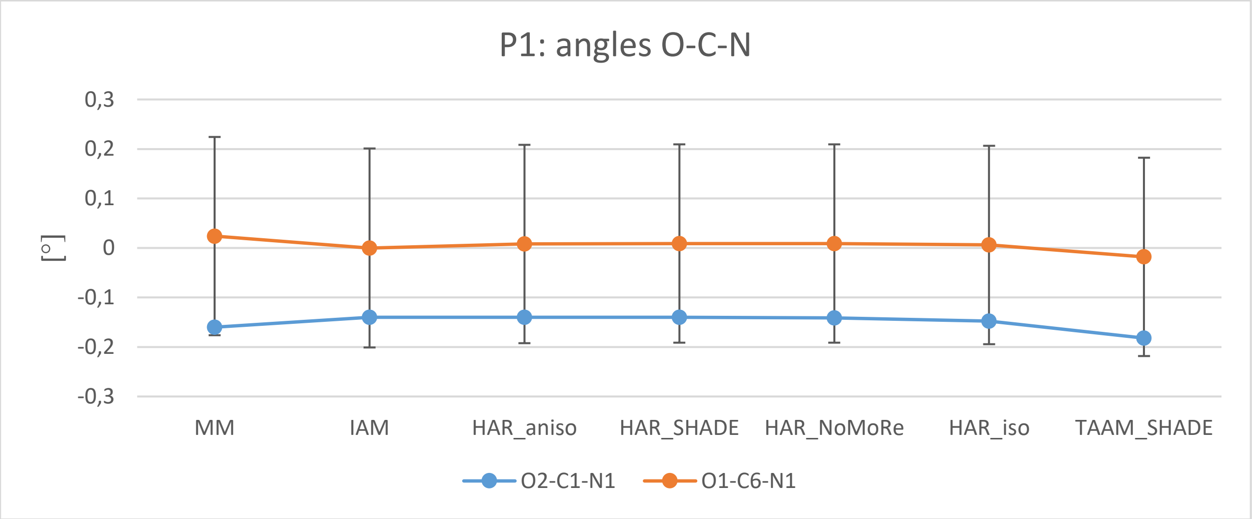

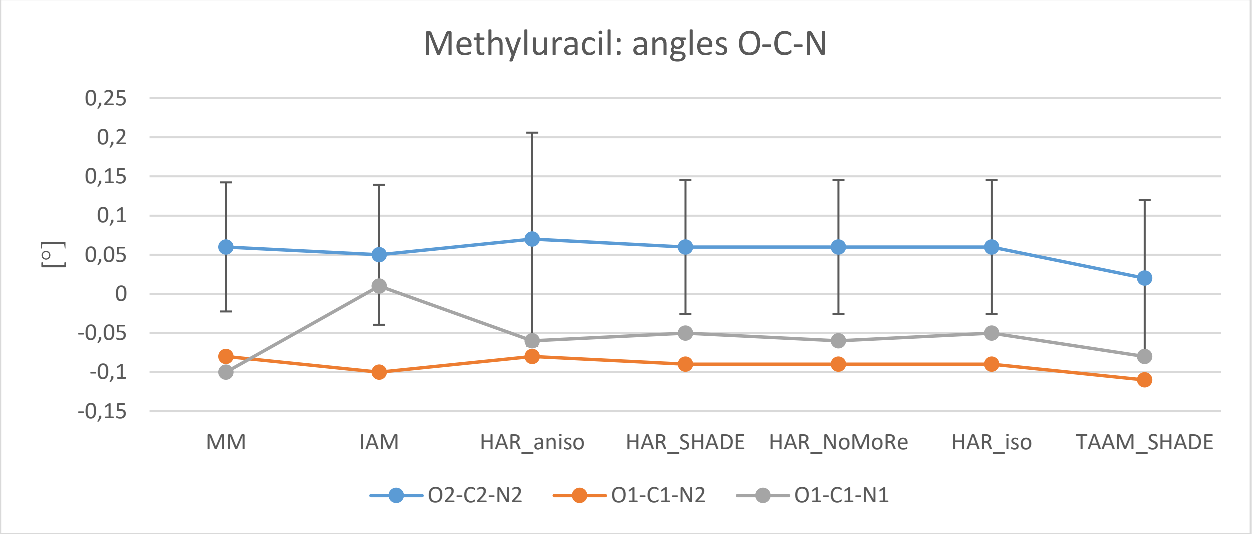


**Figure S14** Comparison of O-C-N angles for MM, IAM, HAR and TAAM refinements of **1** and **3**. Values on plot represent difference between values obtained with analysed model and neutron data in degrees. Lines on plot have no physical meaning, however help in visual analysis. For clarity standard deviations were added only for O1-C6-N1 (for P1) and O2-C2-N2 angles (for methyluracil).

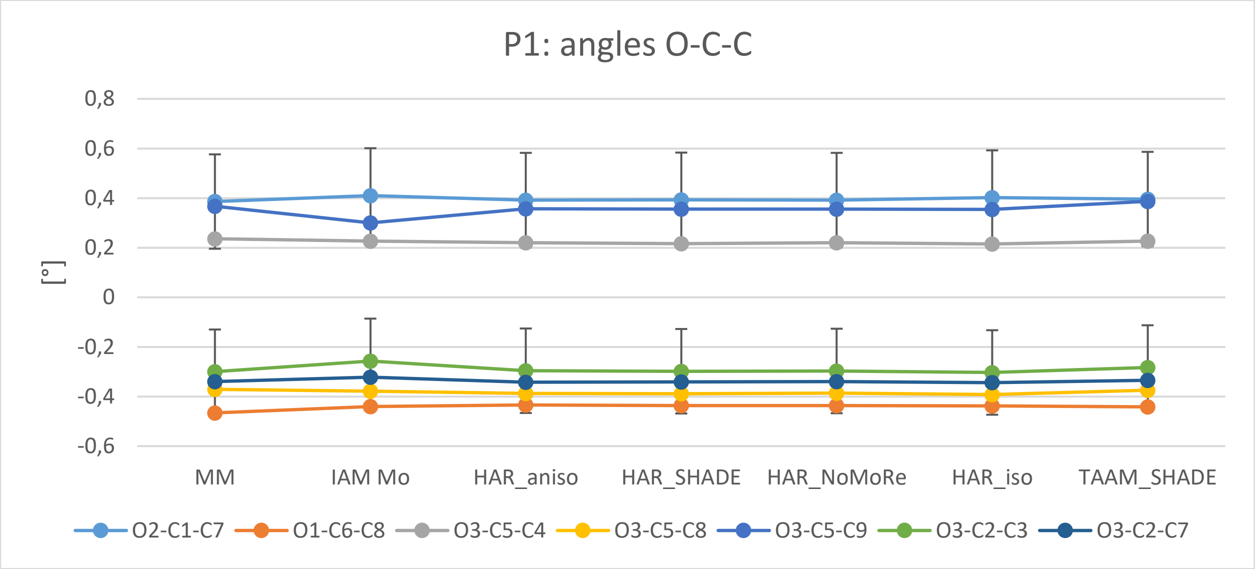

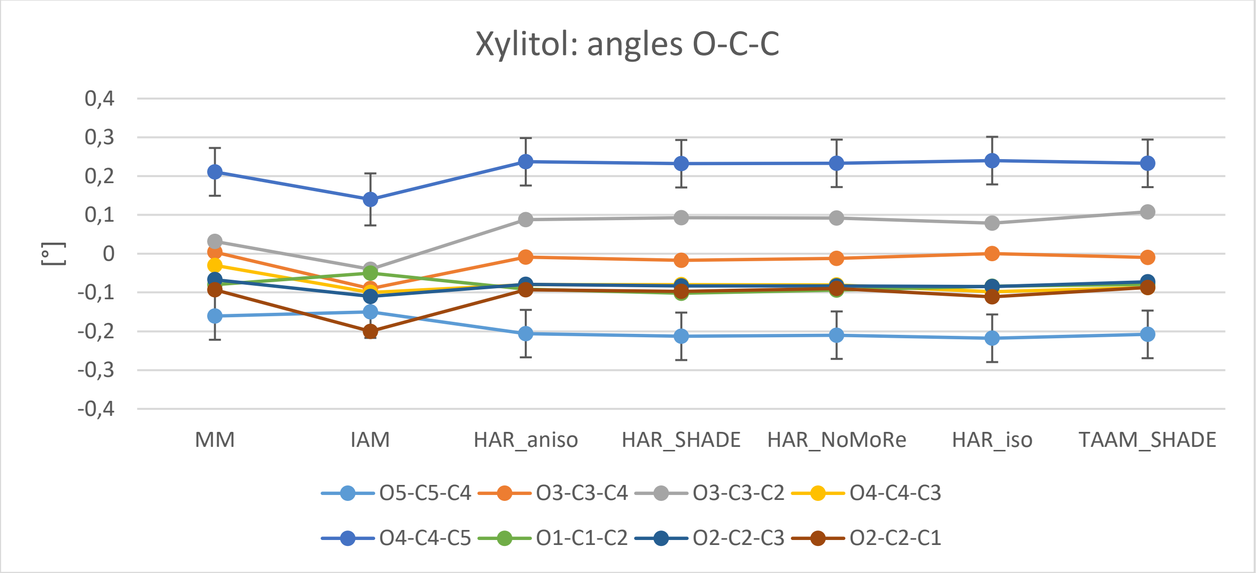


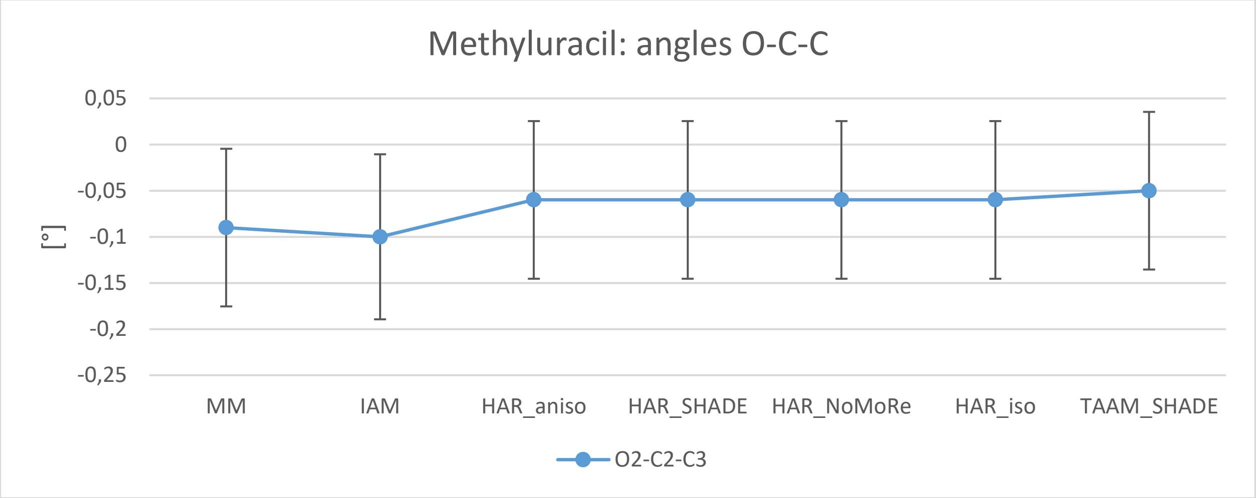


**Figure S15** Comparison of O-C-C angles for MM, IAM, HAR and TAAM refinements of **1**, **2** and **3**. Values on plot represent difference between values obtained with analysed model and neutron data in degrees. Lines on plot have no physical meaning, however help in visual analysis. For clarity estimated standard deviations for **1** were added only for O2-C1-C7 and O3-C2-C3 angles, whereas for **2** only for O5-C5-C4 and O4-C4-C5 angles.

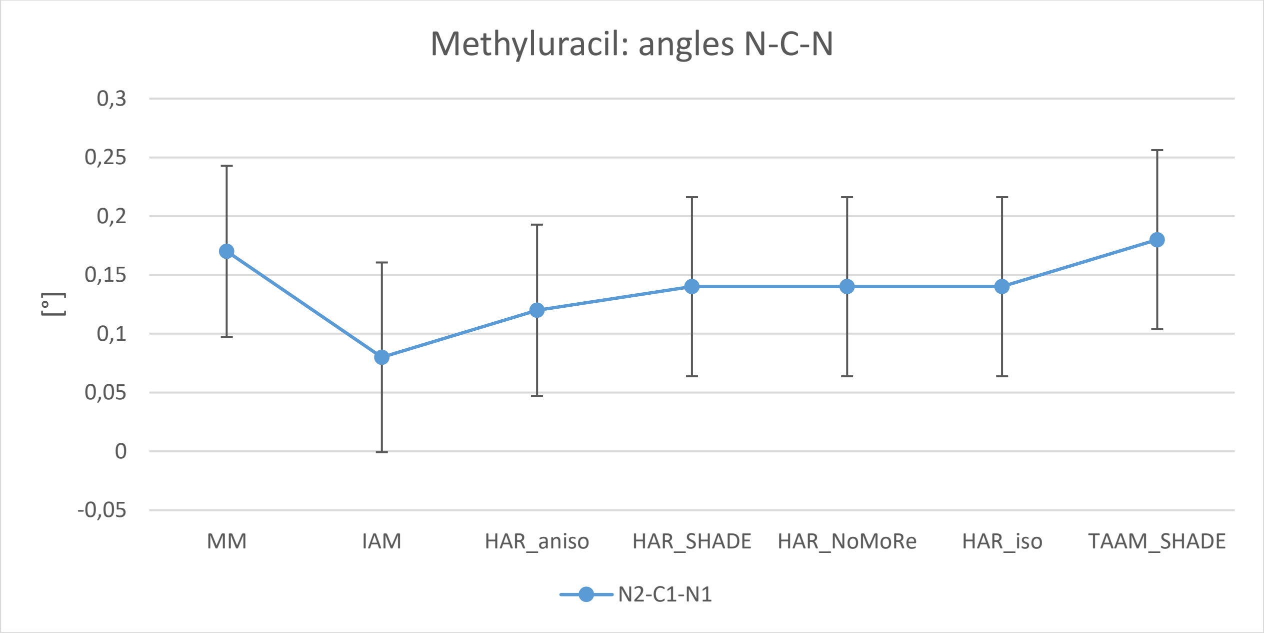


**Figure S16** Comparison of N-C-N angles for MM, IAM, HAR and TAAM refinements of **3**. Values on plot represent difference between values obtained with analysed model and neutron data in degrees. Lines on plot have no physical meaning, however help in visual analysis. Estimated standard deviations were added.

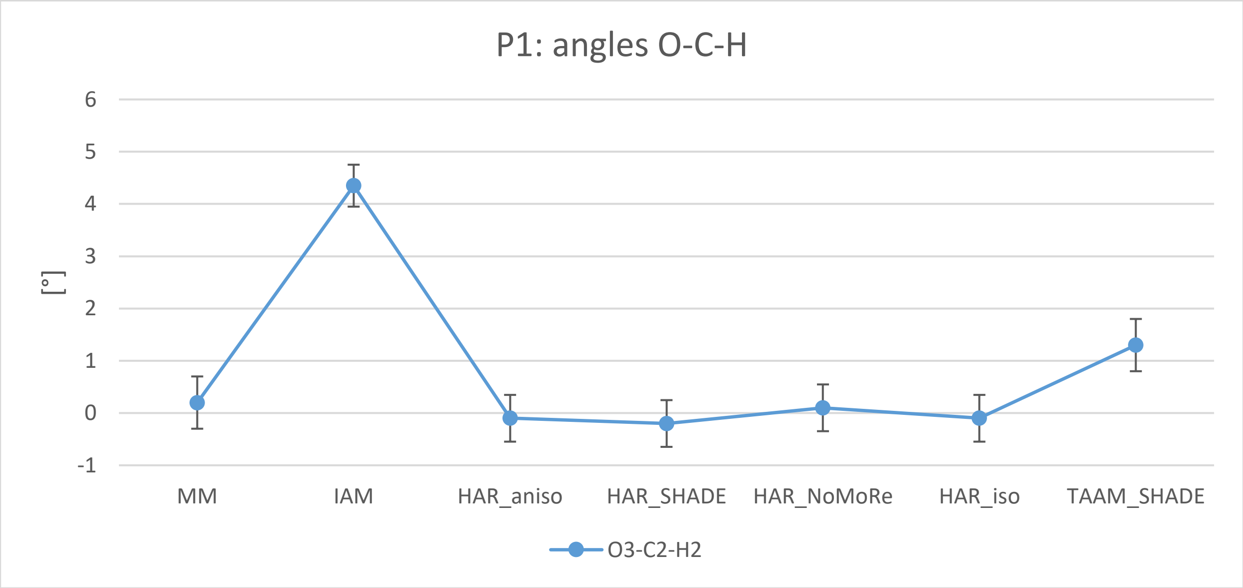

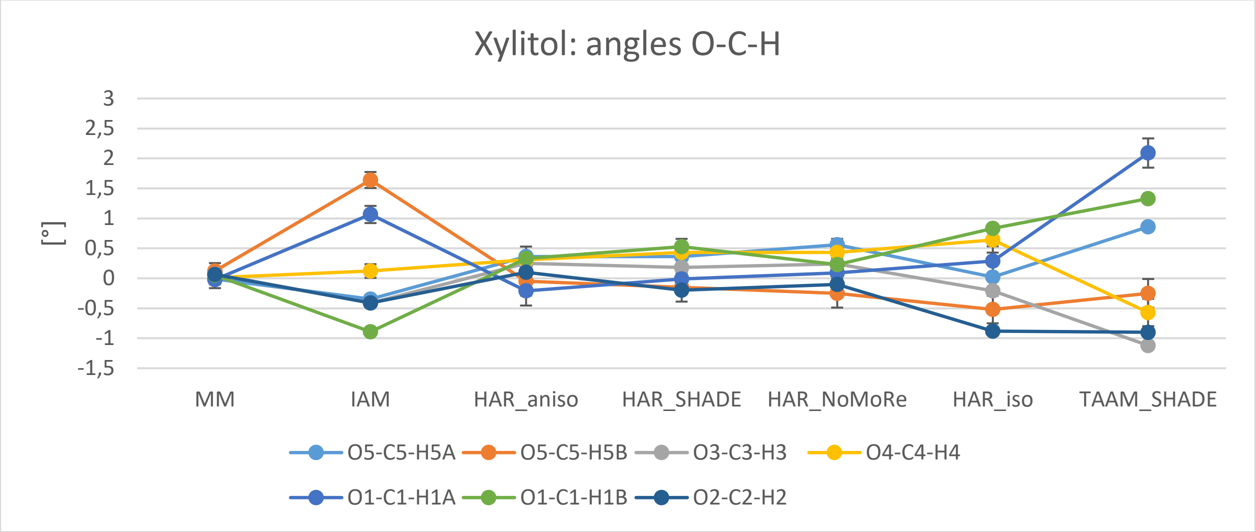


**Figure S17** Comparison of O-C-H angles for MM, IAM, HAR and TAAM refinements of **1** and **2**. Values on plot represent difference between values obtained with analysed model and neutron data in degrees. Lines on plot have no physical meaning, however help in visual analysis. For clarity estimated standard deviations for **2** were added only for O4-C4-H4, O5-C5-H5B and O2-C2-H2 angles.

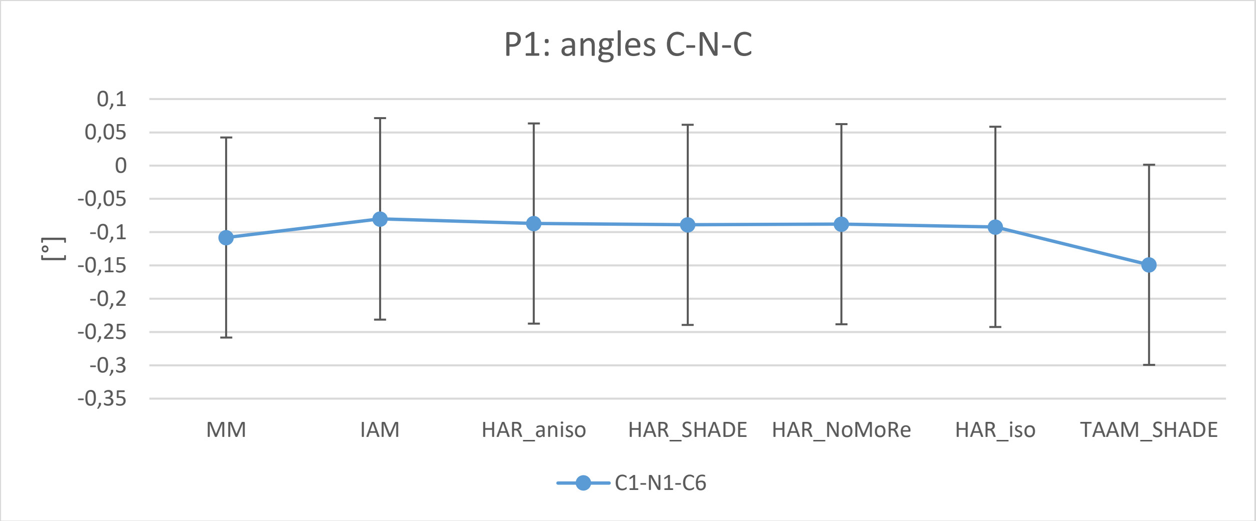

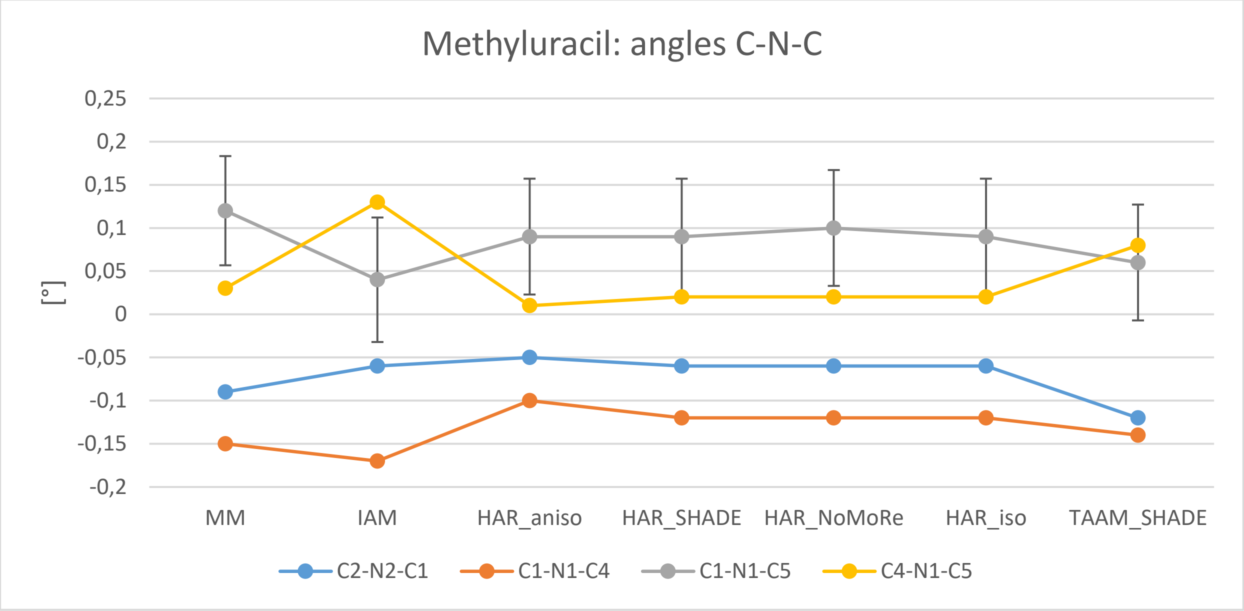


**Figure S18** Comparison of C-N-C angles for MM, IAM, HAR and TAAM refinements of **1** and **3**. Values on plot represent difference between values obtained with analysed model and neutron data in degrees. Lines on plot have no physical meaning, however help in visual analysis. For clarity estimated standard deviations for **3** were added only for C1-N1-C5 angles.

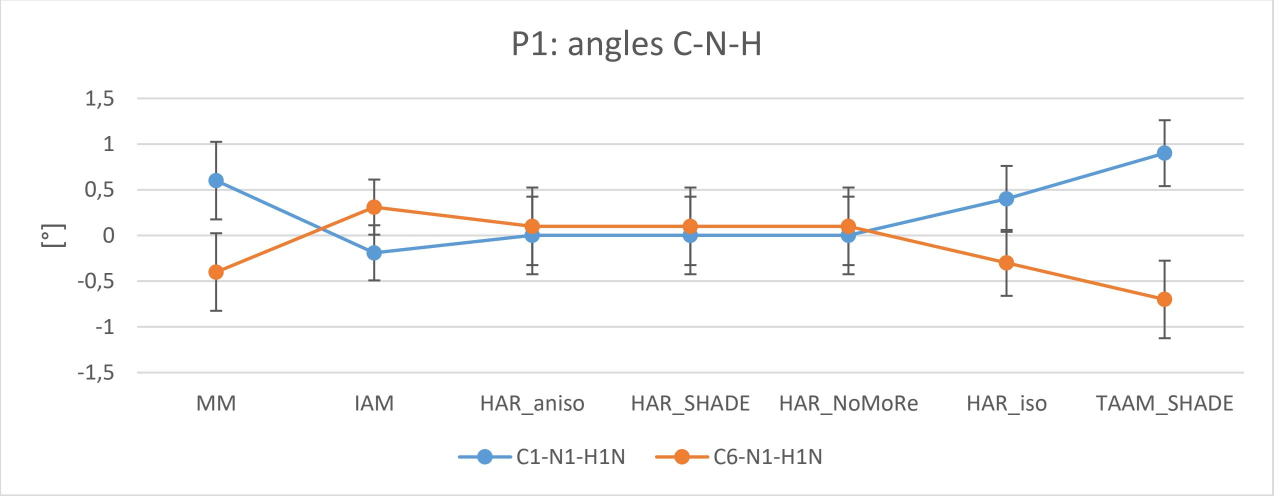

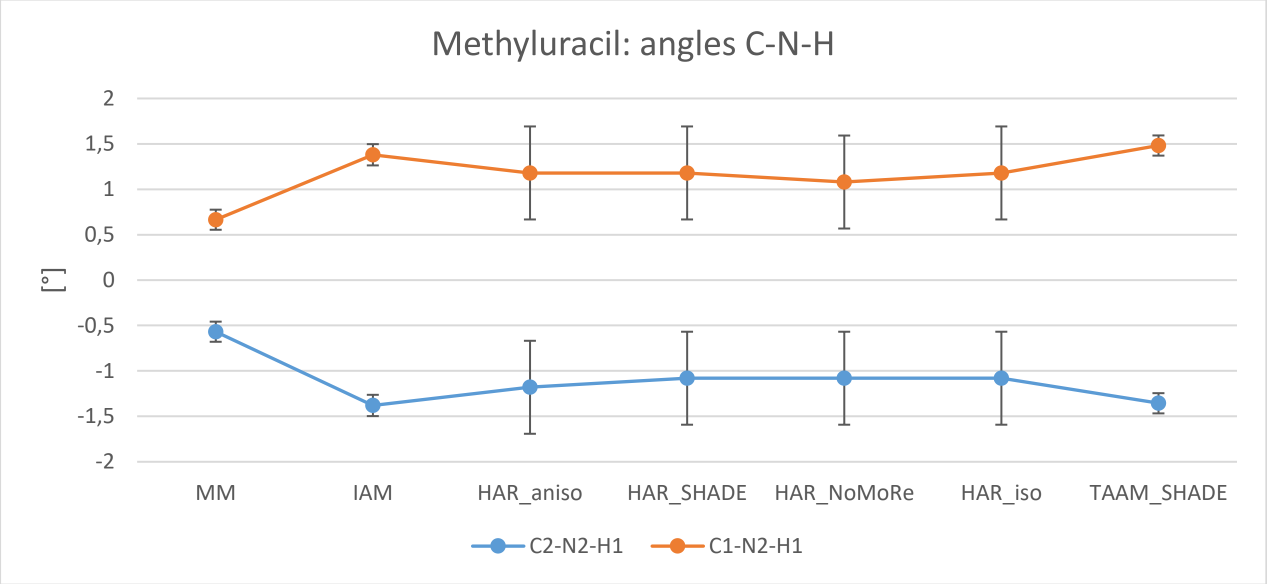


**Figure S19** Comparison of C-N-H angles for MM, IAM, HAR and TAAM refinements of **1** and **3**. Values on plot represent difference between values obtained with analysed model and neutron data in degrees. Lines on plot have no physical meaning, however help in visual analysis. For each plot estimated standard deviations were added.

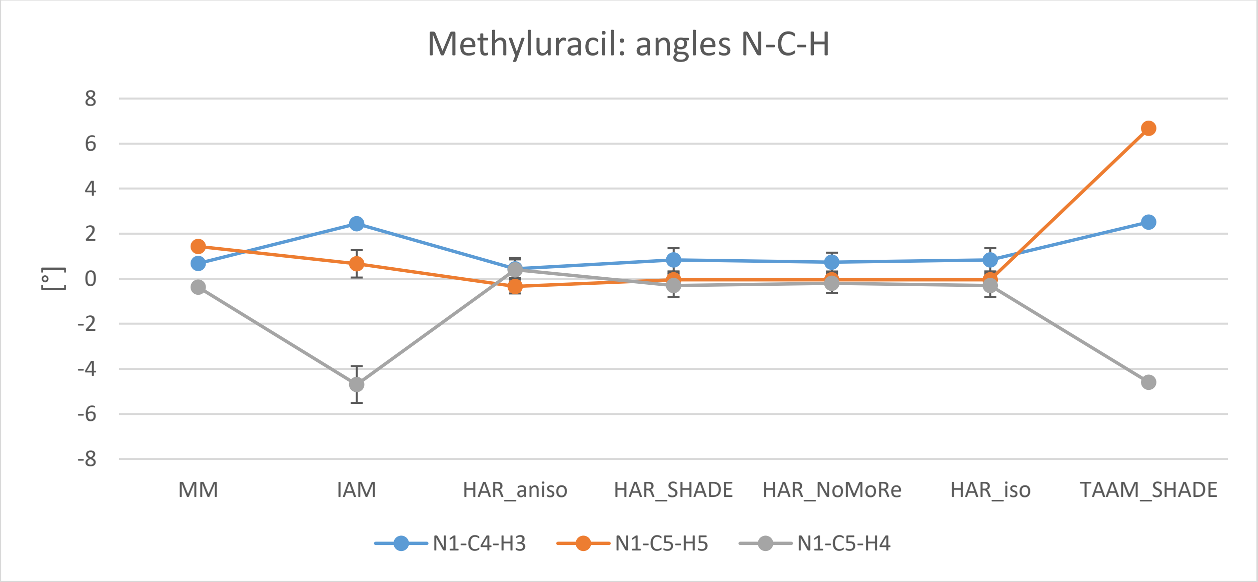


**Figure S20** Comparison of N-C-H angles for MM, IAM, HAR and TAAM refinements of **3**. Values on plot represent difference between values obtained with analysed model and neutron data in degrees. Lines on plot have no physical meaning, however help in visual analysis. For each plot estimated standard deviations were added.

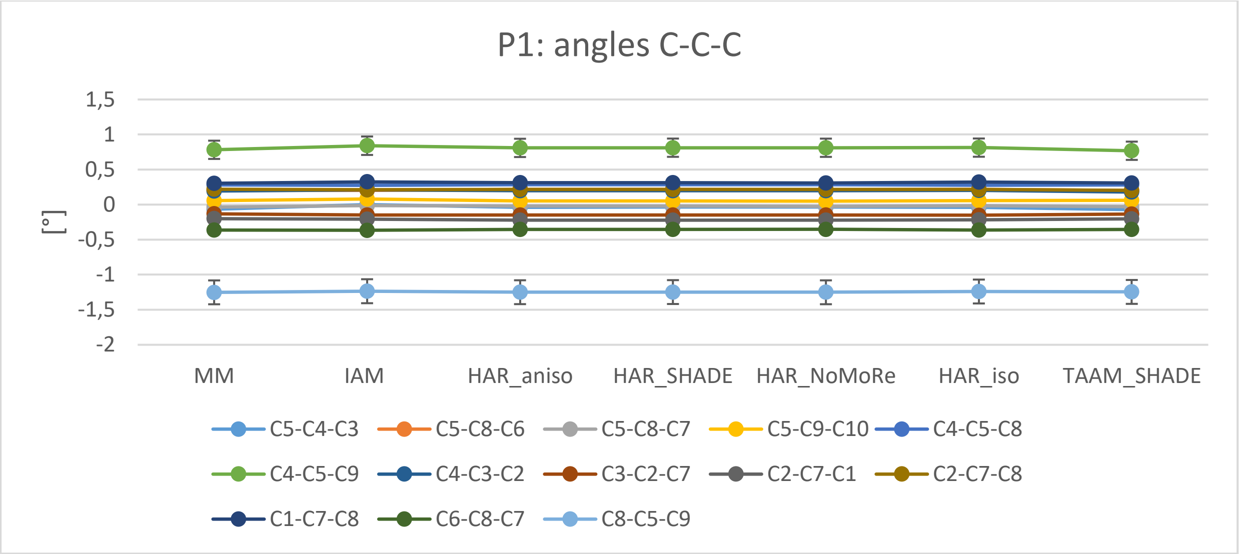
P1: angles C-C-C
1,5
1
0,5
0
-0,5
-1
-1,5
-2
[°]
MM
IAM
HAR_aniso
HAR_SHADE
HAR_NoMoRe
HAR_iso
TAAM_SHADE
C5-C4-C3
C5-C8-C6
C5-C8-C7
C5-C9-C10
C4-C5-C8
C4-C5-C9
C4-C3-C2
C3-C2-C7
C2-C7-C1
C2-C7-C8
C1-C7-C8
C6-C8-C7
C8-C5-C9

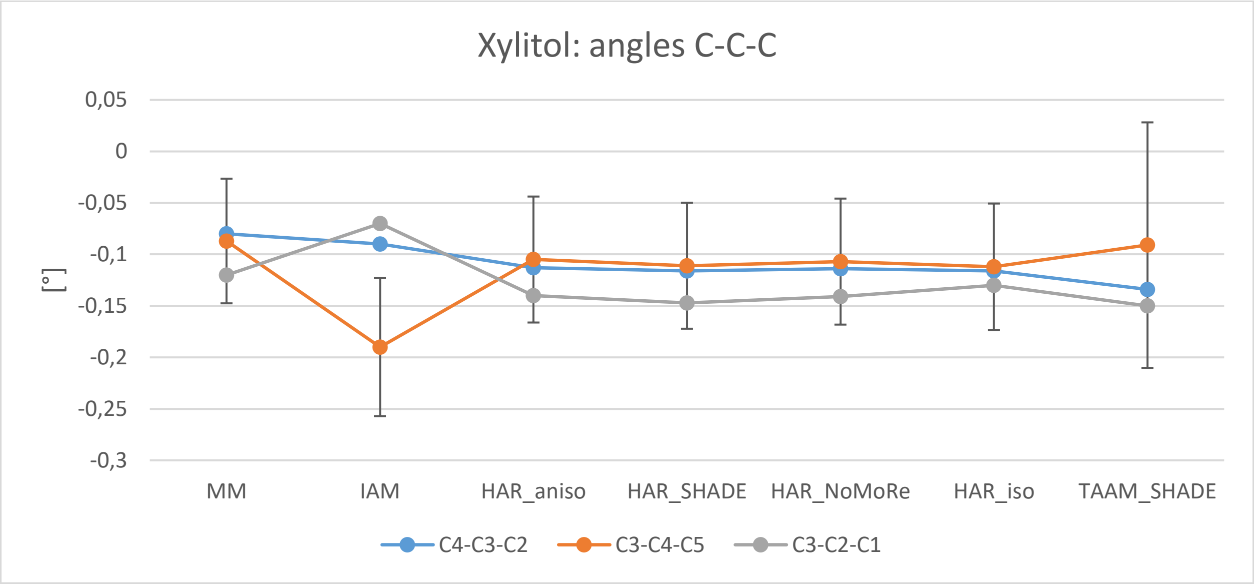
Xylitol: angles C-C-C
0,05
0
-0,05
-0,1
-0,15
-0,2
-0,25
-0,3
[°]
MM
IAM
HAR_aniso
HAR_SHADE
HAR_NoMoRe
HAR_iso
TAAM_SHADE
C4-C3-C2
C3-C4-C5
C3-C2-C1

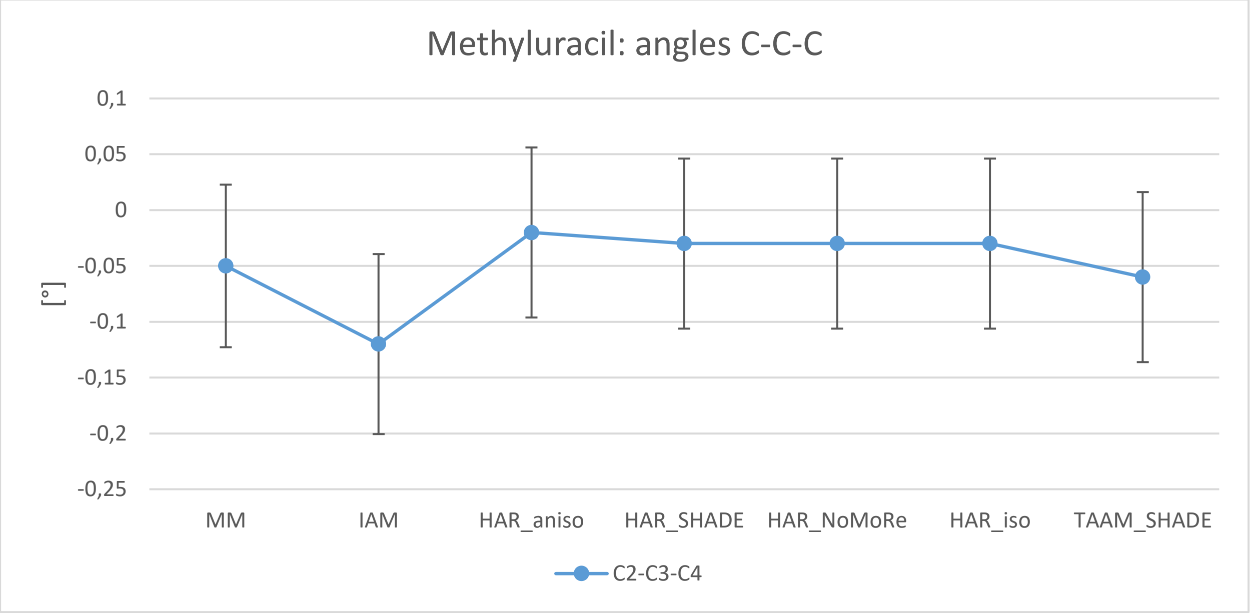


**Figure S21** Comparison of C-C-C angles for MM, IAM, HAR and TAAM refinements of **1**, **2** and **3**. Values on plot represent difference between values obtained with analysed model and neutron data in degrees. Lines on plot have no physical meaning, however help in visual analysis. For clarity estimated standard deviations for **1** were added only for C4-C5-C9 and C8-C5-C9 angles, whereas for **2** only for C3-C4-C5 angles.

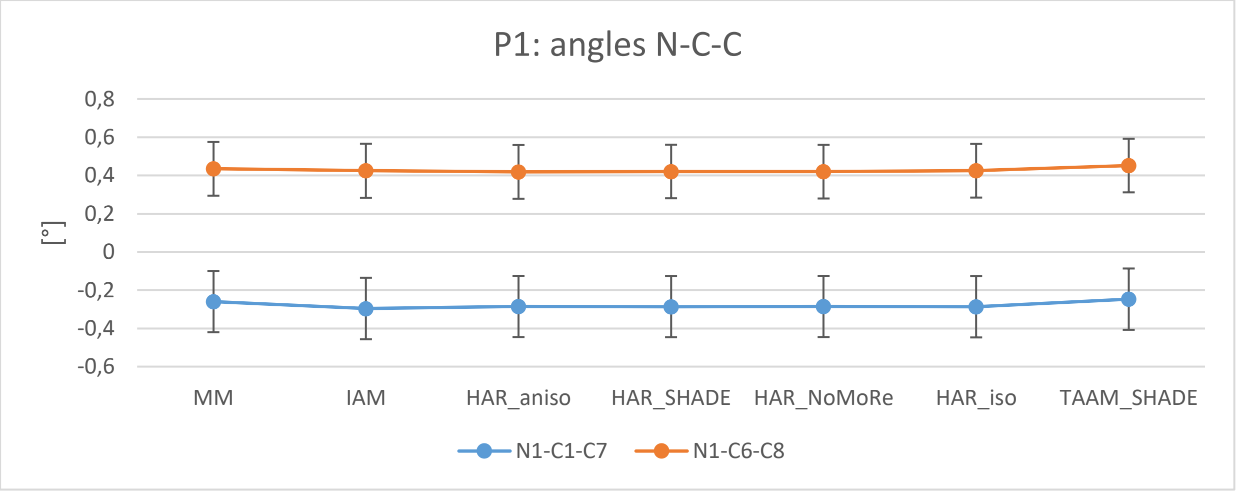

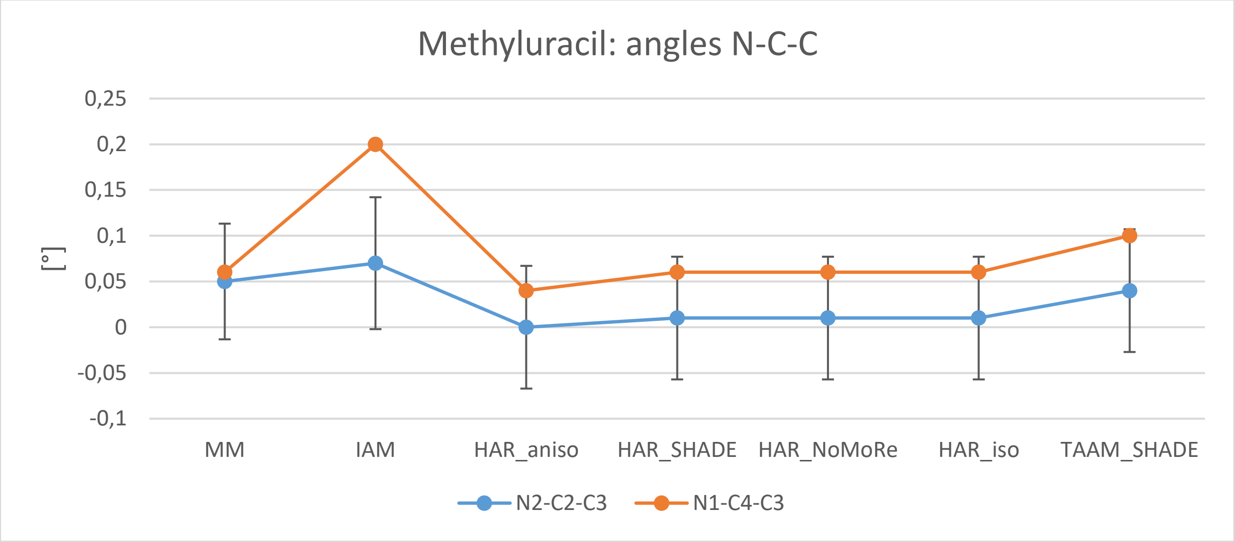


**Figure S22** Comparison of N-C-C angles for MM, IAM, HAR and TAAM refinements of **1** and **3**. Values on plot represent difference between values obtained with analysed model and neutron data in degrees. Lines on plot have no physical meaning, however help in visual analysis. For each plot estimated standard deviations were added.

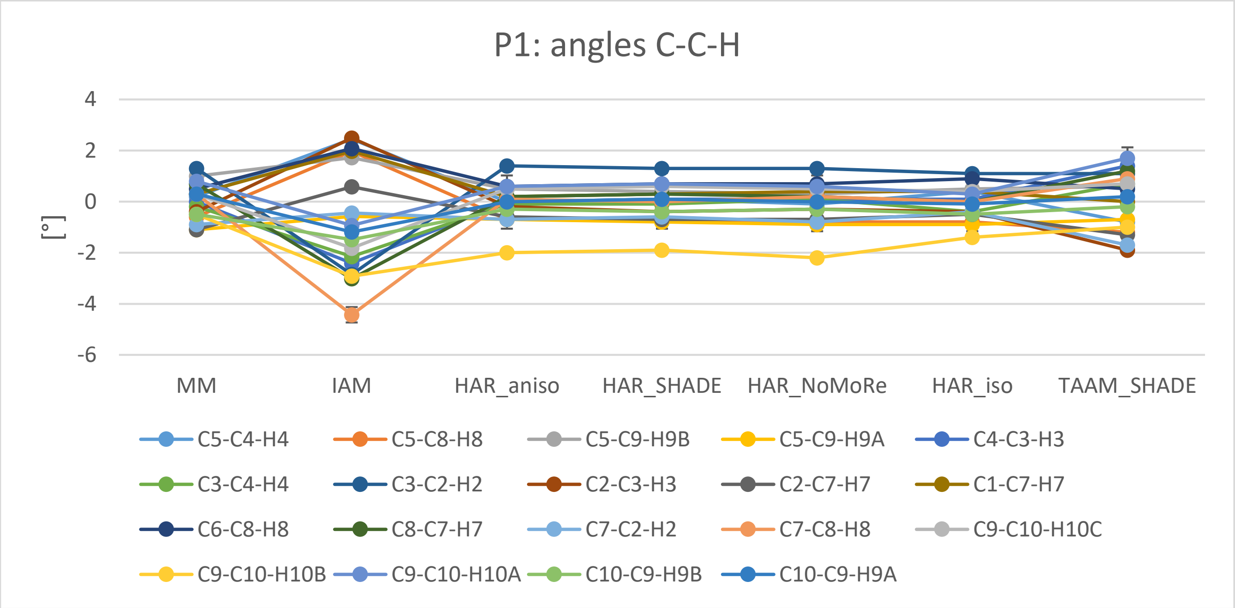

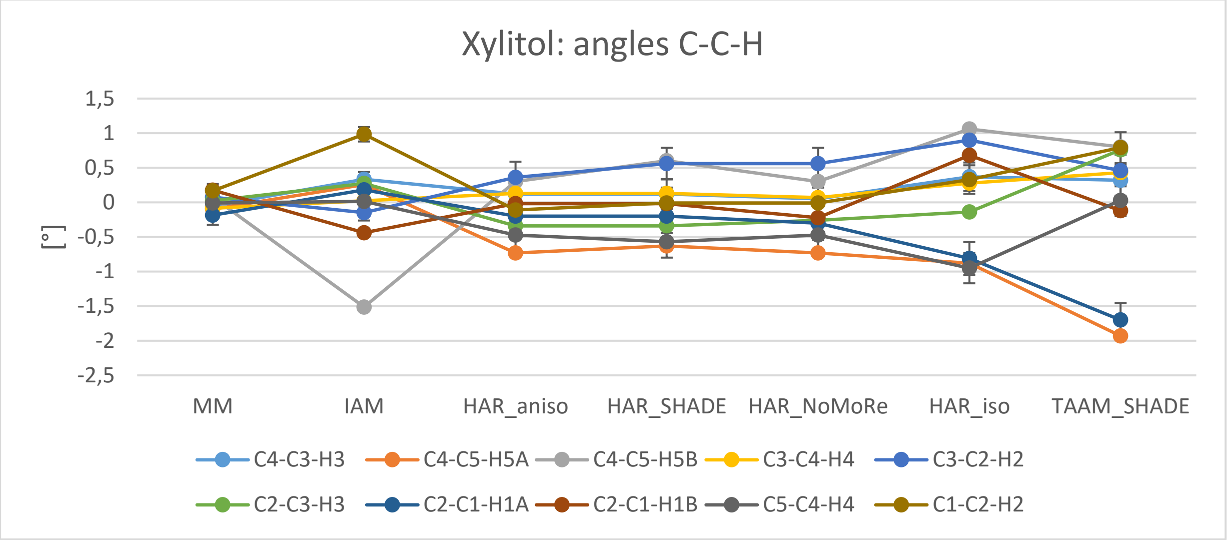


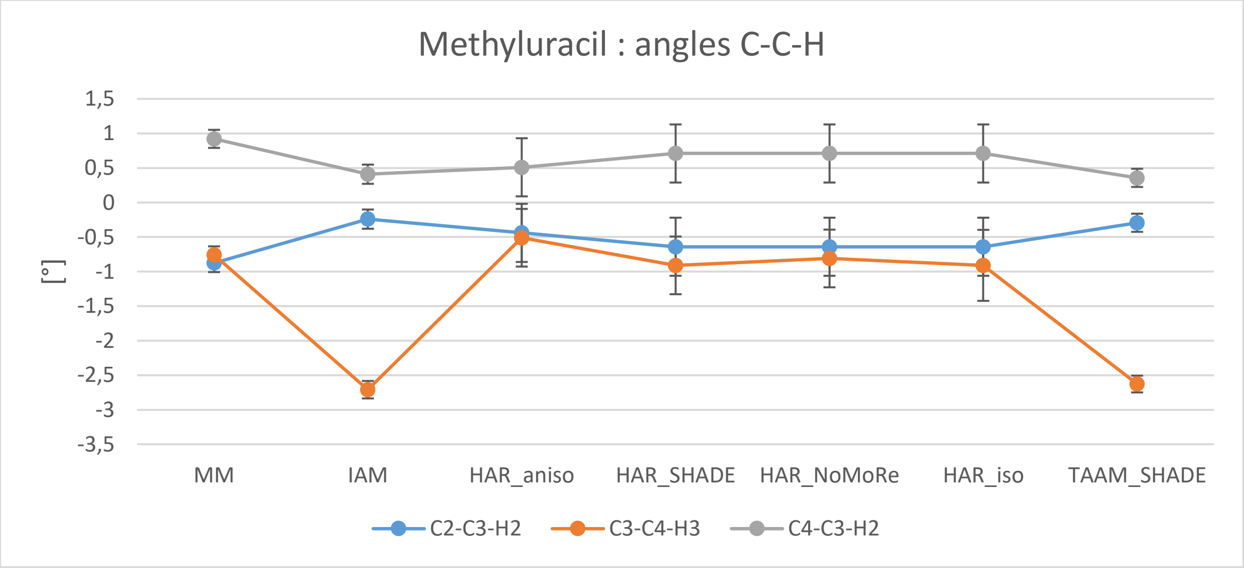


**Figure S23** Comparison of C-C-H angles for MM, IAM, HAR and TAAM refinements of **1**, **2** and **3**. Values on plot represent difference between values obtained with analysed model and neutron data in degrees. Lines on plot have no physical meaning, however help in visual analysis. For each plot estimated standard deviations were added.

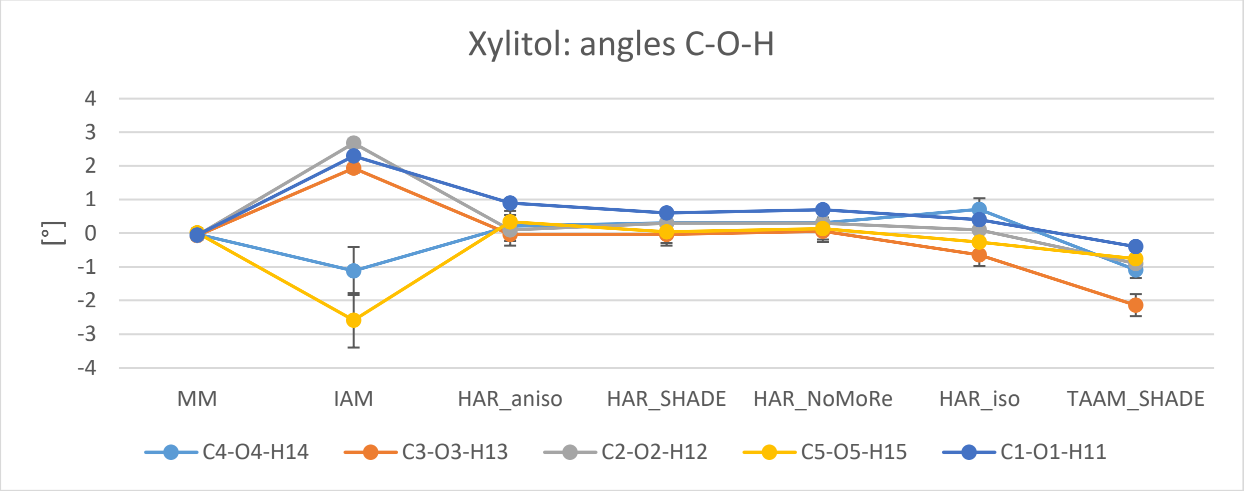


**Figure S24** Comparison of C-O-H angles for MM, IAM, HAR and TAAM refinements of **2**. Values on plot represent difference between values obtained with analysed model and neutron data in degrees. Lines on plot have no physical meaning, however help in visual analysis. For each plot estimated standard deviations were added.

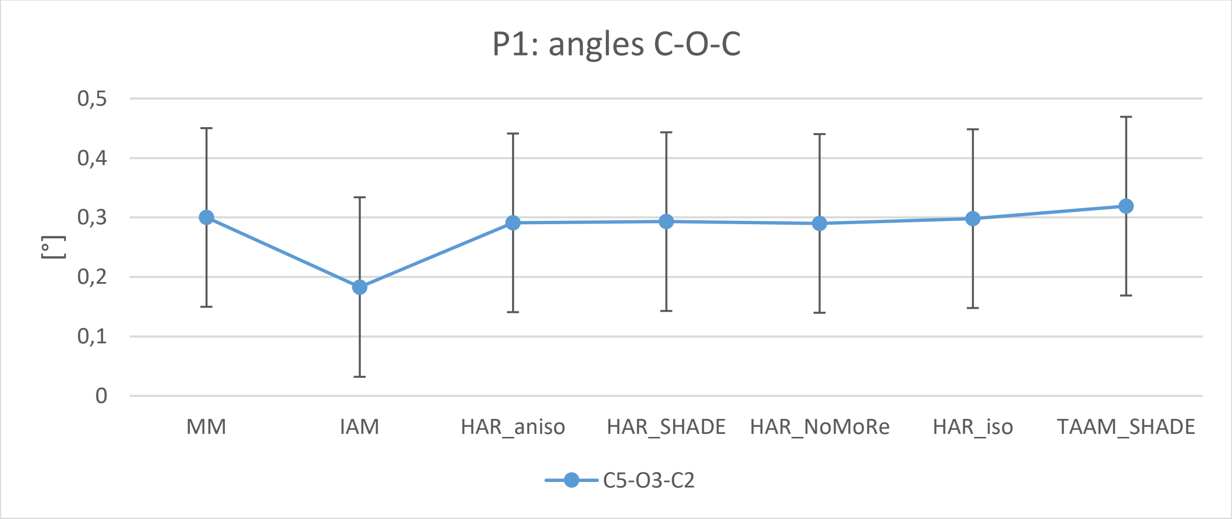


**Figure S25** Comparison of C-O-C angles for MM, IAM, HAR and TAAM refinements of **1**. Values on plot represent difference between values obtained with analysed model and neutron data in degrees. Lines on plot have no physical meaning, however help in visual analysis. Estimated standard deviations were added.

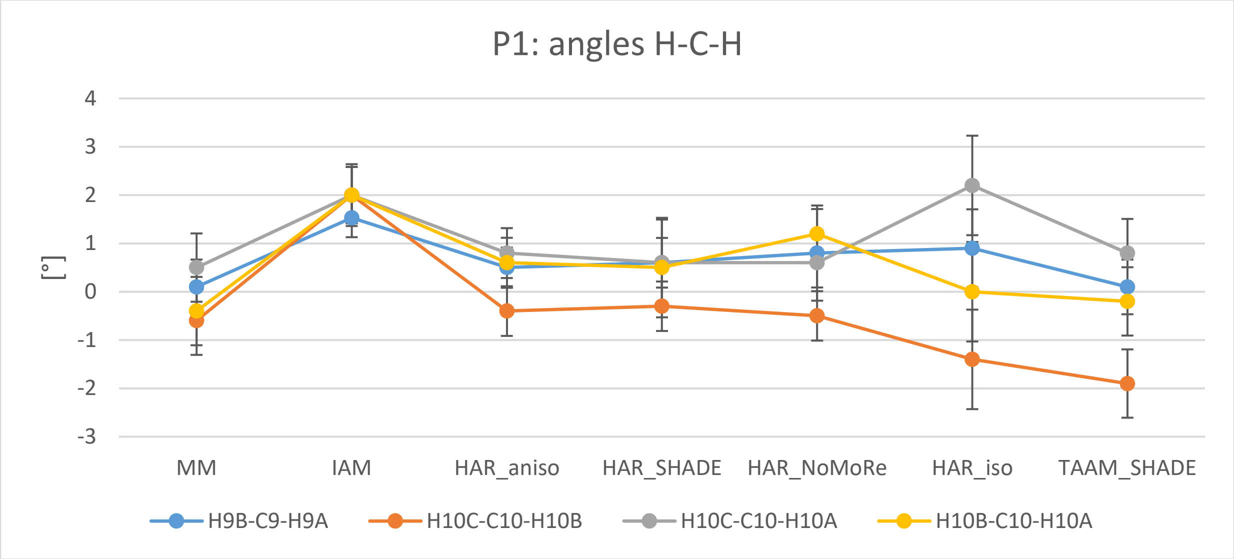
P1: angles H-C-H
[°]
4
3
2
1
0
-1
-2
-3
MM
IAM
HAR_aniso
HAR_SHADE
HAR_NoMoRe
HAR_iso
TAAM_SHADE
H9B-C9-H9A
H10C-C10-H10B
H10C-C10-H10A
H10B-C10-H10A

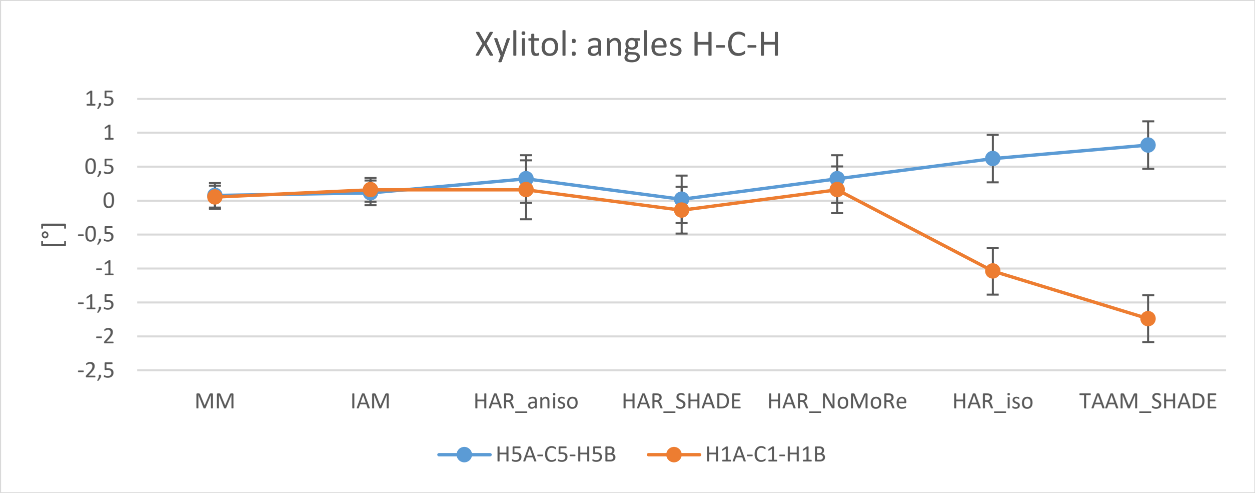
Xylitol: angles H-C-H
[°]
1,5
1
0,5
0
-0,5
-1
-1,5
-2
-2,5
MM
IAM
HAR_aniso
HAR_SHADE
HAR_NoMoRe
HAR_iso
TAAM_SHADE
H5A-C5-H5B
H1A-C1-H1B

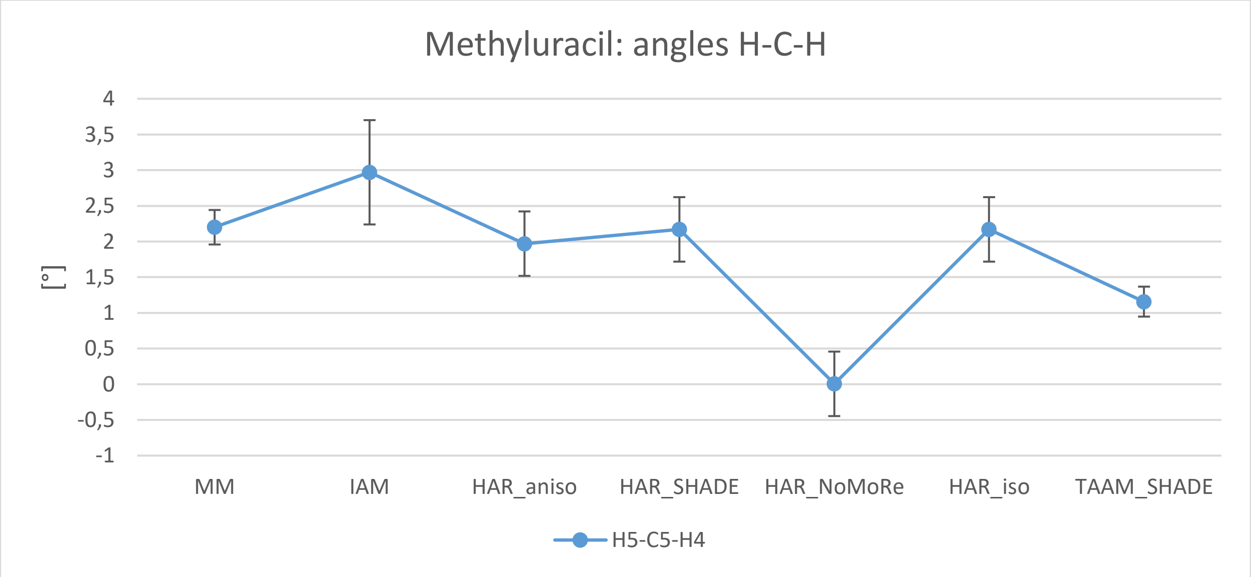
Methyluracil: angles H-C-H
[°]
4
3,5
3
2,5
2
1,5
1
0,5
0
-0,5
-1
MM
IAM
HAR_aniso
HAR_SHADE
HAR_NoMoRe
HAR_iso
TAAM_SHADE
H5-C5-H4

**Figure S26** Comparison of H-C-H angles for MM, IAM, HAR and TAAM refinements of **1**, **2** and **3**. Values on plot represent difference between values obtained with analysed model and neutron data in degrees. Lines on plot have no physical meaning, however help in visual analysis. For each plot estimated standard deviations were added.

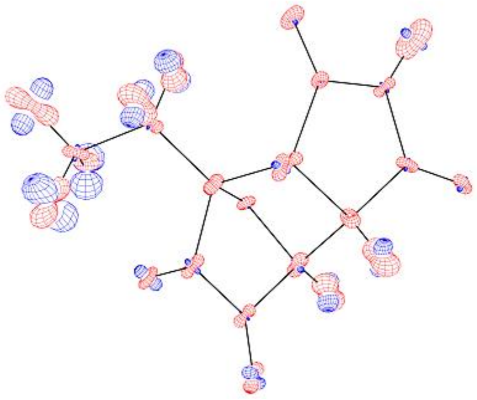

MM

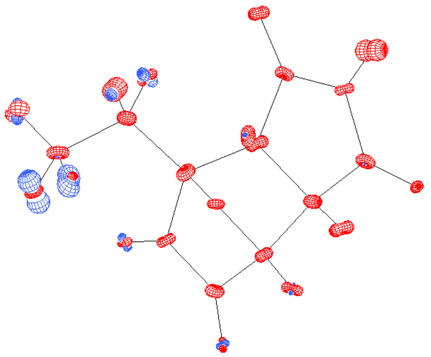

TAAM_Shade

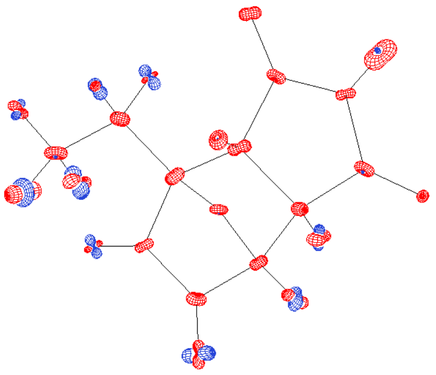

HAR_aniso

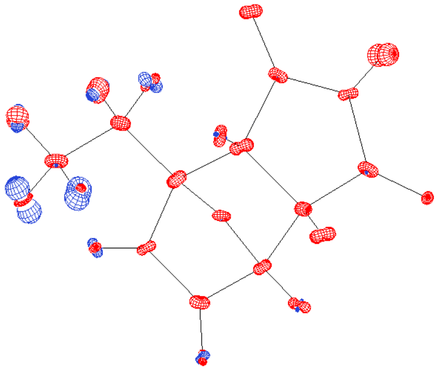

HAR_Shade

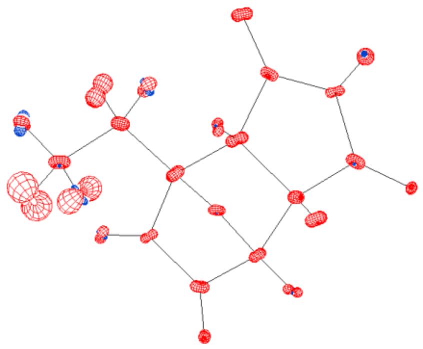

HAR_NoMoRe

**Figure S27** Difference between ADPs of neutron and analysed model for MM, HAR and TAAM refinements of **1**. Two-step overlay algorithm and 2.0 scale were applied using PEANUT software.

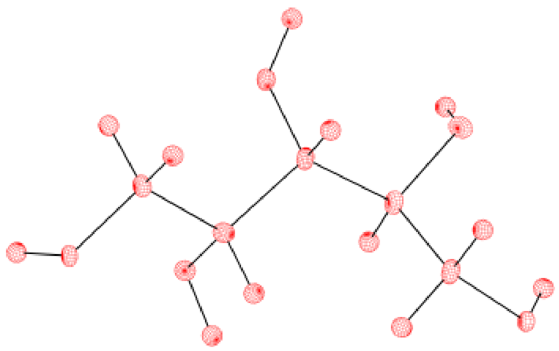

MM

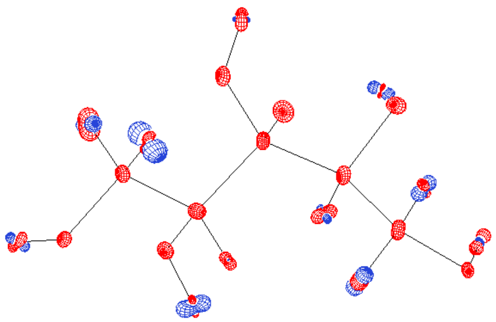

TAAM_Shade

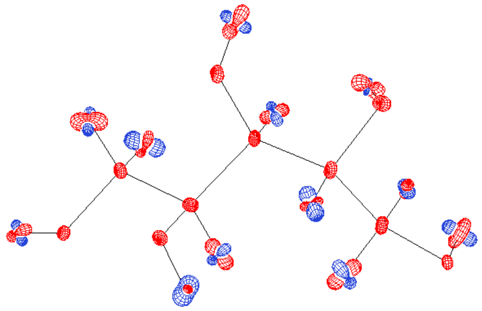

HAR_aniso

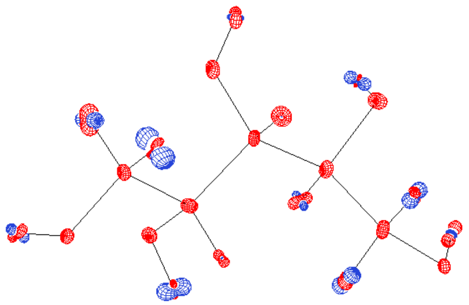

HAR_Shade

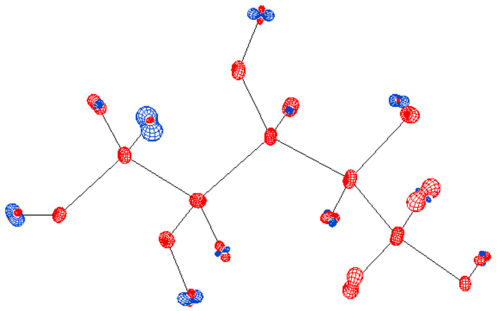

HAR_NoMoRe

**Figure S28** Difference between ADPs of neutron and analysed model for MM, HAR and TAAM refinements of **2**. Two-step overlay algorithm and 2.0 scale were applied using PEANUT software.

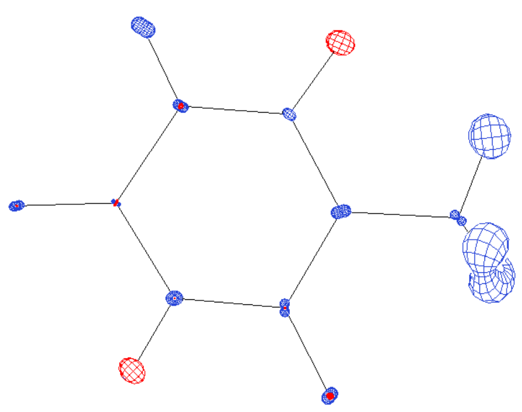

MM

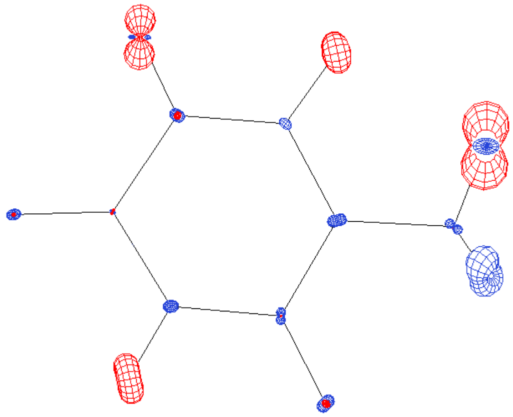

TAAM_Shade

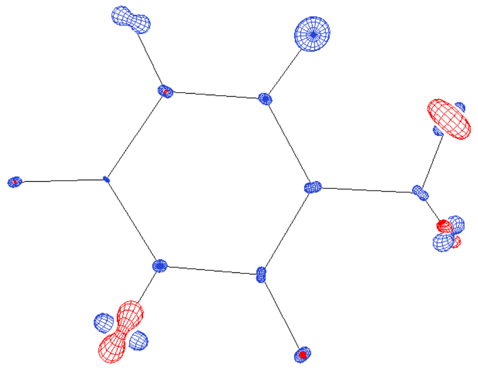

HAR_aniso

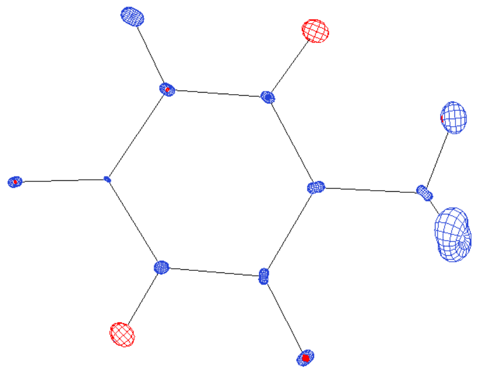

HAR_Shade

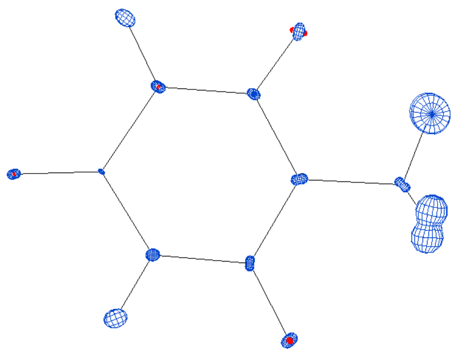

HAR_NoMoRe

**Figure S29** Difference between ADPs of neutron and analysed model for MM, HAR and TAAM refinements of **3**. Two-step overlay algorithm and 2.0 scale were applied using PEANUT software.

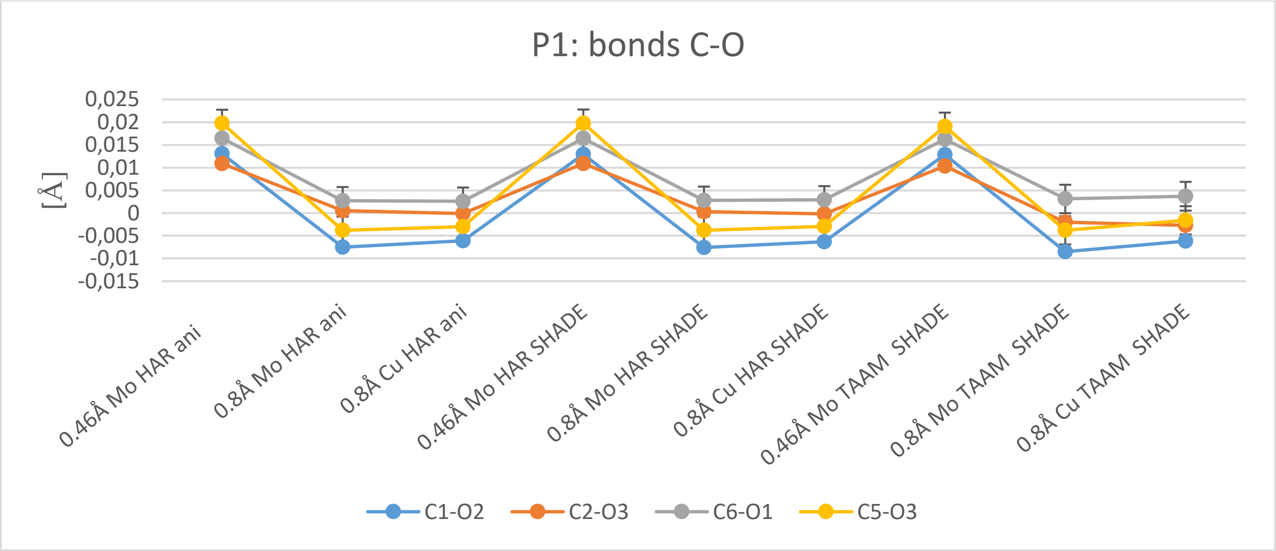

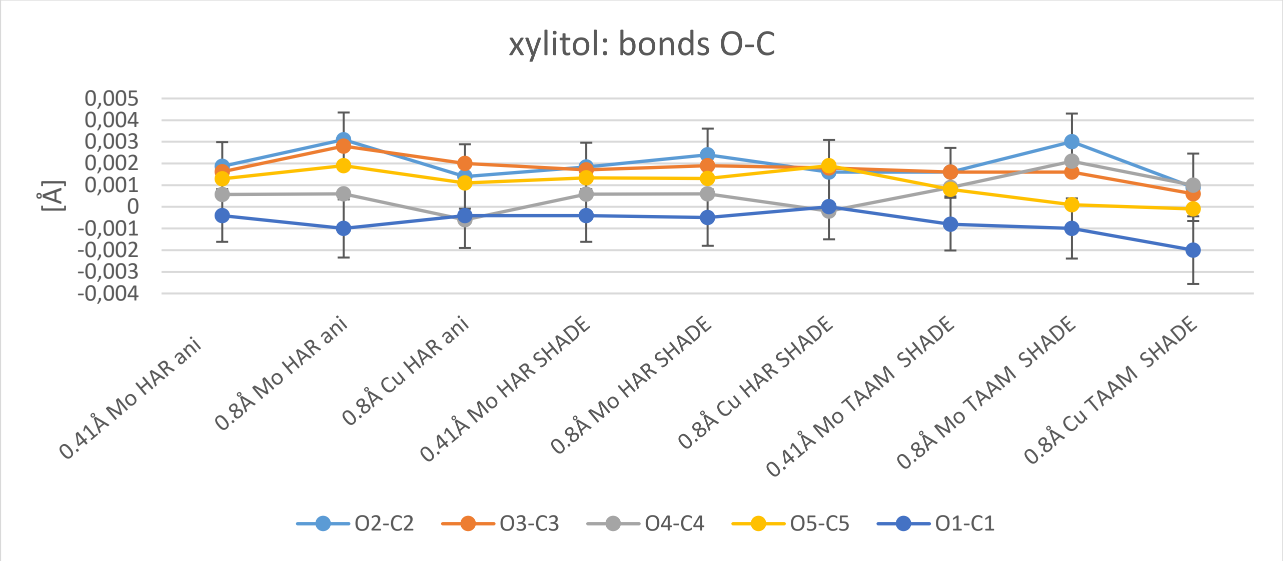


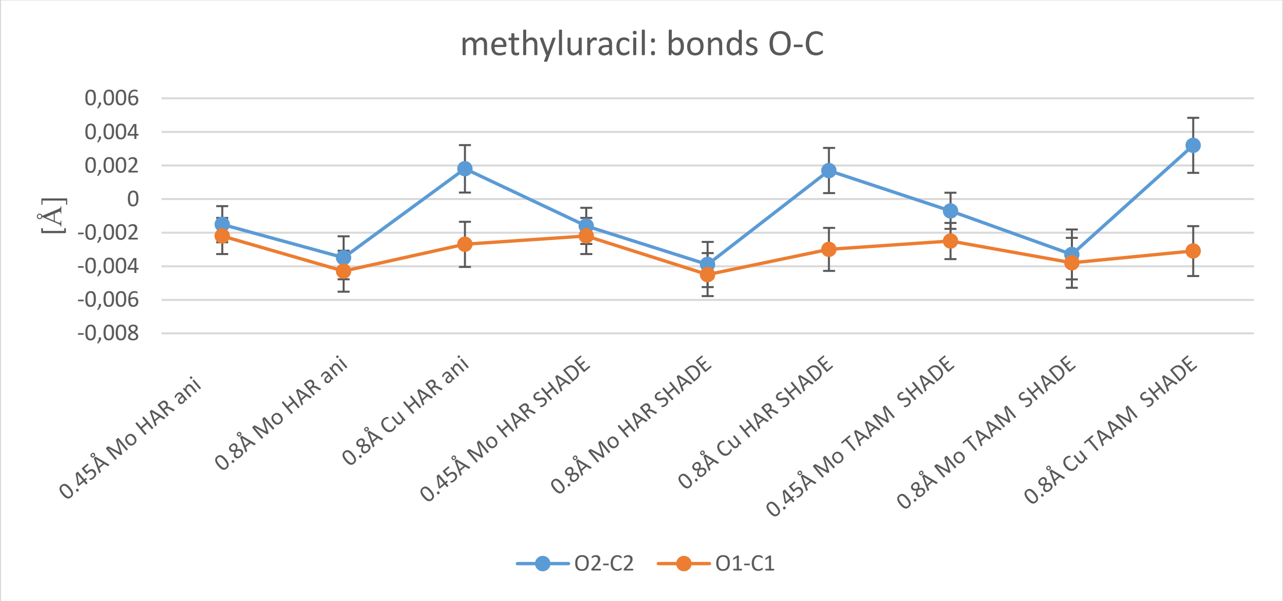


**Figure S30** Comparison of O-C bonds for HAR and TAAM refinements of **1**, **2**, **3**, **1-cutoff**, **2-cutoff** and **3-cutoff**, **1-CuKα**, **2-CuKα** and **3CuKα**. Values on plot represent difference between values obtained with analysed model and neutron data in angstroms. Lines on plot have no physical meaning, however help in visual analysis. For each plot estimated standard deviations were added.

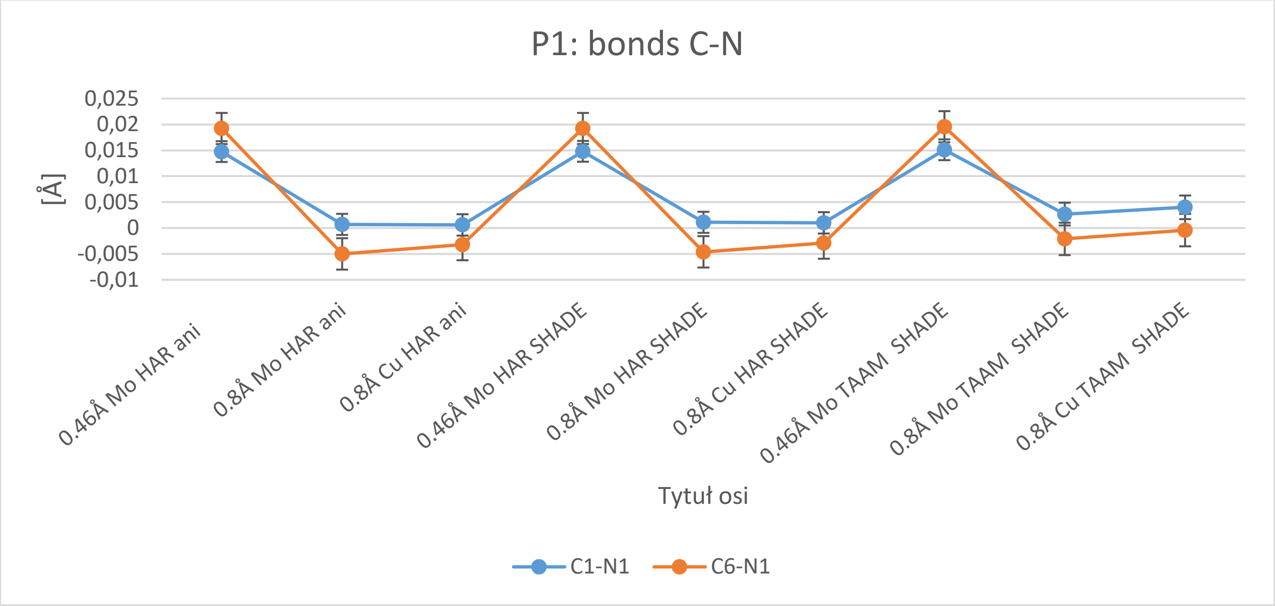


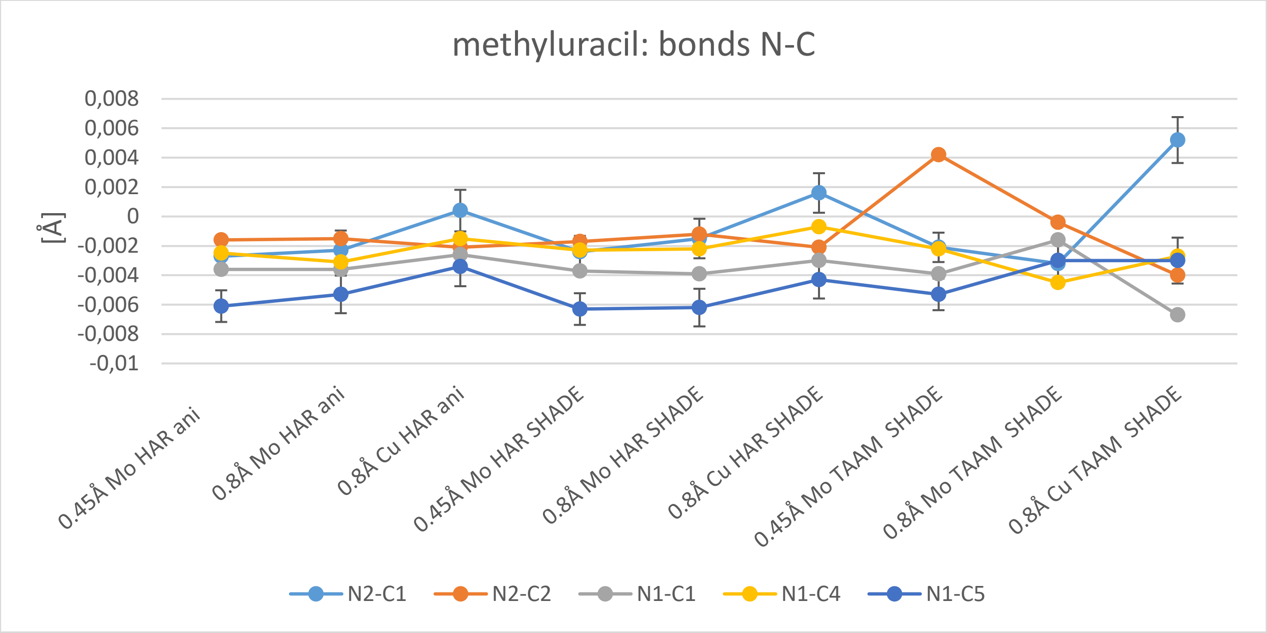


**Figure S31** Comparison of N-C bonds for HAR and TAAM refinements of **1**, **3**, **1-cutoff**, **3-cutoff**, **1-CuKα** and **3CuKα**. Values on plot represent difference between values obtained with analysed model and neutron data in angstroms. Lines on plot have no physical meaning, however help in visual analysis. For each plot of **1**, **1-cutoff, 1-CuKα** and selected plot of **3**, **3-cutoff, 3-CuKα** (N2-C1, N1-C5, N1-C4) estimated standard deviations were added.

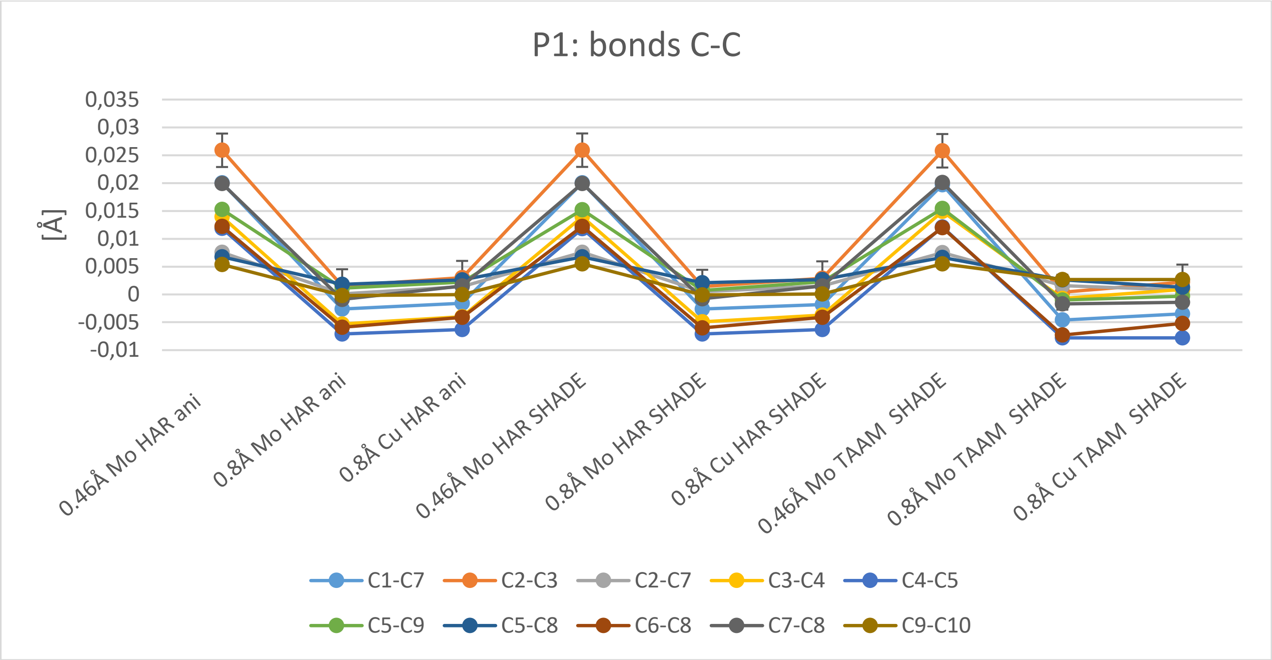
P1: bonds C-C
0,035
0,03
0,025
0,02
0,015
0,01
0,005
0
-0,005
-0,01
[Å]
0.46Å Mo HAR ani
0.8Å Mo HAR ani
0.8Å Cu HAR ani
0.46Å Mo HAR SHADE
0.8Å Mo HAR SHADE
0.8Å Cu HAR SHADE
0.46Å Mo TAAM SHADE
0.8Å Mo TAAM SHADE
0.8Å Cu TAAM SHADE
C1-C7
C2-C3
C2-C7
C3-C4
C4-C5
C5-C9
C5-C8
C6-C8
C7-C8
C9-C10

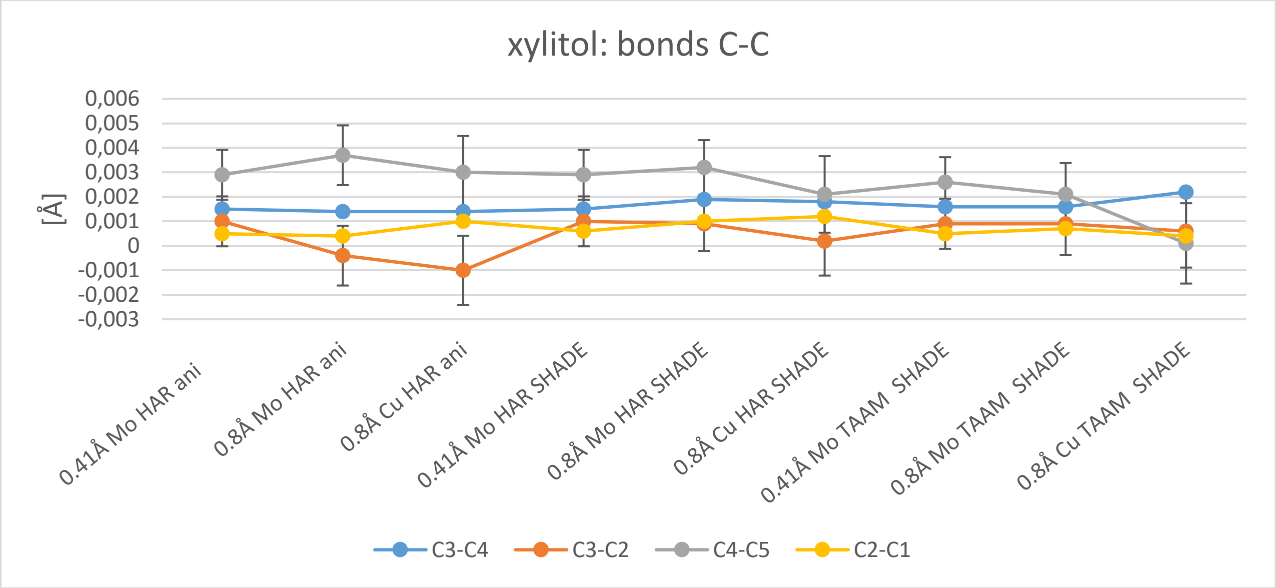
xylitol: bonds C-C
0,006
0,005
0,004
0,003
0,002
0,001
0
-0,001
-0,002
-0,003
[Å]
0.41Å Mo HAR ani
0.8Å Mo HAR ani
0.8Å Cu HAR ani
0.41Å Mo HAR SHADE
0.8Å Mo HAR SHADE
0.8Å Cu HAR SHADE
0.41Å Mo TAAM SHADE
0.8Å Mo TAAM SHADE
0.8Å Cu TAAM SHADE
C3-C4
C3-C2
C4-C5
C2-C1

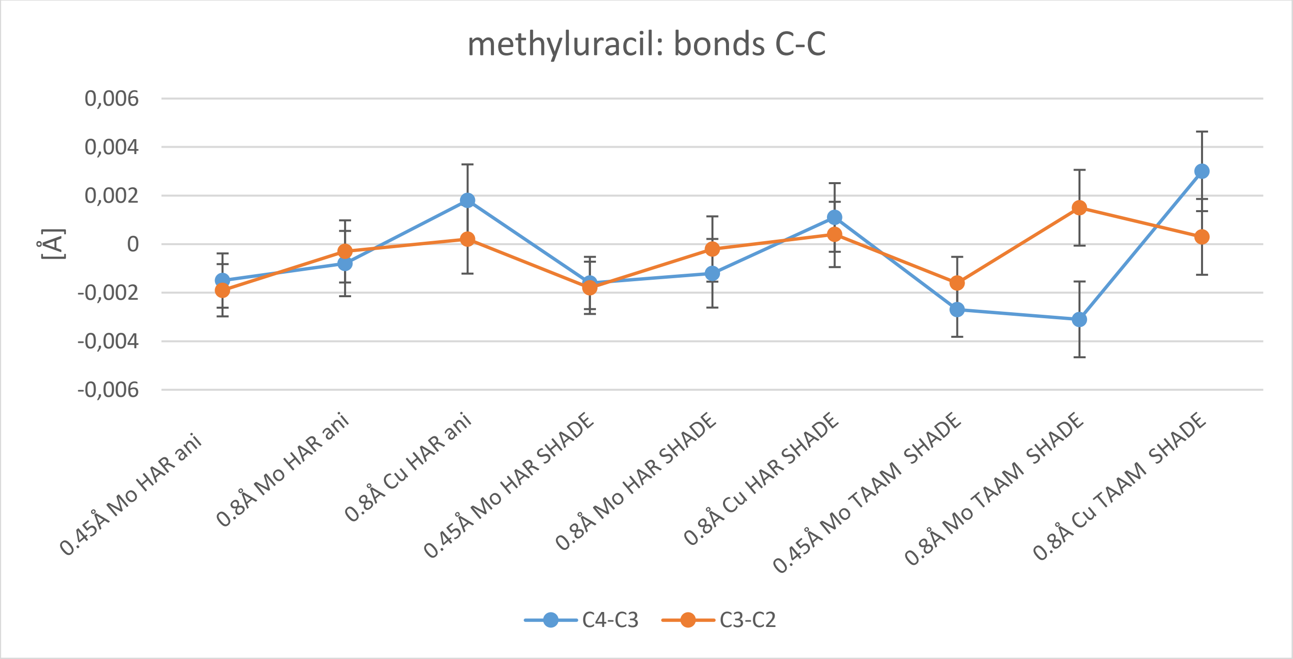


**Figure S32** Comparison of C-C bonds for HAR and TAAM refinements of **1**, **2**, **3**, **1-cutoff**, **2-cutoff** and **3-cutoff**, **1-CuKα**, **2-CuKα** and **3CuKα**. Values on plot represent difference between values obtained with analysed model and neutron data in angstroms. Lines on plot have no physical meaning, however help in visual analysis. For clarity, estimated standard deviations were added to the selected plots.

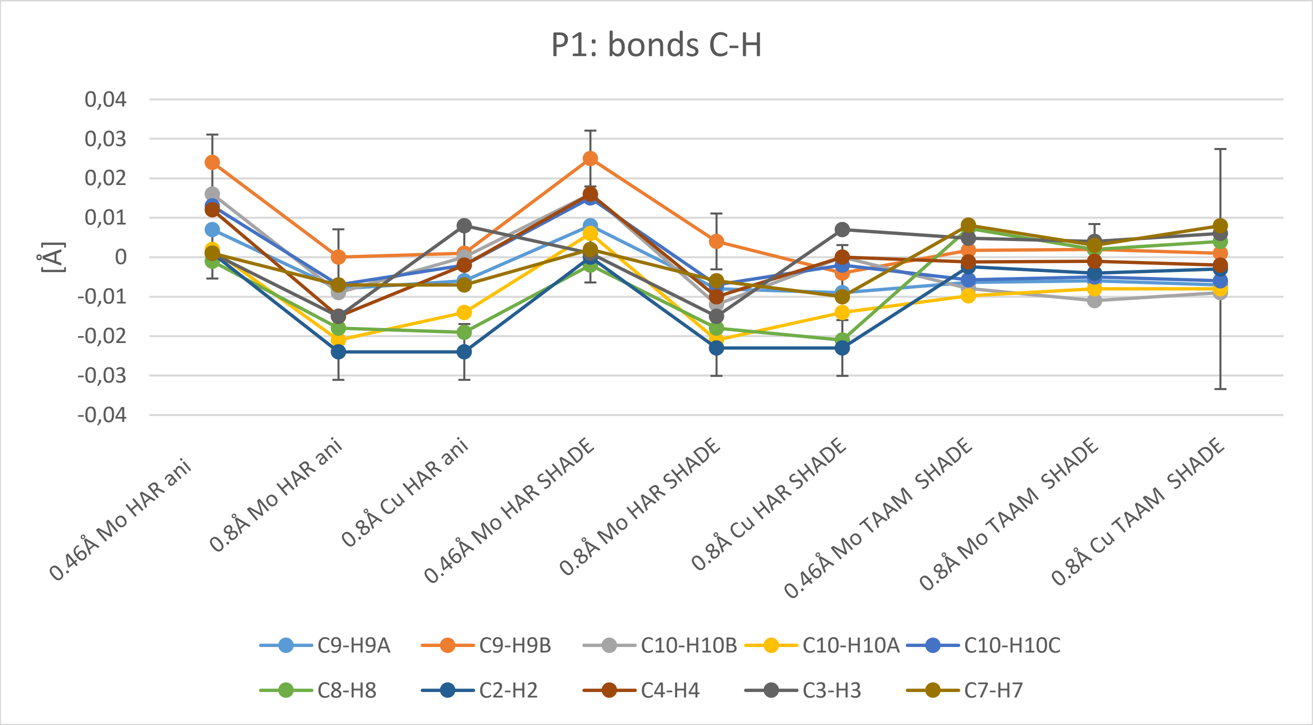

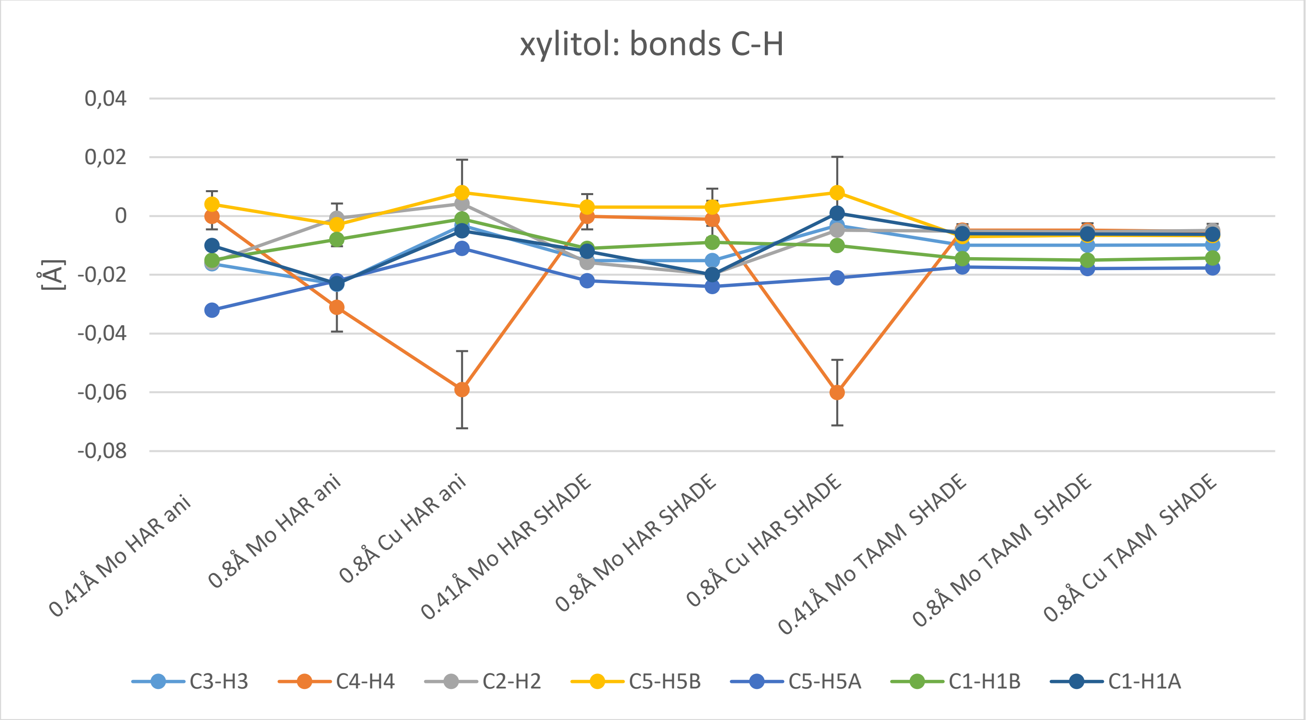


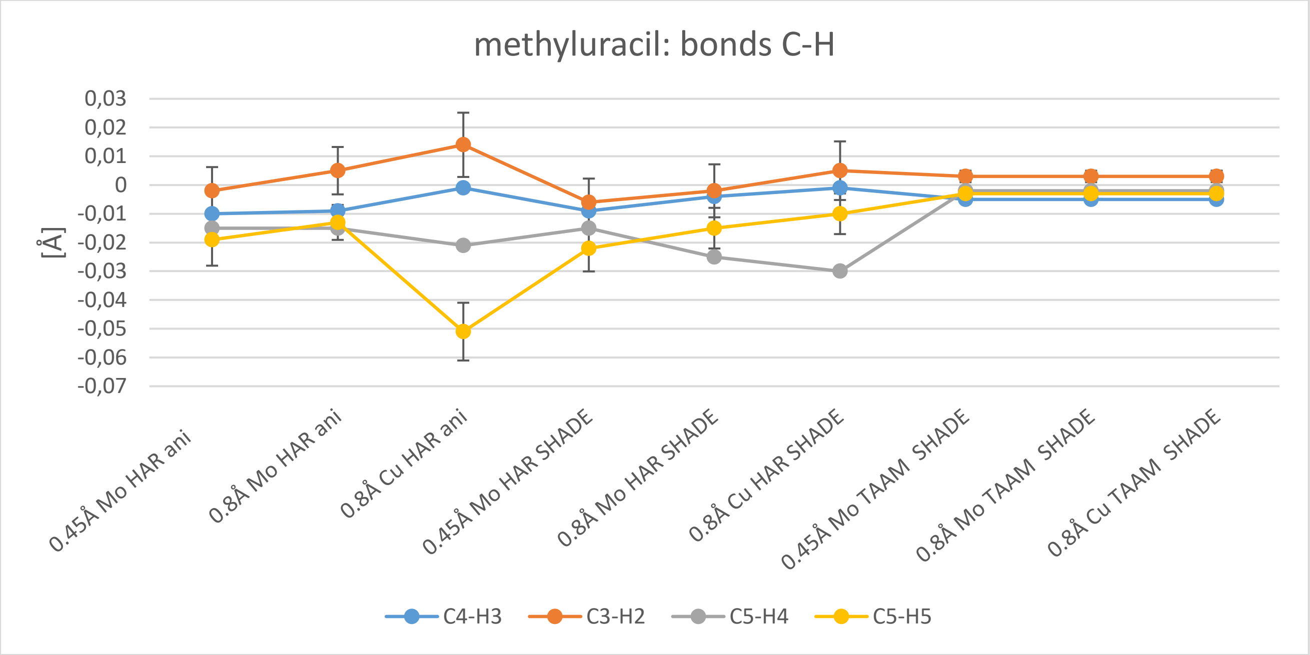


**Figure S33** Comparison of C-H bonds for HAR and TAAM refinements of **1**, **2**, **3**, **1-cutoff**, **2-cutoff** and **3-cutoff**, **1-CuKα**, **2-CuKα** and **3CuKα**. Values on plot represent difference between values obtained with analysed model and neutron data in angstroms. Lines on plot have no physical meaning, however help in visual analysis. For clarity, estimated standard deviations were added to the selected plots.

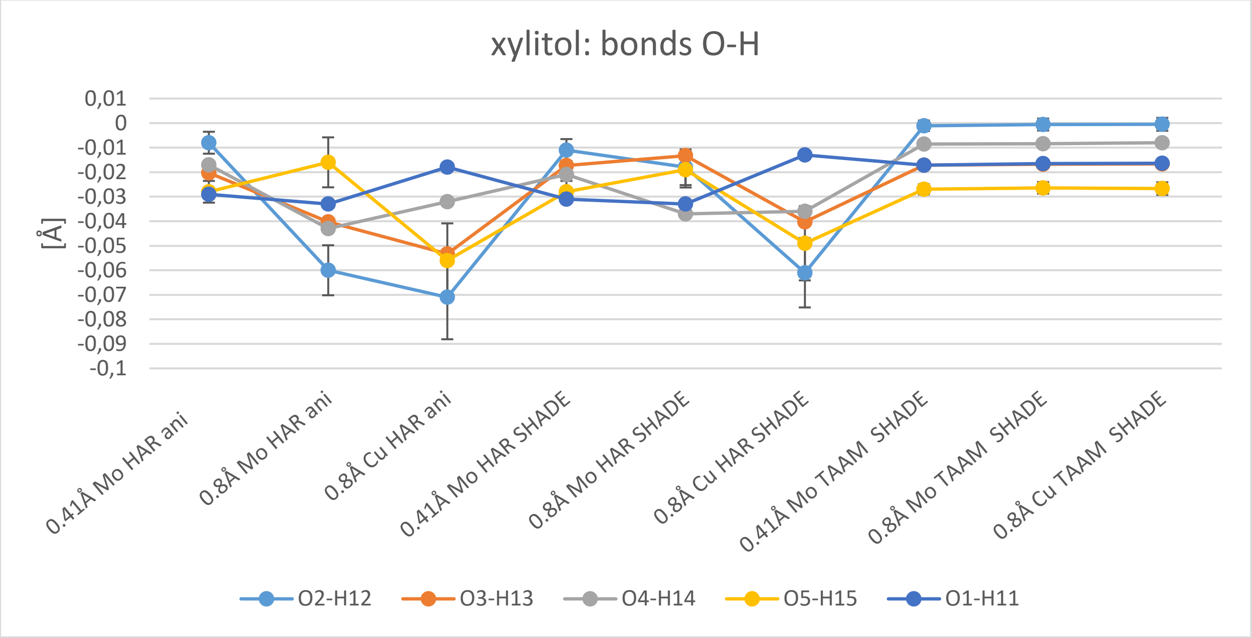


**Figure S34** Comparison of O-H bonds for HAR and TAAM refinements of **2**, **2-cutoff** and **2-CuKα**. Values on plot represent difference between values obtained with analysed model and neutron data in angstroms. Lines on plot have no physical meaning, however help in visual analysis. For clarity, estimated standard deviations were added to the selected plots.

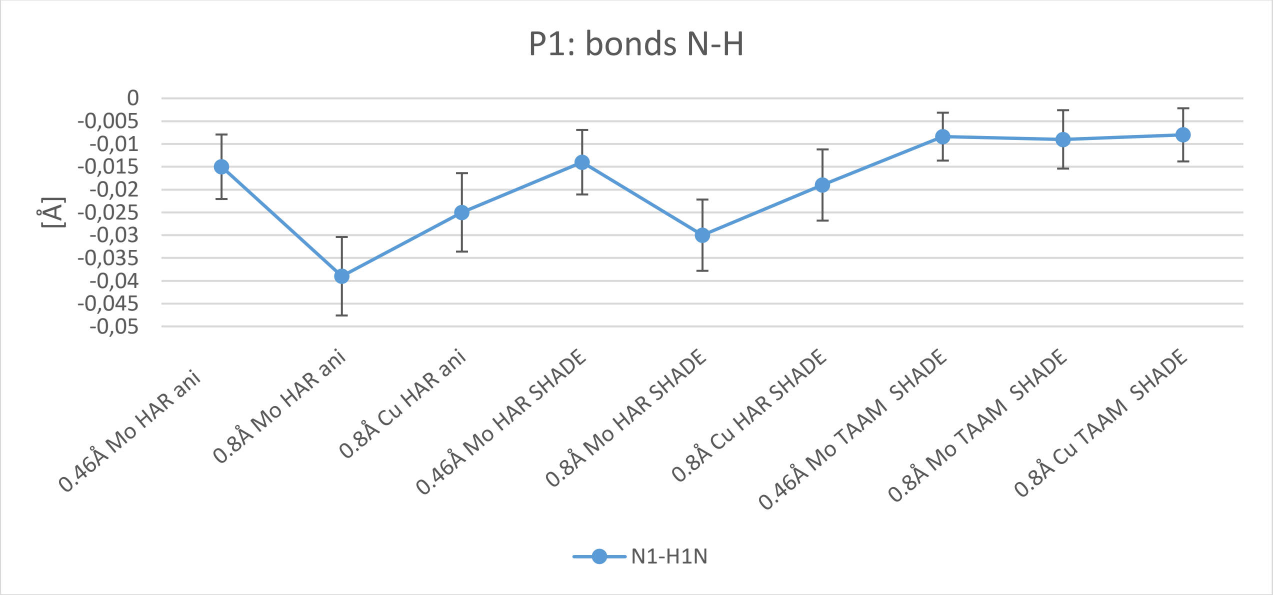

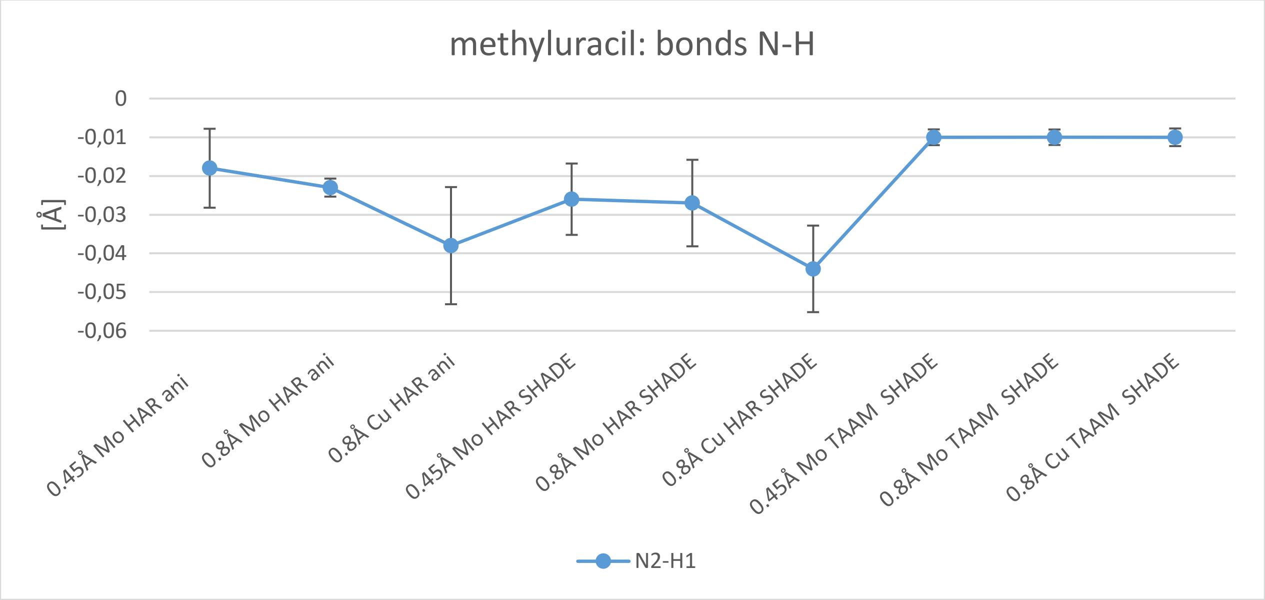


**Figure S35** Comparison of N-H bonds for HAR and TAAM refinements of **3**, **3-cutoff** and **3CuKα**. Values on plot represent difference between values obtained with analysed model and neutron data in angstroms. Lines on plot have no physical meaning, however help in visual analysis. Estimated standard deviations were added to each plot.

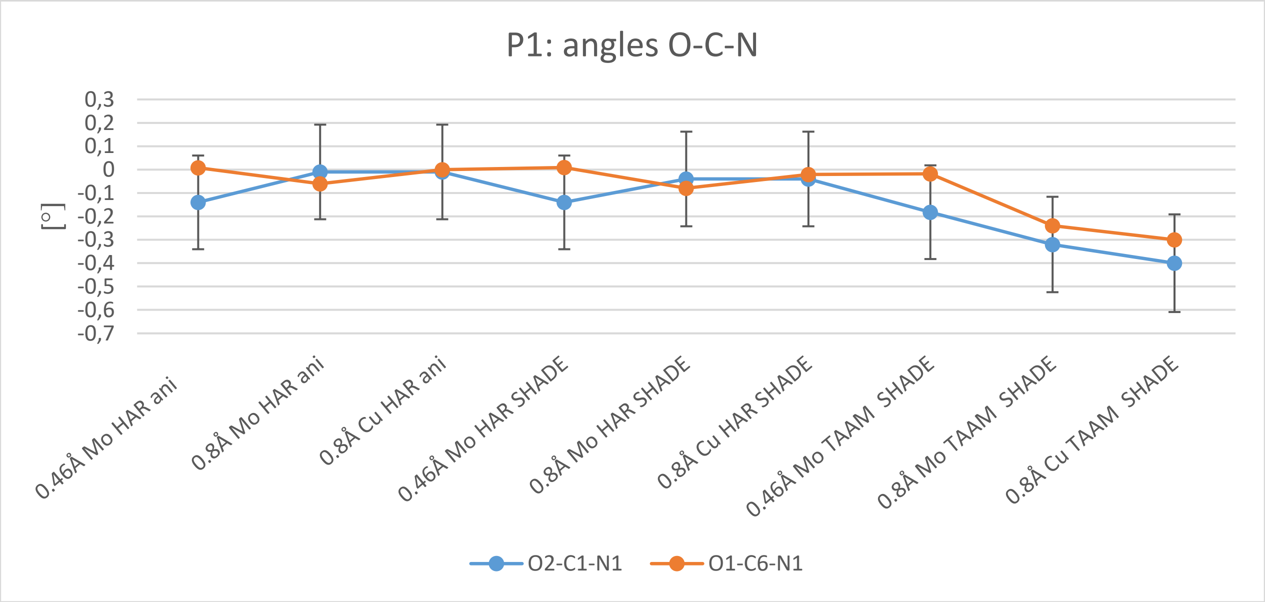

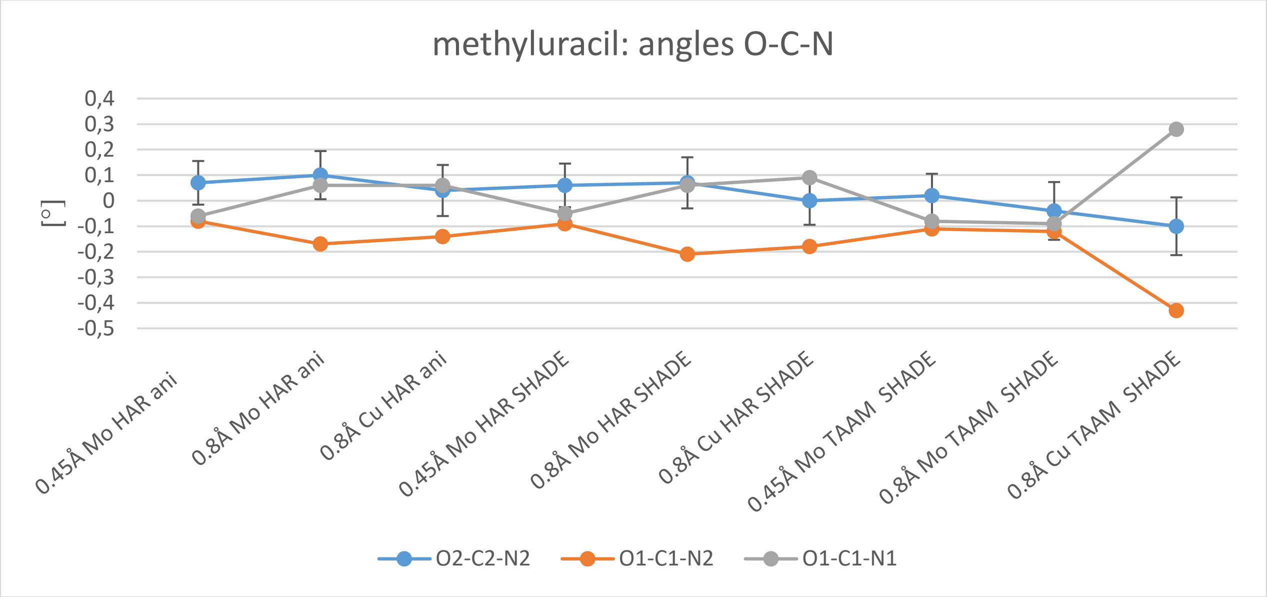


**Figure S36** Comparison of O-C-N angles for HAR and TAAM refinements of **1**, **2**, **3**, **1-cutoff**, **2-cutoff** and **3-cutoff**, **1-CuKα**, **2-CuKα** and **3CuKα**. Values on plot represent difference between values obtained with analysed model and neutron data in angstroms. Lines on plot have no physical meaning, however help in visual analysis. For clarity, estimated standard deviations were added to the selected plots.

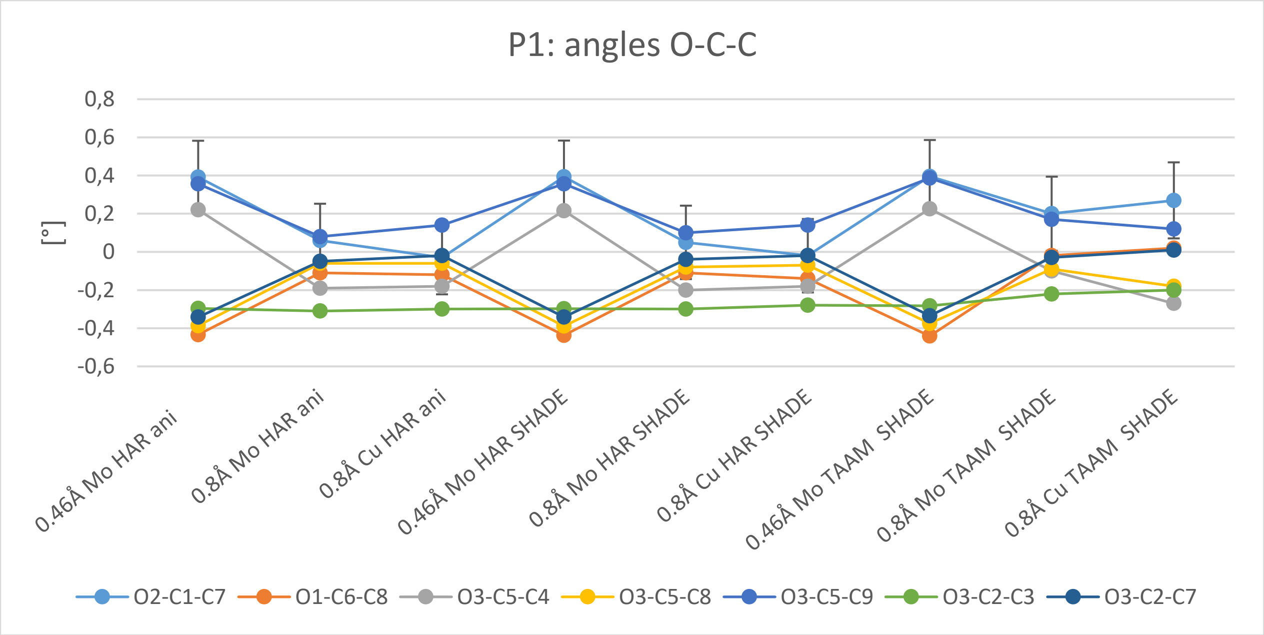

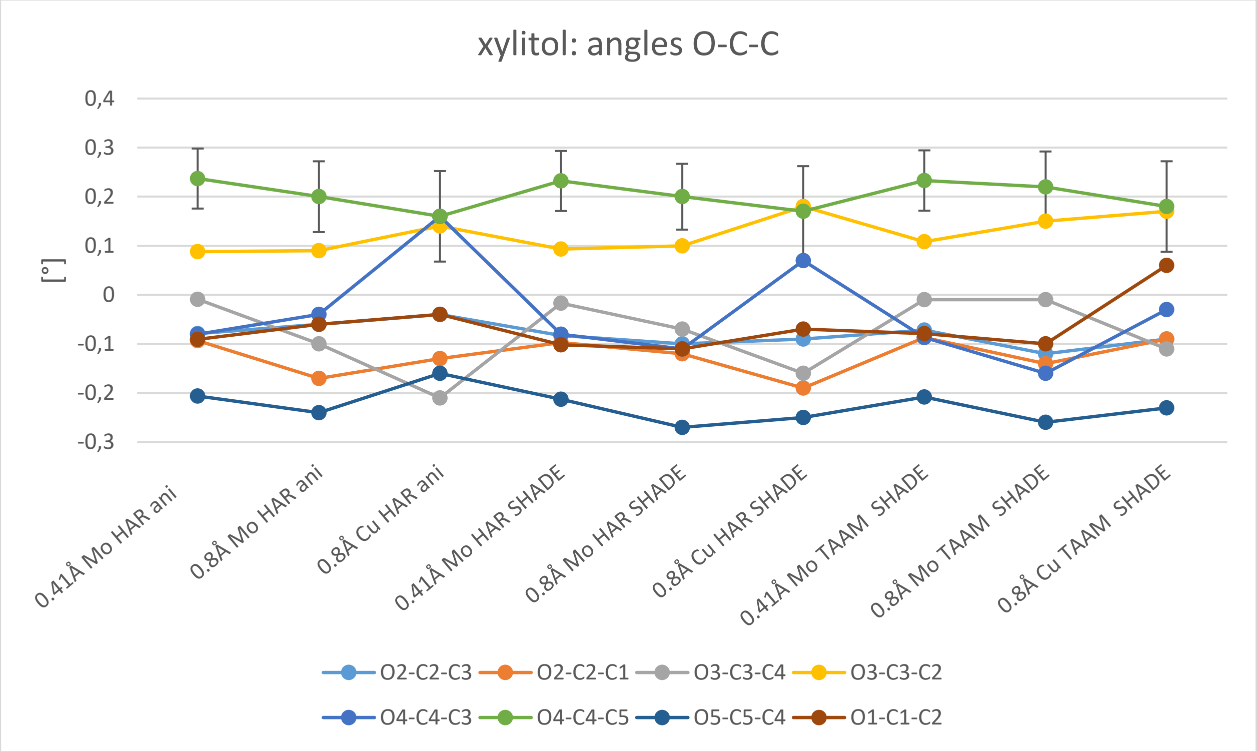


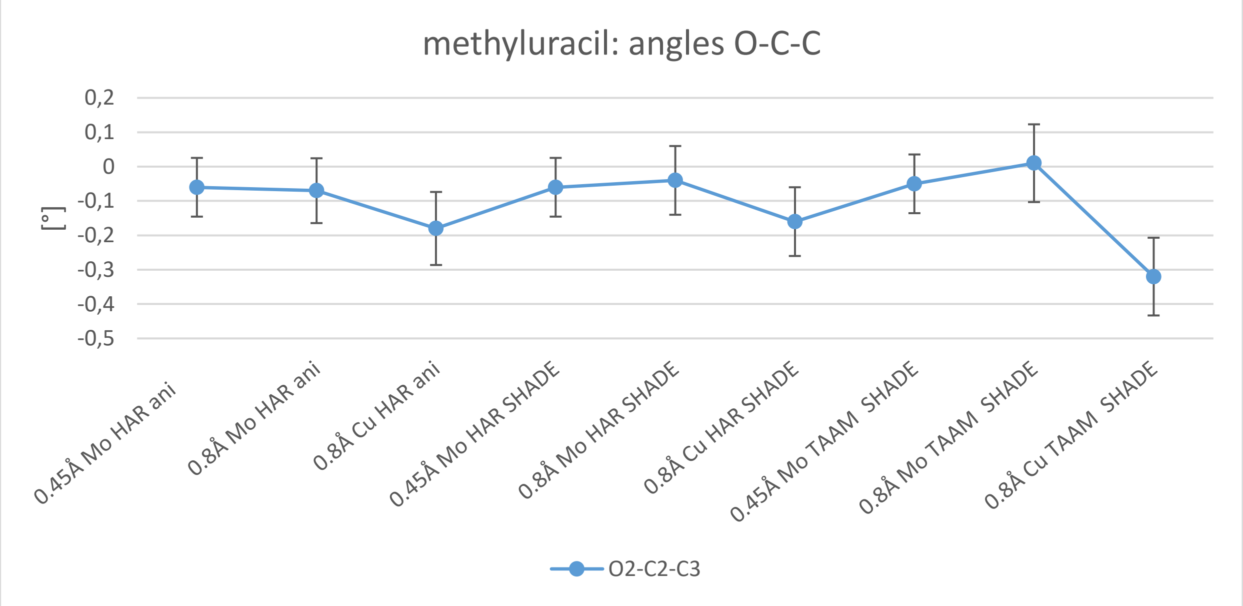


**Figure S37** Comparison of O-C-C angles for HAR and TAAM refinements of **1**, **2**, **3**, **1-cutoff**, **2-cutoff** and **3-cutoff**, **1-CuKα**, **2-CuKα** and **3CuKα**. Values on plot represent difference between values obtained with analysed model and neutron data in angstroms. Lines on plot have no physical meaning, however help in visual analysis. For clarity, estimated standard deviations were added to the selected plots.

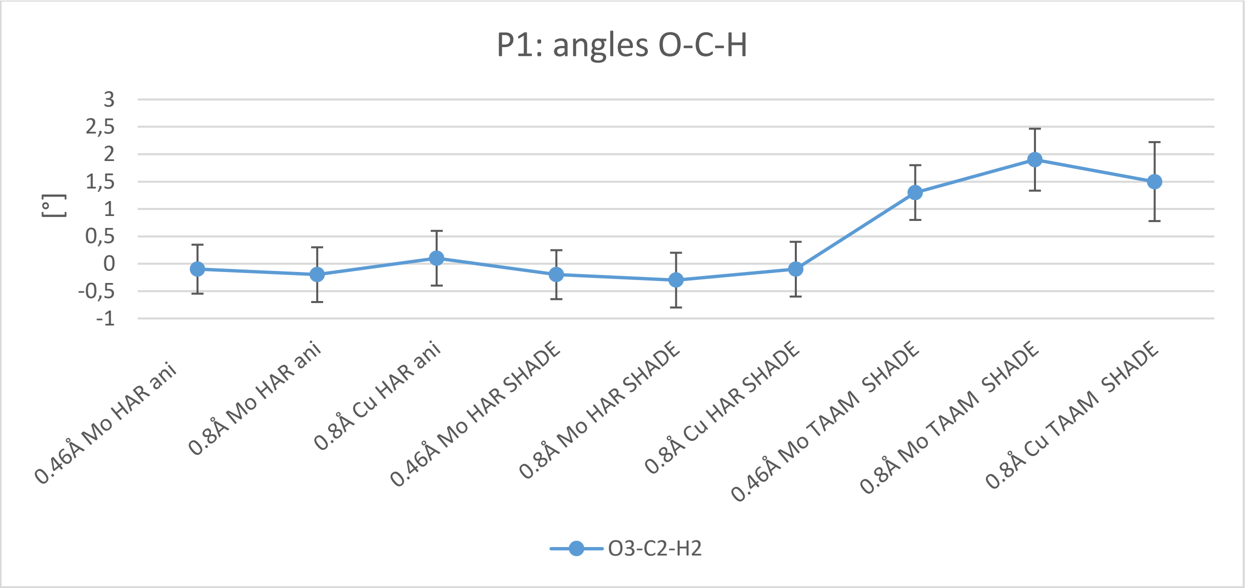


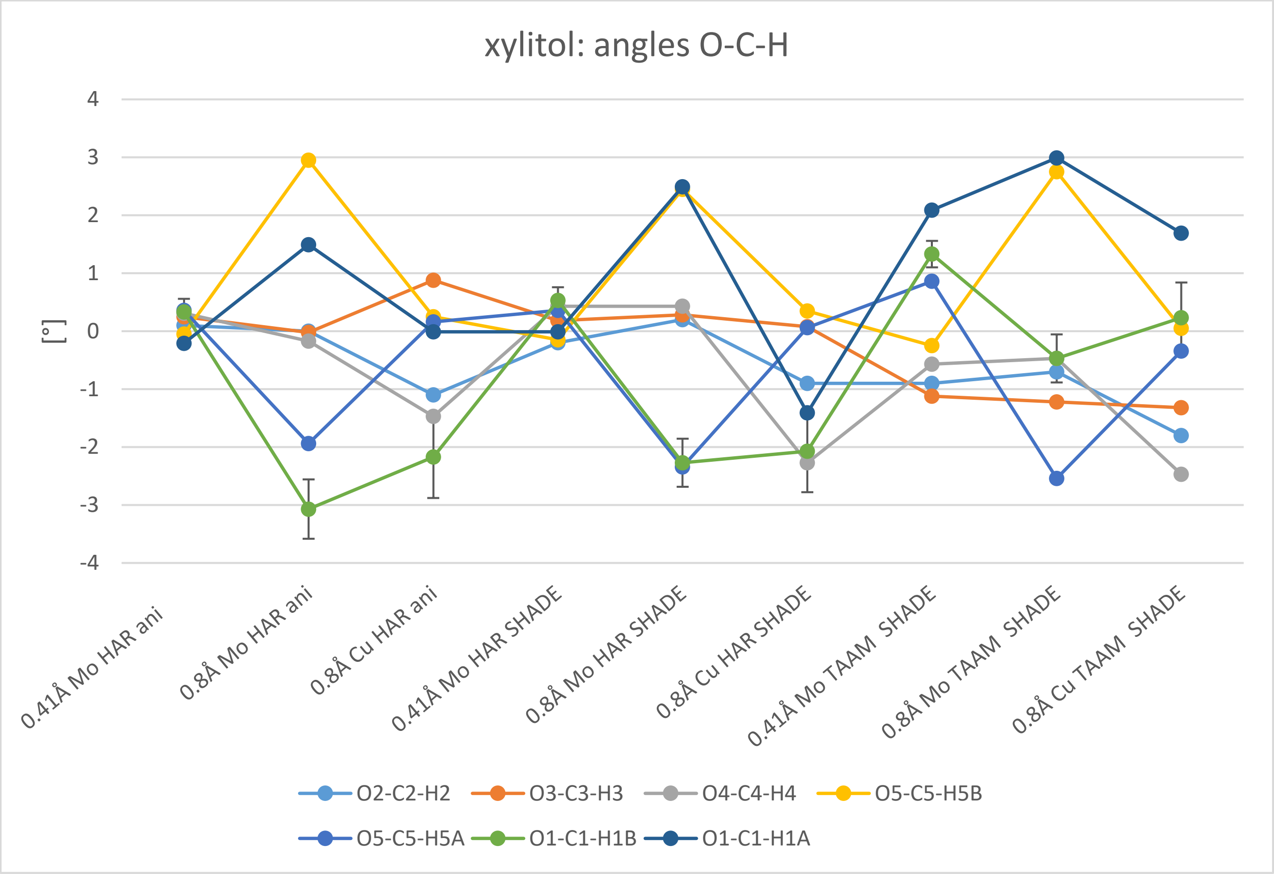


**Figure S38** Comparison of O-C-H angles for HAR and TAAM refinements of **1**, **2**, **1-cutoff**, **2-cutoff**, **1-CuKα** and **2-CuKα**. Values on plot represent difference between values obtained with analysed model and neutron data in angstroms. Lines on plot have no physical meaning, however help in visual analysis. For clarity, estimated standard deviations were added to the selected plots.

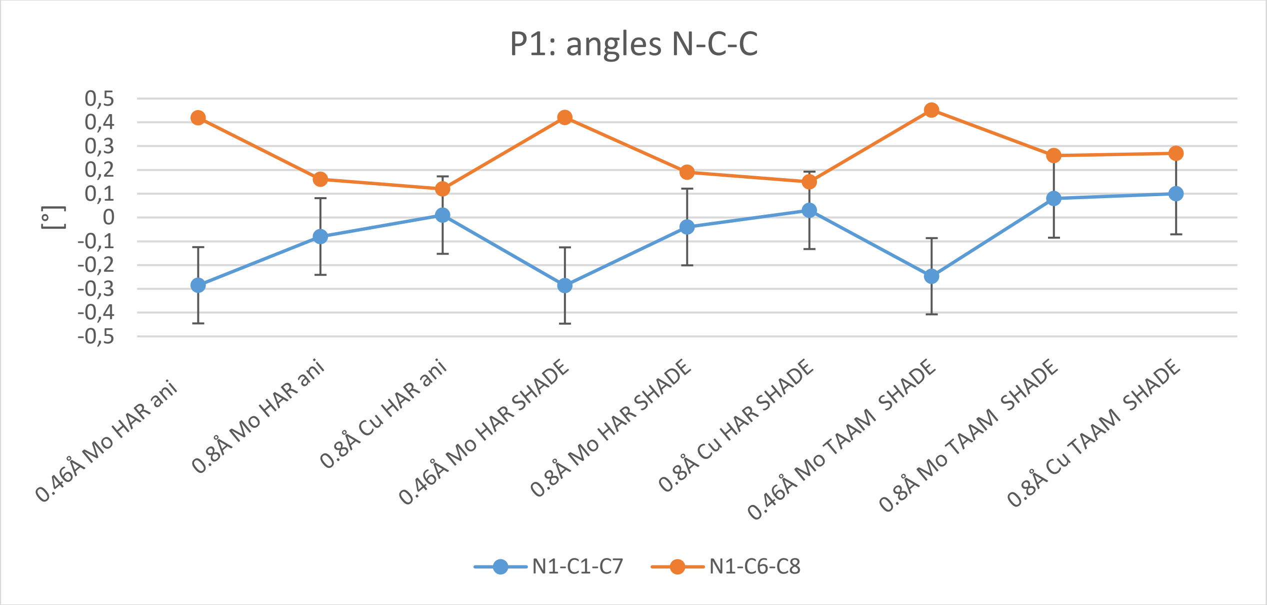


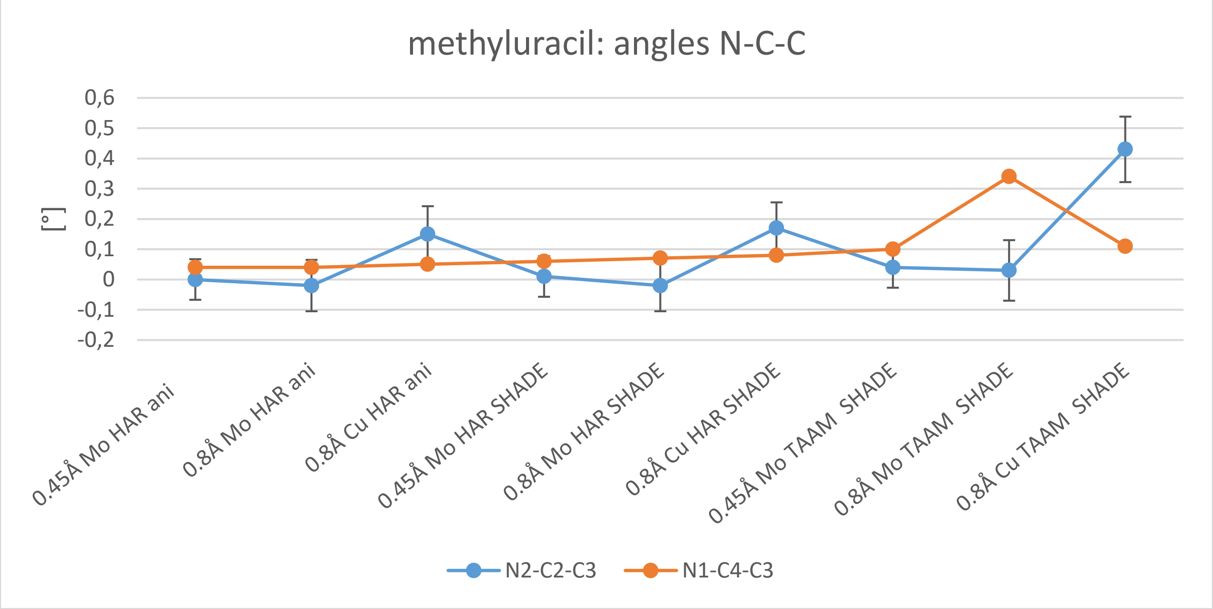


**Figure S39** Comparison of N-C-C angles for HAR and TAAM refinements of **1**, **3**, **1-cutoff**, **3-cutoff**, **1-CuKα** and **3CuKα**. Values on plot represent difference between values obtained with analysed model and neutron data in angstroms. Lines on plot have no physical meaning, however help in visual analysis. For clarity, estimated standard deviations were added to the selected plots.

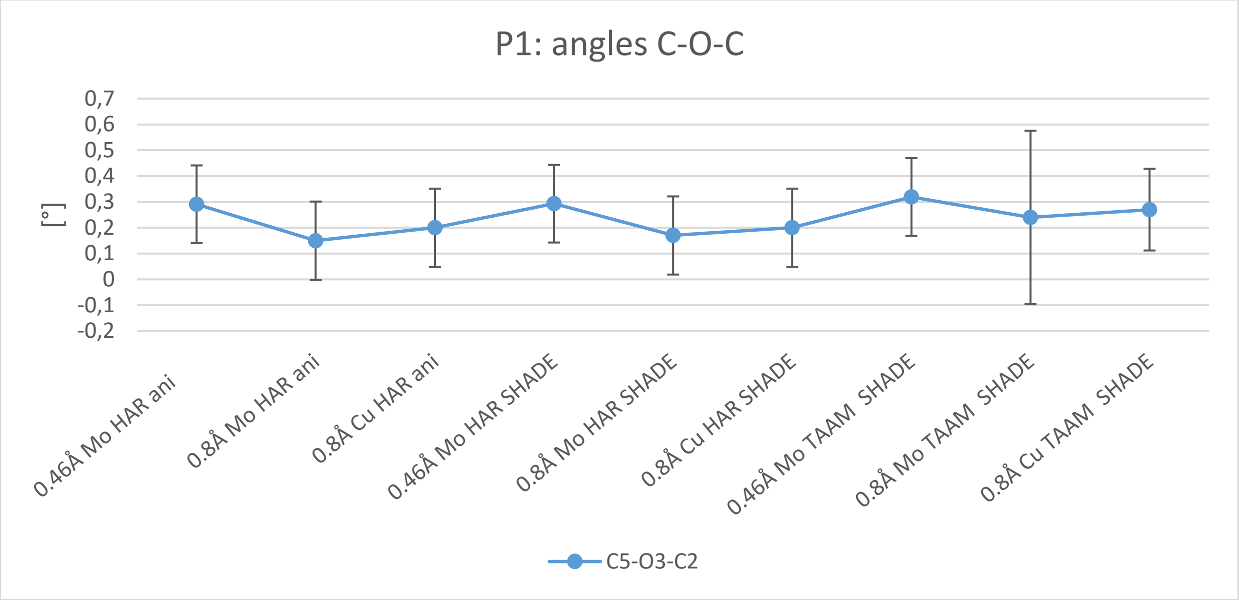


**Figure S40** Comparison of C-O-C angles for HAR and TAAM refinements of **1**, **1-cutoff** and **1-CuKα**. Values on plot represent difference between values obtained with analysed model and neutron data in angstroms. Lines on plot have no physical meaning, however help in visual analysis. Estimated standard deviations were added to the plot.

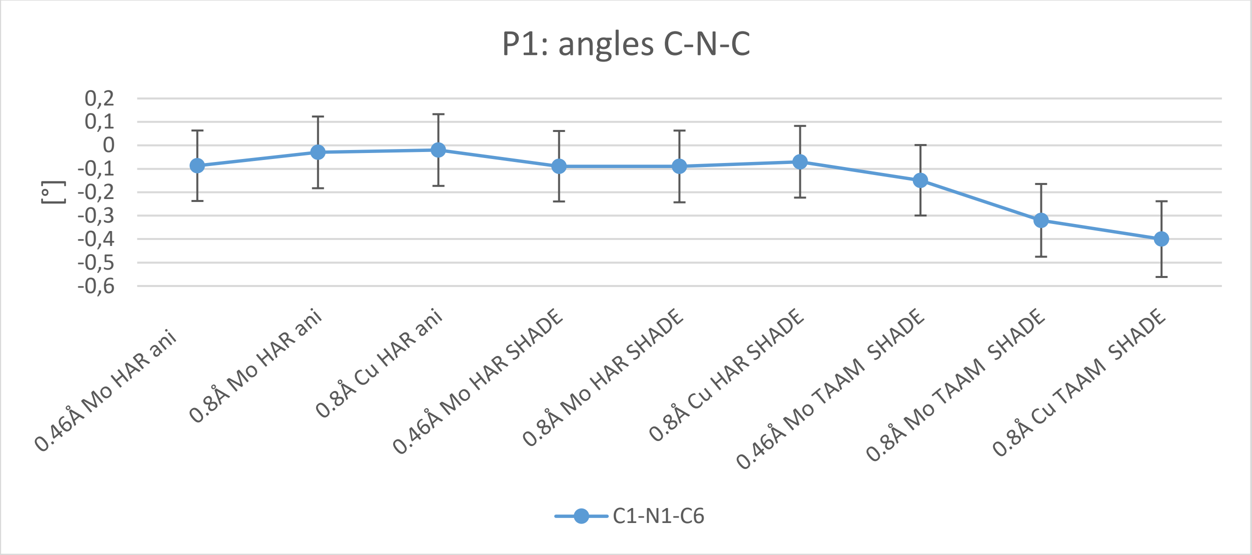


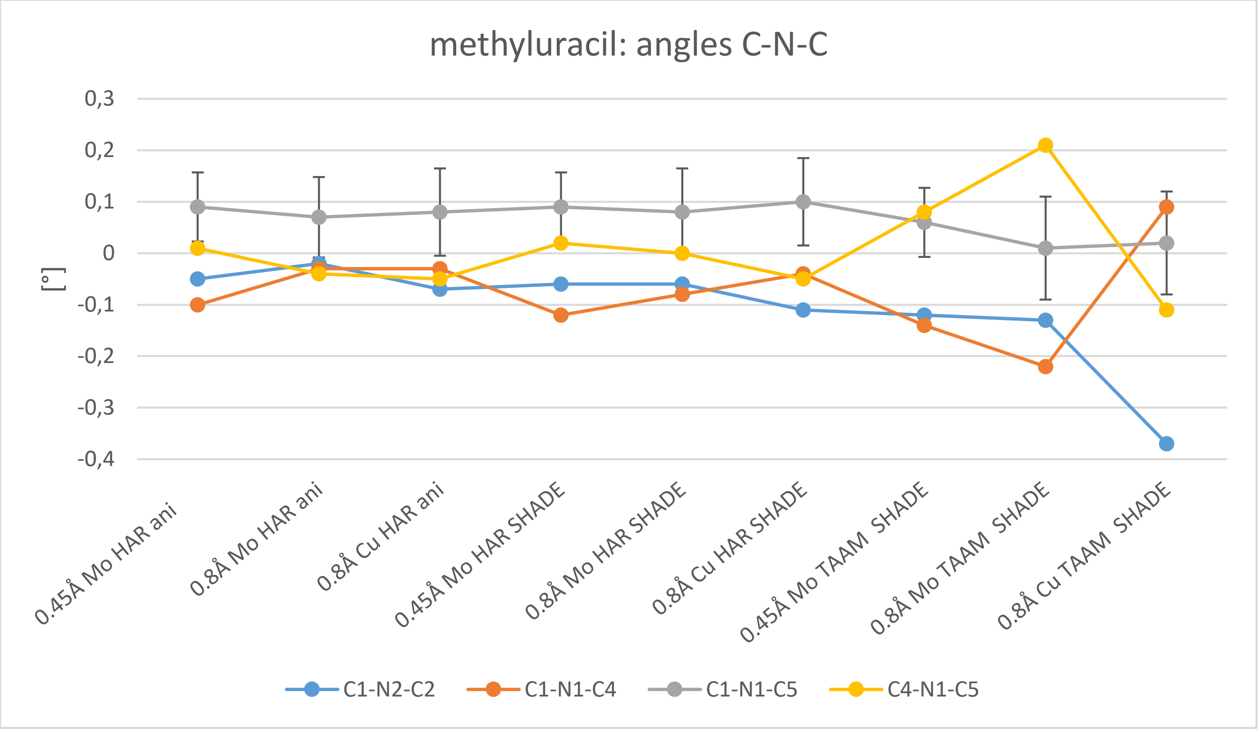


**Figure S41** Comparison of C-N-C angles for HAR and TAAM refinements of **1**, **3**, **1-cutoff**, **3-cutoff**, **1-CuKα** and **3CuKα**. Values on plot represent difference between values obtained with analysed model and neutron data in angstroms. Lines on plot have no physical meaning, however help in visual analysis. For clarity, estimated standard deviations were added to the selected plots.

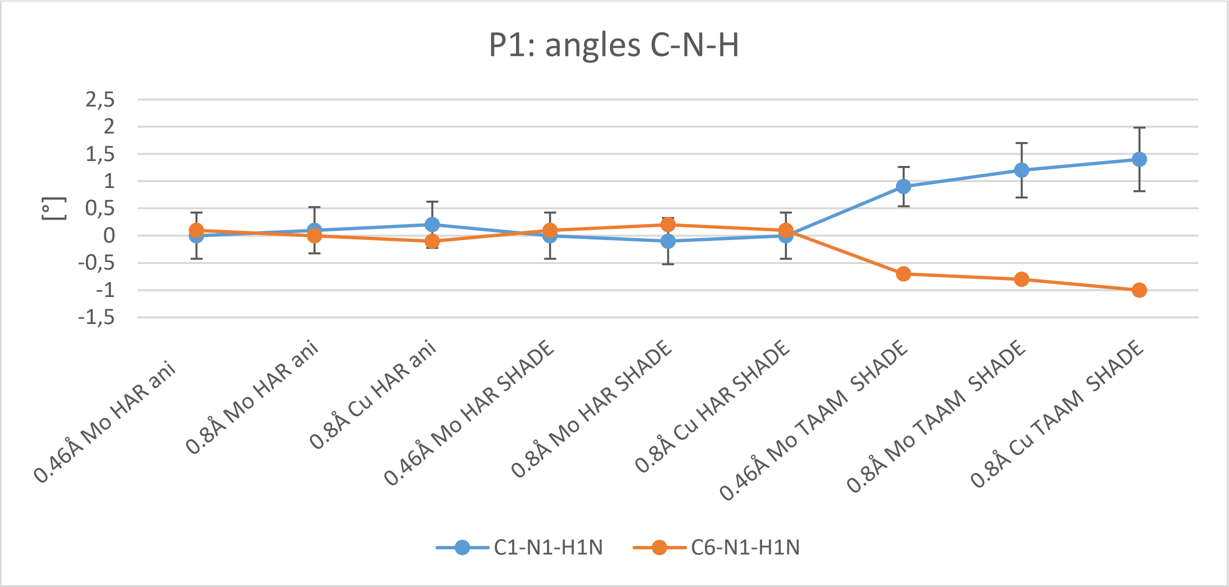


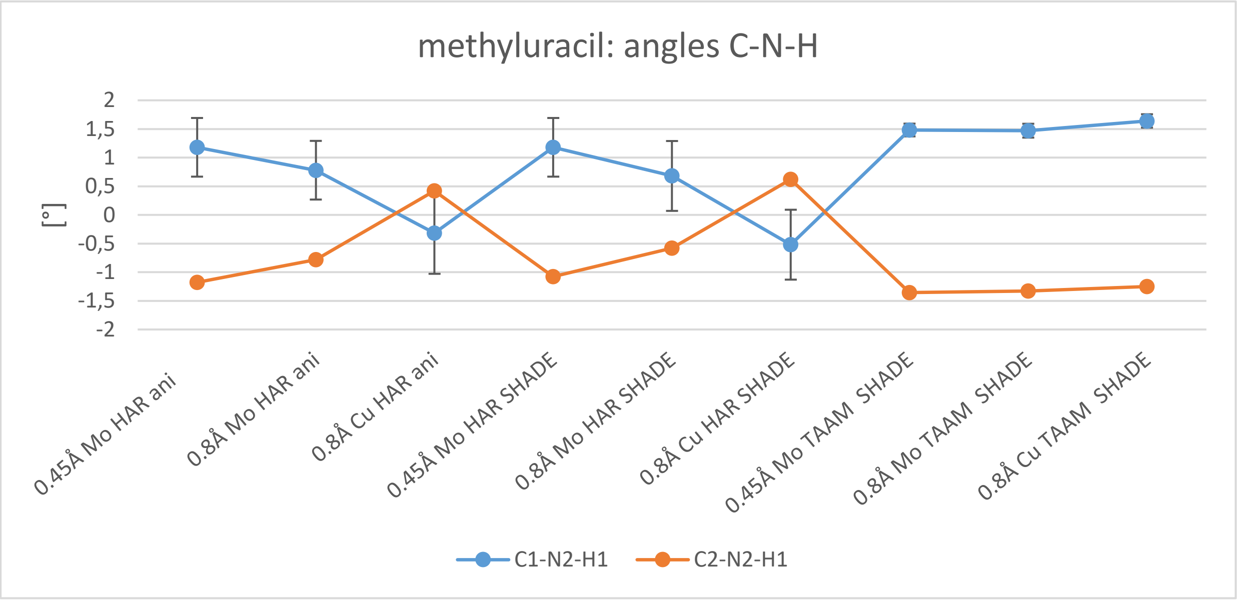


**Figure S42** Comparison of C-N-H angles for HAR and TAAM refinements of **1**, **3**, **1-cutoff**, **3-cutoff**, **1-CuKα** and **3CuKα**. Values on plot represent difference between values obtained with analysed model and neutron data in angstroms. Lines on plot have no physical meaning, however help in visual analysis. For clarity, estimated standard deviations were added to the selected plots.

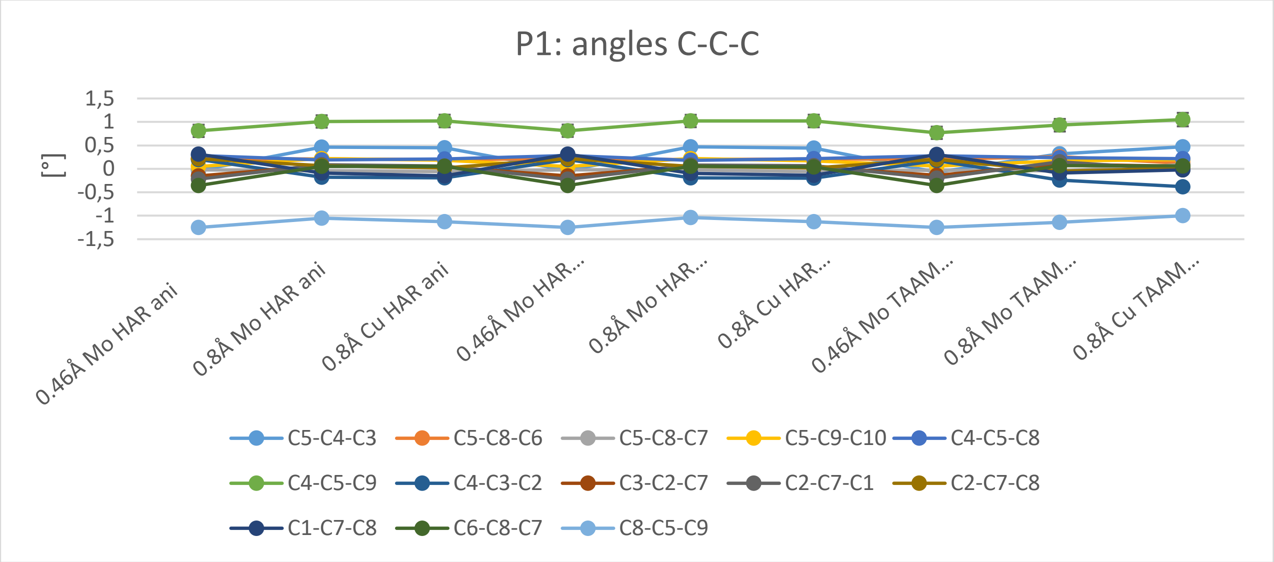
P1: angles C-C-C
1,5
1
0,5
0
-0,5
-1
-1,5
[°]
0.46Å Mo HAR ani
0.8Å Mo HAR ani
0.8Å Cu HAR ani
0.46Å Mo HAR...
0.8Å Mo HAR...
0.8Å Cu HAR...
0.46Å Mo TAAM...
0.8Å Mo TAAM...
0.8Å Cu TAAM...
C5-C4-C3
C5-C8-C6
C5-C8-C7
C5-C9-C10
C4-C5-C8
C4-C5-C9
C4-C3-C2
C3-C2-C7
C2-C7-C1
C2-C7-C8
C1-C7-C8
C6-C8-C7
C8-C5-C9

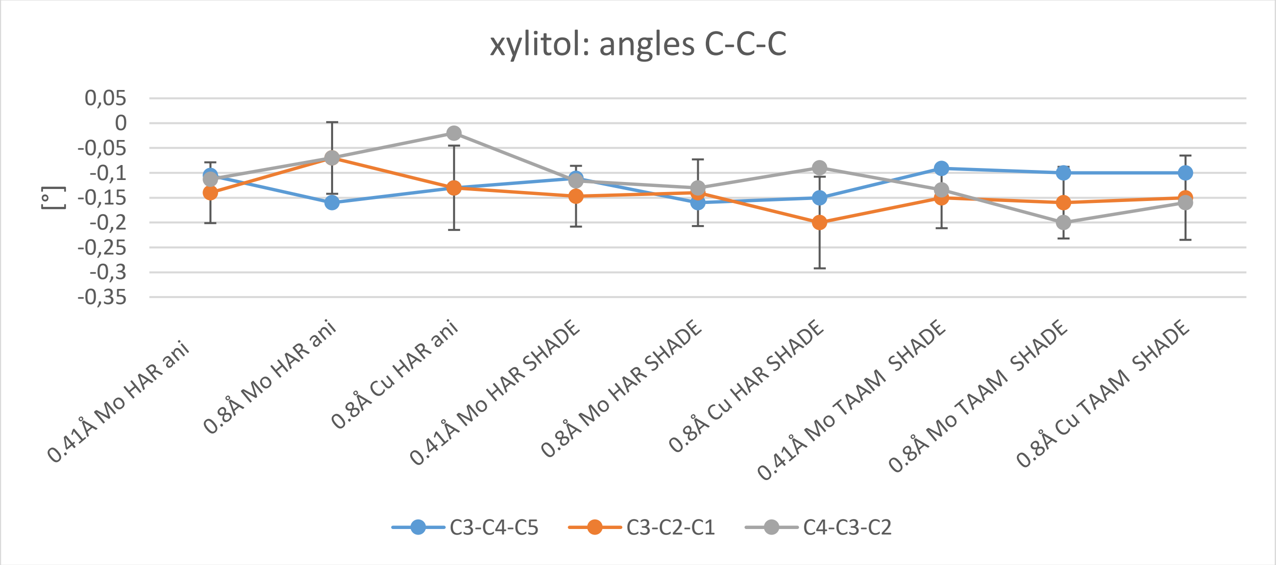
xylitol: angles C-C-C
0,05
0
-0,05
-0,1
-0,15
-0,2
-0,25
-0,3
-0,35
[°]
0.41Å Mo HAR ani
0.8Å Mo HAR ani
0.8Å Cu HAR ani
0.41Å Mo HAR SHADE
0.8Å Mo HAR SHADE
0.8Å Cu HAR SHADE
0.41Å Mo TAAM SHADE
0.8Å Mo TAAM SHADE
0.8Å Cu TAAM SHADE
C3-C4-C5
C3-C2-C1
C4-C3-C2

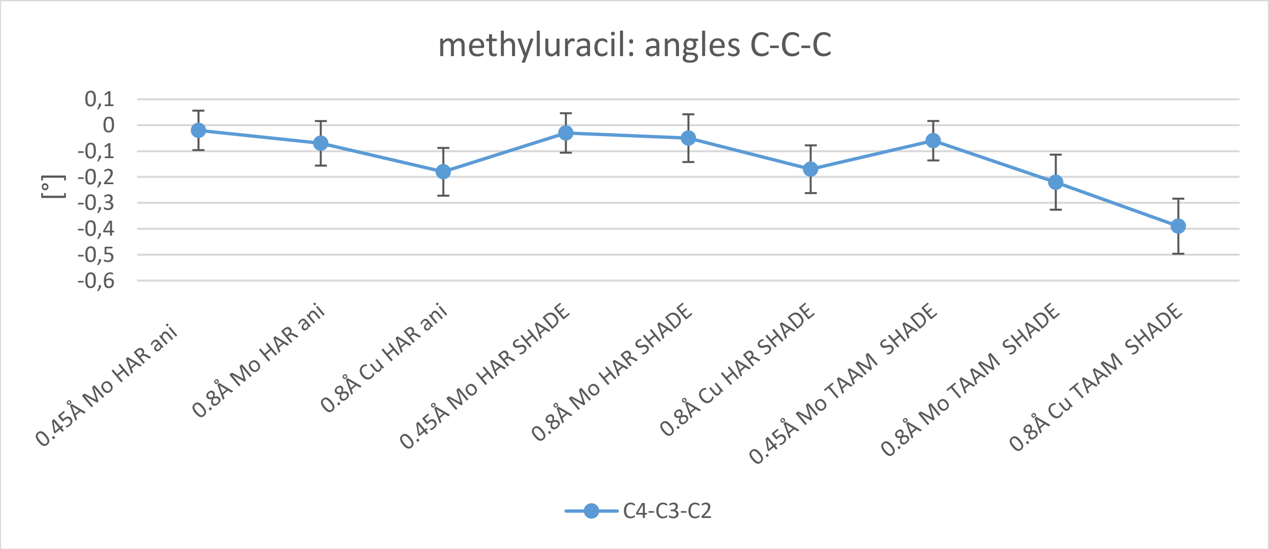
methyluracil: angles C-C-C
0,1
0
-0,1
-0,2
-0,3
-0,4
-0,5
-0,6
[°]
0.45Å Mo HAR ani
0.8Å Mo HAR ani
0.8Å Cu HAR ani
0.45Å Mo HAR SHADE
0.8Å Mo HAR SHADE
0.8Å Cu HAR SHADE
0.45Å Mo TAAM SHADE
0.8Å Mo TAAM SHADE
0.8Å Cu TAAM SHADE
C4-C3-C2

**Figure S43** Comparison of C-C-C angles for HAR and TAAM refinements of **1**, **2**, **3**, **1-cutoff**, **2-cutoff** and **3-cutoff**, **1-CuKα**, **2-CuKα** and **3CuKα**. Values on plot represent difference between values obtained with analysed model and neutron data in angstroms. Lines on plot have no physical meaning, however help in visual analysis. For clarity, estimated standard deviations were added to the selected plots.

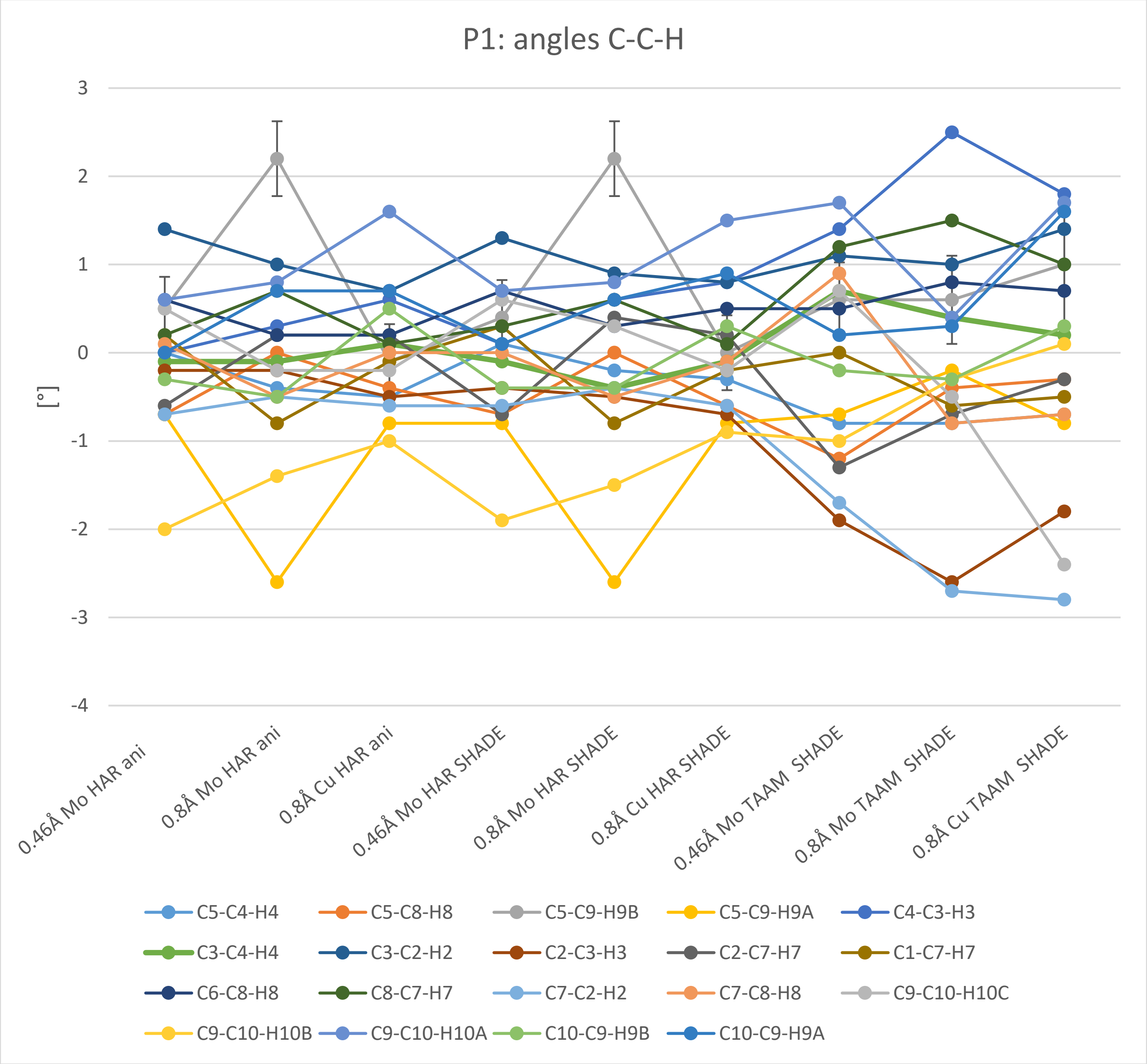

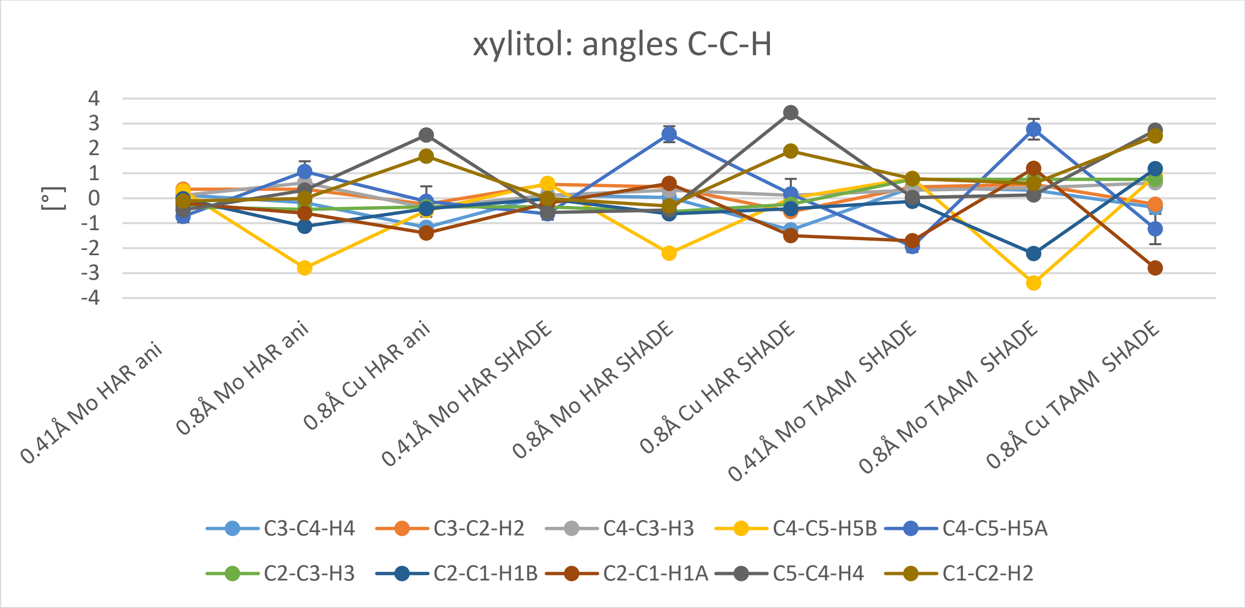


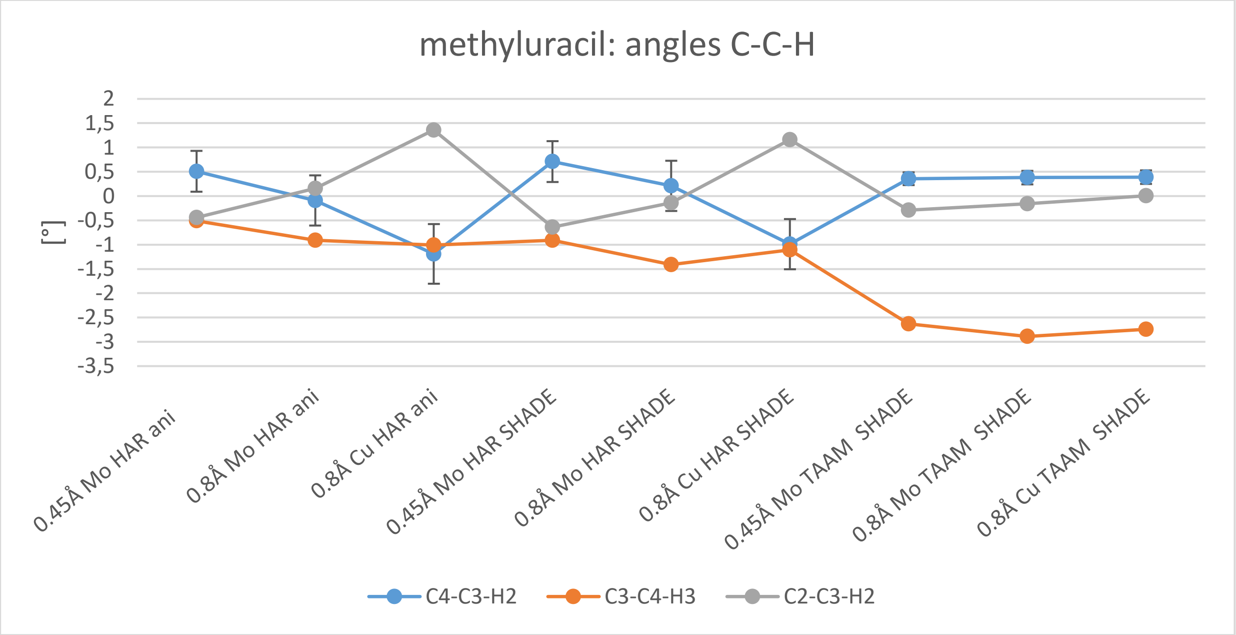


**Figure S44** Comparison of C-C-H angles for HAR and TAAM refinements of **1**, **2**, **3**, **1-cutoff**, **2-cutoff** and **3-cutoff**, **1-CuKα**, **2-CuKα** and **3CuKα**. Values on plot represent difference between values obtained with analysed model and neutron data in angstroms. Lines on plot have no physical meaning, however help in visual analysis. For clarity, estimated standard deviations were added to the selected plots.

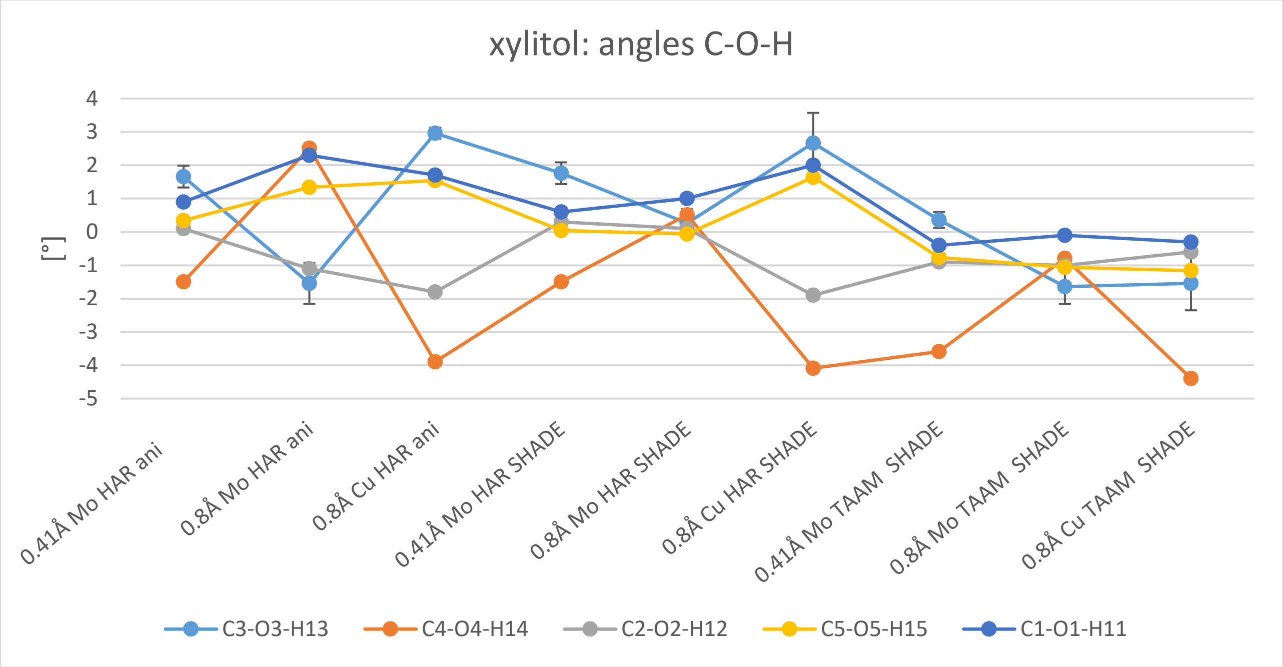


**Figure S45** Comparison of C-O-H angles for HAR and TAAM refinements of **2**, **2-cutoff** and **2-CuKα**. Values on plot represent difference between values obtained with analysed model and neutron data in angstroms. Lines on plot have no physical meaning, however help in visual analysis. For clarity, estimated standard deviations were added to the selected plots.

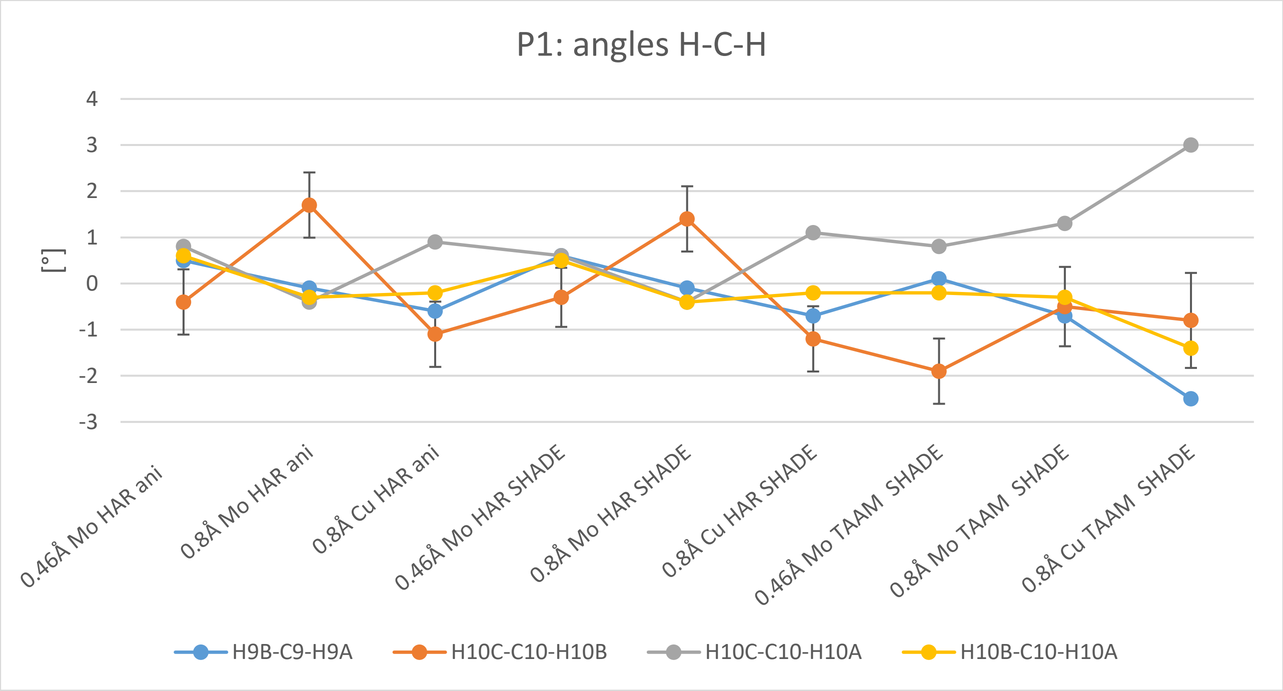

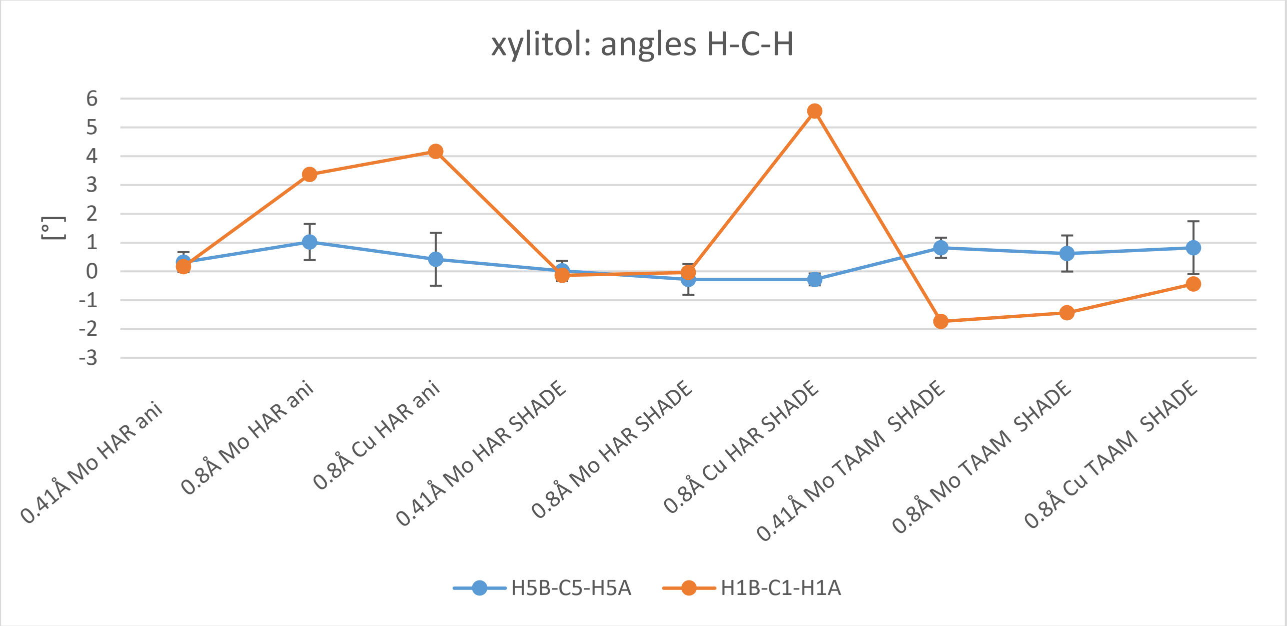


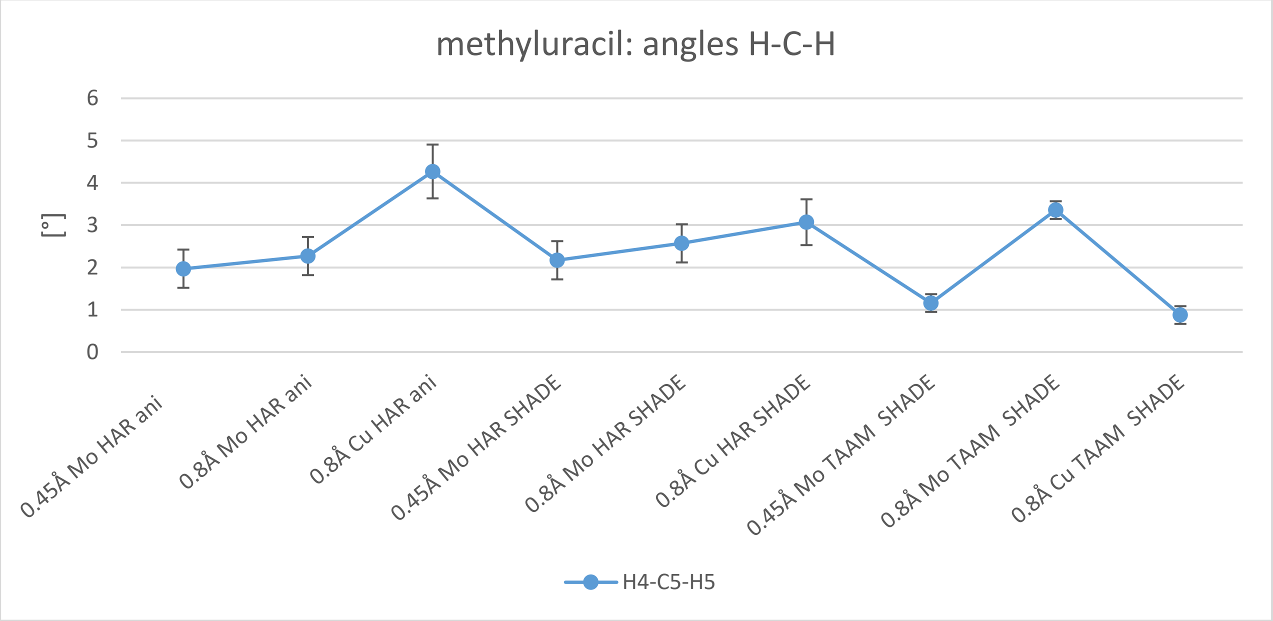


**Figure S46** Comparison of H-C-H angles for HAR and TAAM refinements of **1**, **2**, **3**, **1-cutoff**, **2-cutoff** and **3-cutoff**, **1-CuKα**, **2-CuKα** and **3CuKα**. Values on plot represent difference between values obtained with analysed model and neutron data in angstroms. Lines on plot have no physical meaning, however help in visual analysis. For clarity, estimated standard deviations were added to the selected plots.

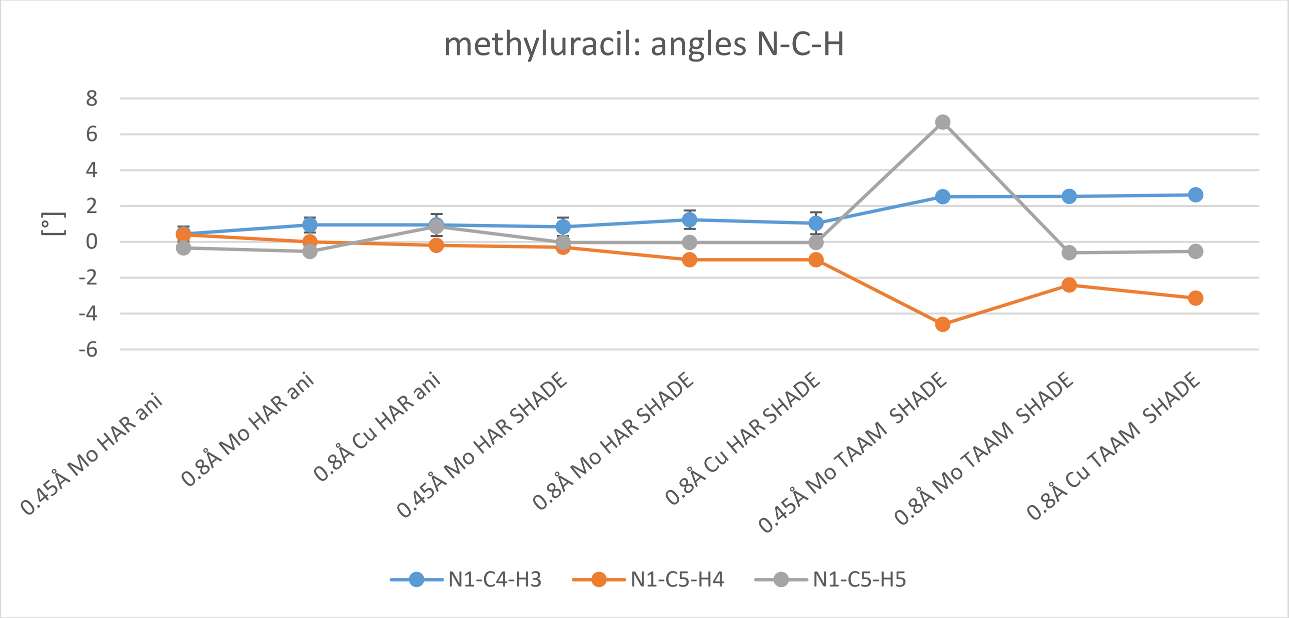


**Figure S47** Comparison of N-C-H angles for HAR and TAAM refinements of **3**, **3-cutoff** and **3CuKα**. Values on plot represent difference between values obtained with analysed model and neutron data in angstroms. Lines on plot have no physical meaning, however help in visual analysis. For clarity, estimated standard deviations were added to the selected plots.

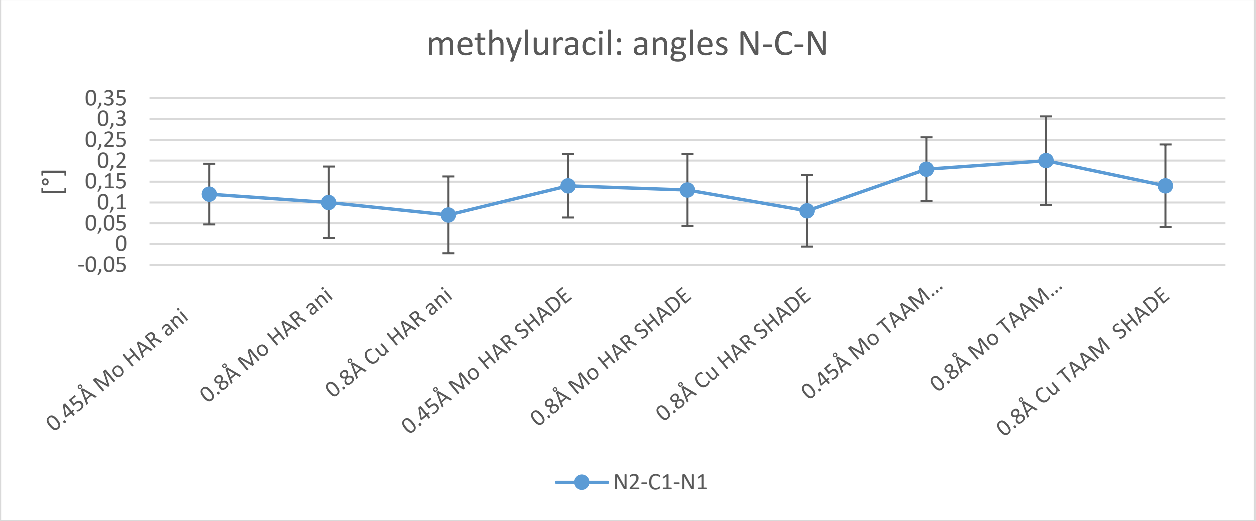


**Figure S48** Comparison of N-C-H angles for HAR and TAAM refinements of **3**, **3-cutoff** and **3CuKα**. Values on plot represent difference between values obtained with analysed model and neutron data in angstroms. Lines on plot have no physical meaning, however help in visual analysis. Estimated standard deviations were added to the plot.

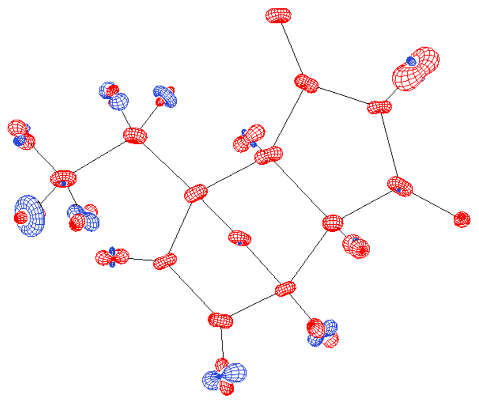

**1-cutoff**: HAR aniso

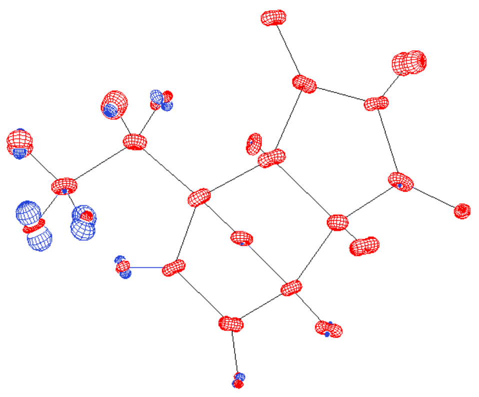

**1-cutoff**: HAR SHADE

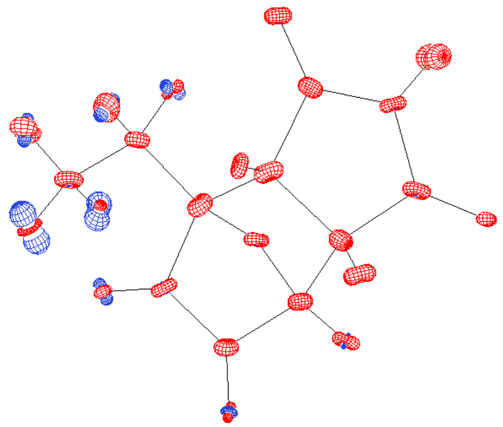

**1-cutoff**: TAAM SHADE

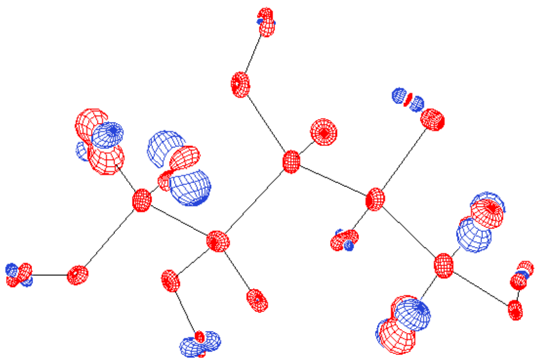

**2-cutoff**: TAAM SHADE

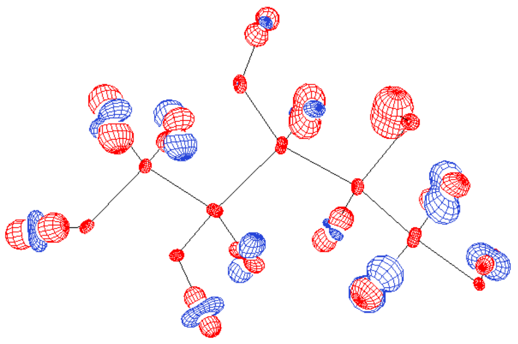

**2-cutoff**: HAR aniso

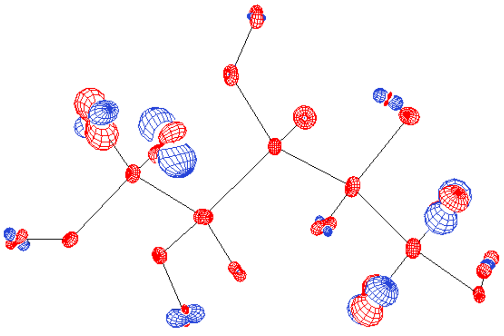

**2-cutoff**: HAR SHADE

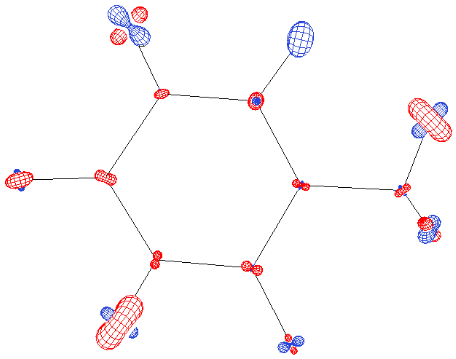

**3-cutoff**: HAR aniso

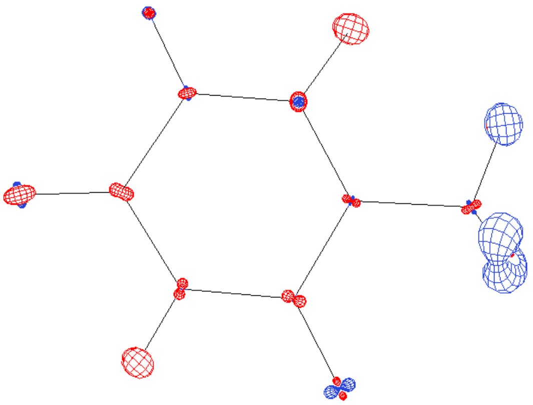

**3-cutoff**: HAR SHADE

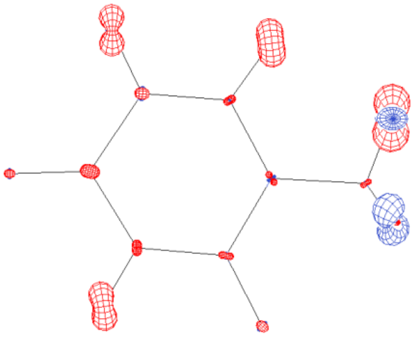

**3-cutoff**: TAAM SHADE

**Figure S49** Difference between ADPs of neutron and analysed model for HAR and TAAM refinements of **1-cutoff**, **2-cutoff** and **3-cutoff**. Two-step overlay algorithm and 2.0 scale were applied using PEANUT software.

## Section S1. Combination of Normal Mode Refinement with the Multipole Model

Starting from TAAM structure of **1** ([10] CSD deposition number: 2008296 (1)) the high resolution X-ray diffraction data for **1** were refined using Mo-ProSuite. [11] In the refinement, the following parameters were refined consecutively: valence populations ($P_{val}$) multipoles ($P_{lm}$, *i.e.* dipoles, quadrupoles, octupoles and for oxygen atoms hexadecapoles), expansion and contraction parameters (kappa and kappa'). After each block refinement of kappas, the xyz and Uij parameters were re-fined. As the ADPs of H-atoms were obtained from the Normal Mode Refinement (procedure described in the Section 2.4 in ms. and Scheme S3 in SI). Moreover, the X-H distances were extended to the mean neutron values as restraints with a standard deviation of 0.001. [12]Final structure is present in the Fig. S50 (in the middle) and statistical parameters are present in the Table S14.  Based on the geometry of the final structure, statistical parameters, fractal dimension plot and residual density maps (Fig. S51) we can judge the **1MM_NoMoRe** structure as a reliable. However, we excluded detail analysis of this approach in the ms. as the NoMoRe ADPs were already analysed in the Section 3.3. in ms. and the restraints were applied for the X-H bonds. Therefore, the influence on X-H bonds in the MM_NoMoRe refinement is not significant as in the case of HAR_NoMoRe refinement whereas the X-H bonds are refined freely (Fig. S52). However, based on the residual density maps (Fig. S51) we can see the differences arise from the ADPs modelling on the electron density. Furthermore, combination of both refinements i.e. MM and NoMoRe were already reported [9].

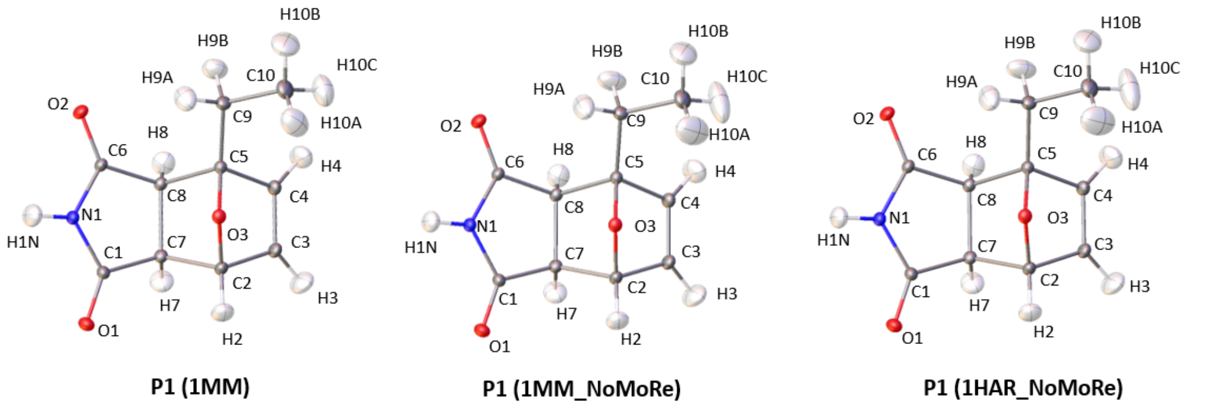


**Figure S50** Labelling of atoms and visualisation and comparison of atomic ADPs in molecular structures of the studied compound **1** obtained with the MM, MM_NoMoRe and HAR_NoMoRe refinements. Anisotropic atomic displacement ellipsoids are drawn at the 50% probability level.

**Table S14** Statistical parameters of MM NoMoRe refinement of **1** with comparison to the MM and HAR NoMoRe.

| Compound | Parameters | MM | MM NoMoRe | HAR NoMoRe |
|---|---|---|---|---|
| **1** | R[F > 2σ(F)] | 0.013 | 0.014 | 0.015 |
| | $wR(F^2)$ | 0.043 | 0.043 | 0.045 |
| | # of reflections | 9849 | 9849 | 9828 |
| | # of fit parameters | 160 | 160 | 160 |
| | $chi^2$ | n/a | n/a | 2.41 |
| | Goodness of fit | 1.35 | 1.34 | 1.55 |
| | $\Delta\varrho_{max}$ | 0.22 | 0.22 | 0.21 |
| | $\Delta\varrho^{min}$ | -0.16 | -0.15 | -0.15 |

**1MM**

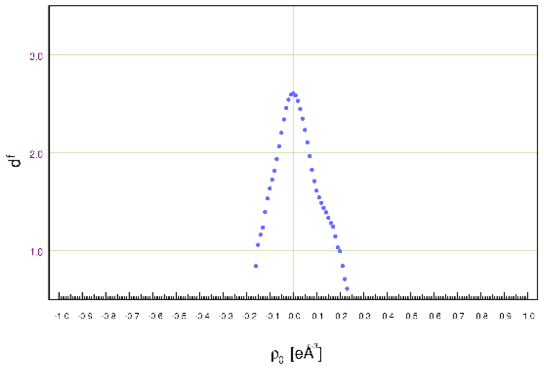


**1MM_NoMoRe**

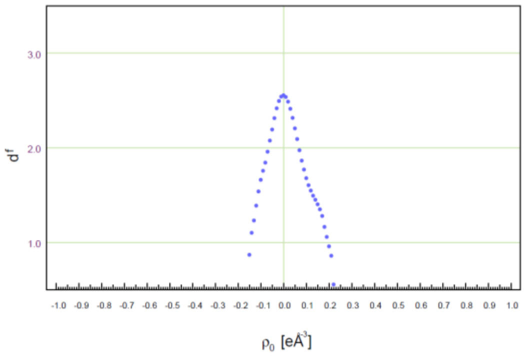


Fractal dimension plots.

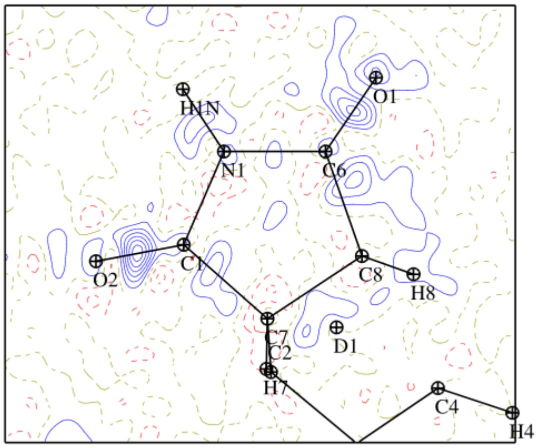


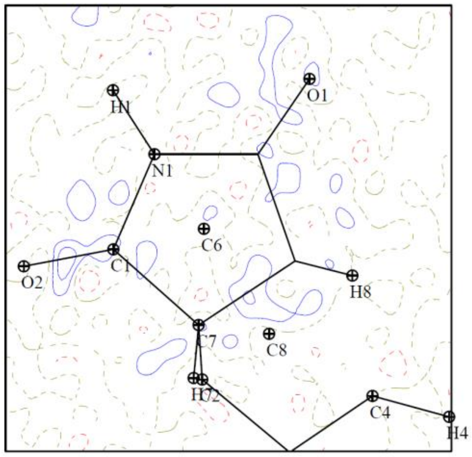


Residual density maps, plane determined by N1 (centre), C6 (x axis) and C1 (y axis) atoms. Maps are presented with contours levels with intervals ±0.05 eÅ$^{-3}$. Blue lines represent positive values and red lines the negative ones

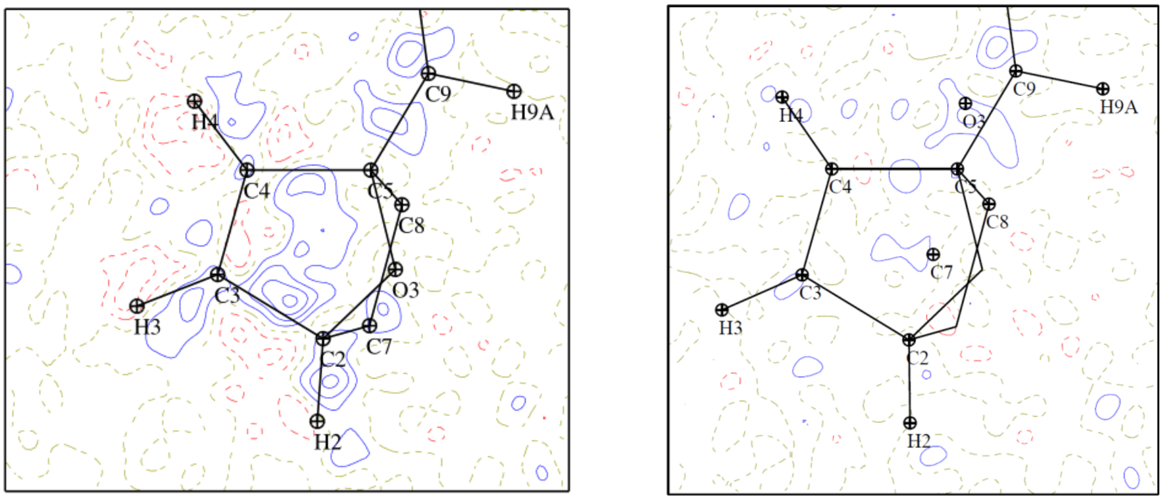


Residual density maps, plane determined by C4 (centre), C5 (x axis) and C3 (y axis) atoms. Maps are presented with contours levels with intervals ±0.05 eÅ$^{-3}$. Blue lines represent positive values and red lines the negative ones

**Figure S51** Fractal dimension plots and residual density maps for MM (SHADE, NoMoRe) refinements of **1**.

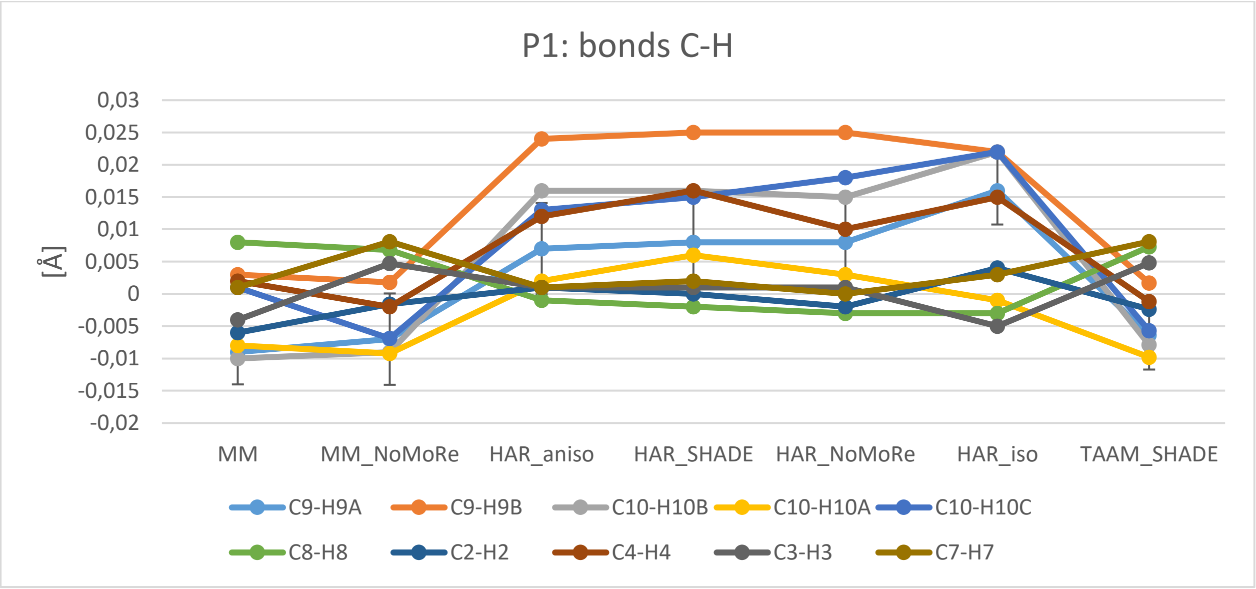


**Figure S52** Comparison of C-H bonds for MM (SHADE, NoMoRe), HAR (SHADE, NoMoRe, aniso, iso) and TAAM (SHADE) refinements of **1**. Values on plot represent difference between values obtained

with analysed model and neutron data in angstroms. Lines on plot have no physical meaning, however help in visual analysis. For clarity, estimated standard deviations were added to selected plot (C9-H9A).

**References:**


1. Capelli, S.C.; Bürgi, H.-B.; Dittrich, B.; Grabowsky, S.; Jayatilaka, D. Hirshfeld Atom Refinement. *IUCrJ* **2014**, *1*, 361–379, doi:10.1107/S2052252514014845.
2. Jarzembska, K.N.; Dominiak, P.M. New Version of the Theoretical Databank of Transferable Aspherical Pseudoatoms, UBDB2011 – towards Nucleic Acid Modelling. *Acta Cryst A* **2012**, *68*, 139–147, doi:10.1107/S0108767311042176.
3. Jelsch, C.; Pichon-Pesme, V.; Lecomte, C.; Aubry, A. Transferability of Multipole Charge-Density Parameters: Application to Very High Resolution Oligopeptide and Protein Structures. *Acta Cryst D* **1998**, *54*, 1306–1318, doi:10.1107/S0907444998004466.
4. Dittrich, B.; Munshi, P.; Spackman, M.A. Invariom-Model Refinement of l-Valinol. *Acta Cryst C* **2006**, *62*, o633–o635, doi:10.1107/S0108270106037358.
5. Dittrich, B.; Hübschle, C.B.; Pröpper, K.; Dietrich, F.; Stolper, T.; Holstein, J. The Generalized Invariom Database (GID). *Acta Cryst B* **2013**, *69*, 91–104, doi:10.1107/S2052519213002285.
6. Domagała, S.; Fournier, B.; Liebschner, D.; Guillot, B.; Jelsch, C. An Improved Experimental Databank of Transferable Multipolar Atom Models – ELMAM2. Construction Details and Applications. *Acta Cryst A* **2012**, *68*, 337–351, doi:10.1107/S0108767312008197.
7. Bąk, J.M.; Domagała, S.; Hübschle, C.; Jelsch, C.; Dittrich, B.; Dominiak, P.M. Verification of Structural and Electrostatic Properties Obtained by the Use of Different Pseudoatom Databases. *Acta Cryst A* **2011**, *67*, 141–153, doi:10.1107/S0108767310049731.
8. Hoser, A.A.; Madsen, A.Ø. Dynamic Quantum Crystallography: Lattice-Dynamical Models Refined against Diffraction Data. I. Theory. *Acta Cryst A* **2016**, *72*, 206–214, doi:10.1107/S2053273315024699.
9. Hoser, A.A.; Madsen, A.Ø. Dynamic Quantum Crystallography: Lattice-Dynamical Models Refined against Diffraction Data. II. Applications to l-Alanine, Naphthalene and Xylitol. *Acta Cryst A* **2017**, *73*, 102–114, doi:10.1107/S2053273316018994.
10. Wanat, M.; Malinska, M.; Gutmann, M.J.; Cooper, R.I.; Wozniak, K. HAR, TAAM and BODD Refinements of Model Crystal Structures Using Cu Kα and Mo Kα X-Ray Diffraction Data. *Acta Cryst B* **2021**, *77*, doi:10.1107/S2052520620014936.
11. Jelsch, C.; Guillot, B.; Lagoutte, A.; Lecomte, C. Advances in Protein and Small-Molecule Charge-Density Refinement Methods Using MoPro. *J Appl Cryst* **2005**, *38*, 38–54, doi:10.1107/S0021889804025518.
12. Allen, F.H.; Bruno, I.J. Bond Lengths in Organic and Metal-Organic Compounds Revisited: X—H Bond Lengths from Neutron Diffraction Data. *Acta Cryst B* **2010**, *66*, 380–386, doi:10.1107/S0108768110012048.
